# Supplementary material for: Massively parallel screen uncovers many rare 3′ UTR variants regulating mRNA abundance of cancer driver genes
Source: Nat Commun. 2024 Apr 18;15:3335. doi: 10.1038/s41467-024-46795-7 (PMC11026479; doi:10.1038/s41467-024-46795-7)
Supplement: Supplementary file 1 — Supplementary Information [file 41467_2024_46795_MOESM1_ESM.pdf]

Supplementary Information

Massively parallel screen uncovers many rare 3' UTR variants regulating mRNA abundance of cancer driver genes

Ting Fu, Kofi Amoah, Tracey W. Chan, Jae Hoon Bahn, Jae-Hyung Lee, Sari Terrazas, Rockie Chong, Sriram Kosuri, Xinshu Xiao

Supplementary Figures 1-17
Supplementary Tables 1-3
Supplementary Method
Supplementary Note 1. Off-target results for prime editing experiments
Supplementary Note 2. Plasmid map

a

Control SNPs	Expected RNA/DNA Ratio
chr4:74963310|*CXCL2*|-|alternate|C	>1
chr4:74963329|*CXCL2*|-|alternate|A	<1
chr6:152420197|*ESR1*|+|alternate|T	<1

b

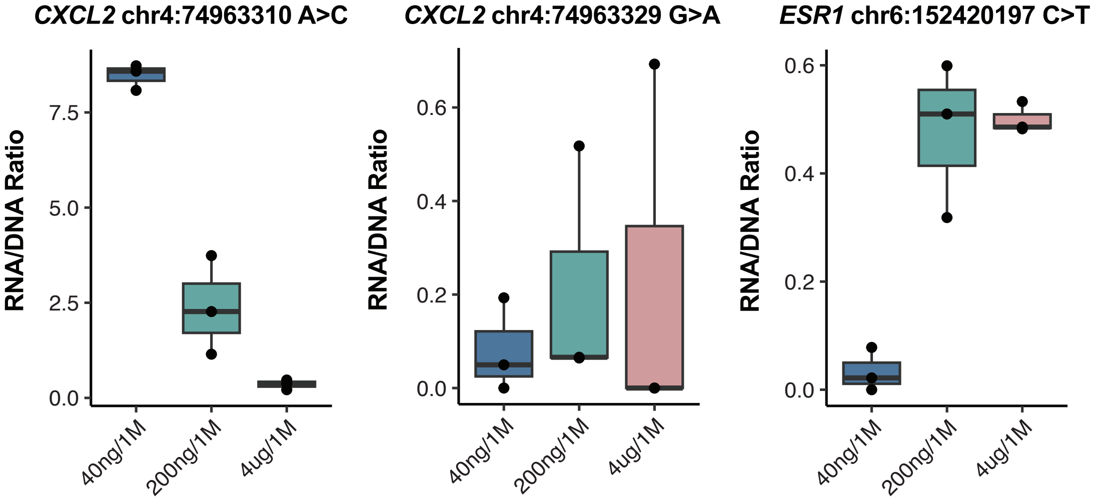

Fig. S1 l DNA/Cell ratio optimization using cell electroporation.
a, A list of control SNPs used for optimization tests. **b**, RNA/DNA ratios observed for the alternative allele of the control variants when transfected under different DNA (ng)/Cell (M) ratios. N=3 biological replicates were included in each experiment. Boxplots are plotted as median, the 25% and 75% percentiles, and non-outlier maxima and minima. Source data are provided as a Source Data file.

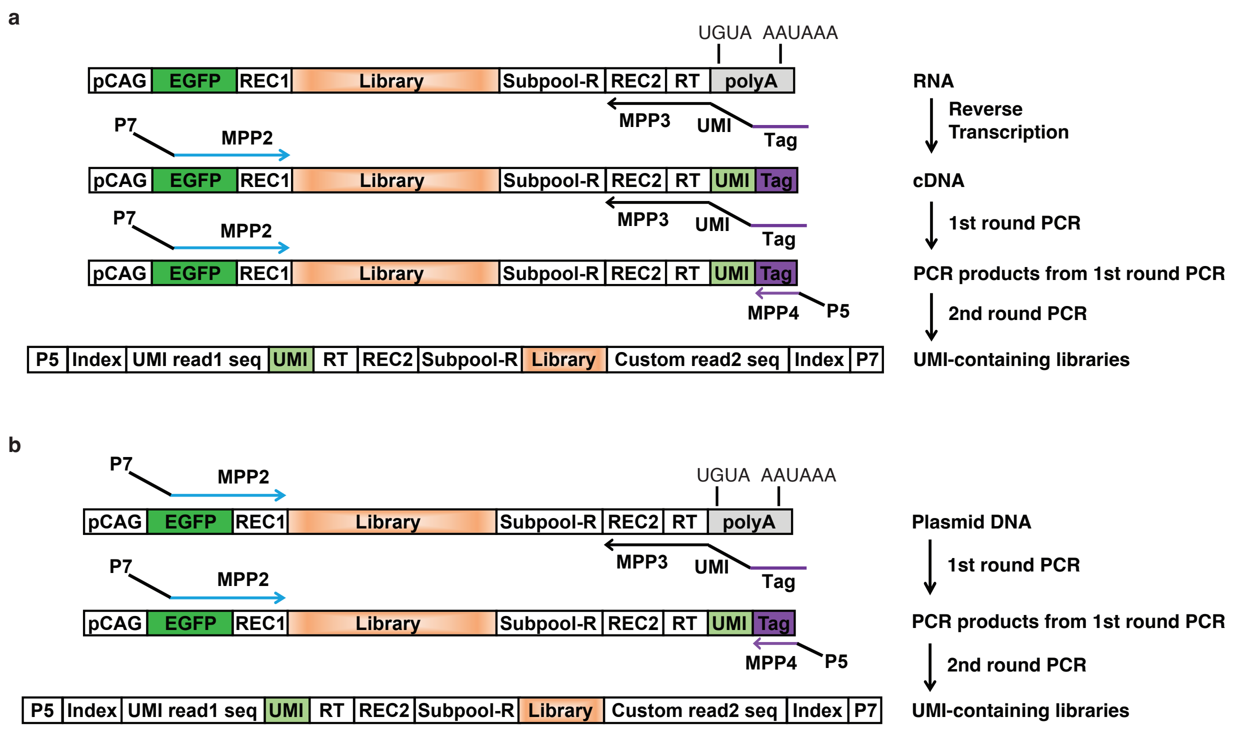

Fig. S2 l Generation of UMI-containing libraries.
a, RNA-seq library generation for mRNA isolated from HEK293/HeLa cells electroporated with plasmid libraries. A polyA signal (AAUAAA) and a UGUA motif 69nt upstream of the polyA signal were labeled in the diagram. **b**, DNA-seq library generation for plasmid libraries used for cell electroporation.

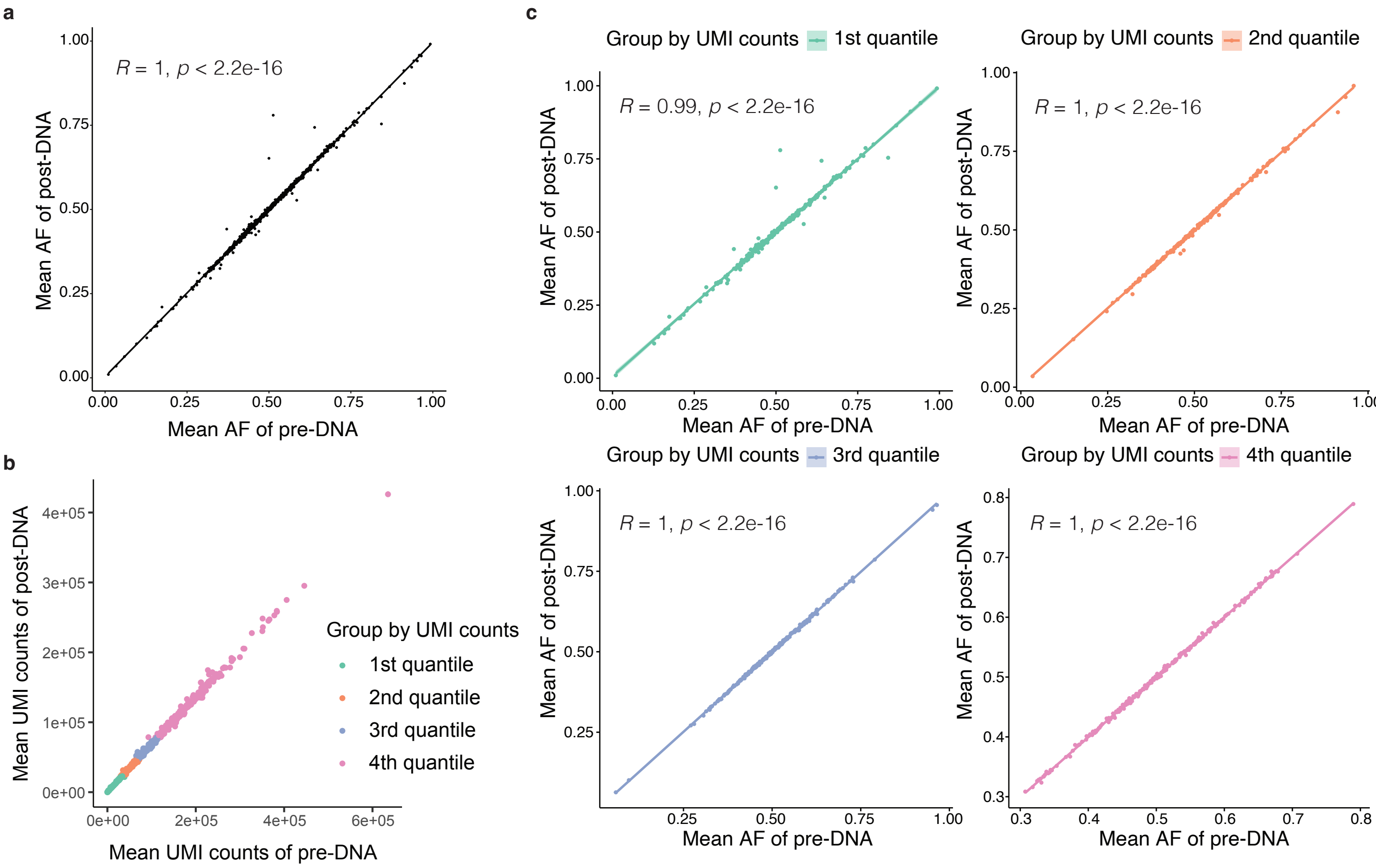

Fig. S3 l Comparison between post- and pre- transfected DNA for RNA normalization.
a, Spearman correlation of alternative allele frequencies (alt/(alt+ref)) between pre- and post- transfected DNA. **b**, Scatter plot of mean total counts (alt + ref) between pre- and post- transfected DNA separated into 4 quantiles. The 1st quantile represents the group of variants with the least amount of UMI counts. The 4th quantile represents the highest UMI counts. **c**, Spearman correlation of alternative allele frequencies (alt/(alt+ref)) between pre- and post- transfected DNA in different quantiles defined in **b**. **a-c**, N=3 biologically independent experiments. Source data are provided as a Source Data file.

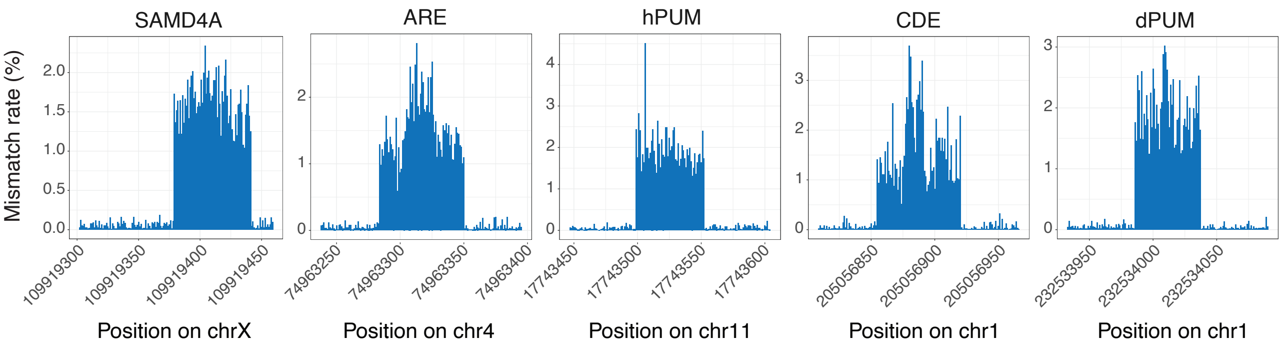

Fig. S4 | MapUTR sequencing quality and accuracy.
Mismatch rate (%) per position along the length of RNA sequences harboring known motifs: SAMD4A, sterile alpha motif domain containing 4A motif (in gene *CHRDL1*), ARE, AU-rich element (in gene *CXCL2*), hPUM, human pumilio motif (in gene *MYOD1*), CDE, constitutive decay element (in gene *RBBP5*), and dPUM, *Drosophila* pumilio motif (in gene *SIPA1L2*). Source data are provided as a Source Data file.

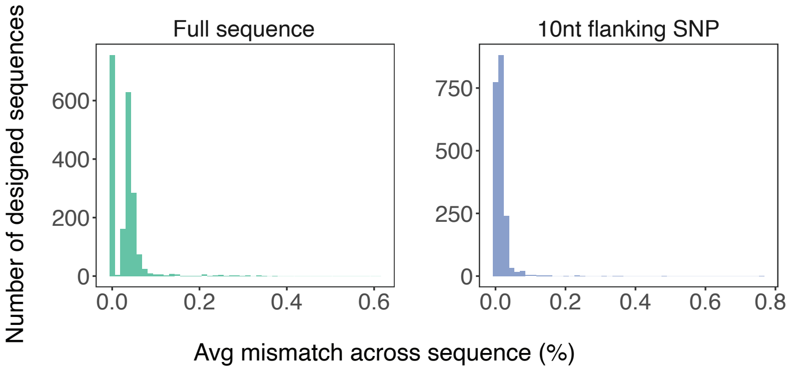

Fig. S5 | Mismatch rates of designed sequences.
Distribution of mismatch rates across the full length (left) and in the local vicinity (+/- 10nt, right) of the designed sequences. Source data are provided as a Source Data file.

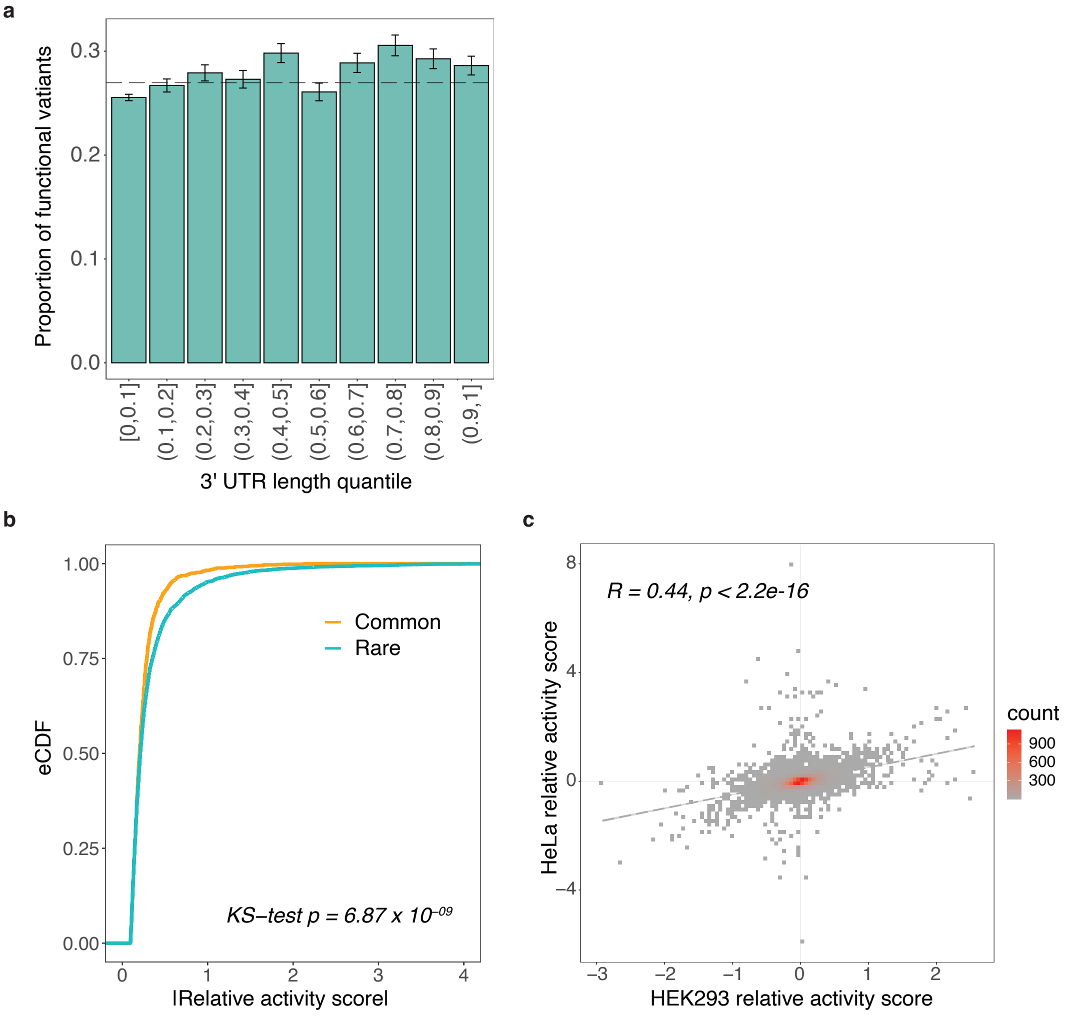

Fig. S6 | MapUTR identifies functional gnomAD rare variants along the 3' UTR regions with larger effect sizes compared to common variants.

a, Proportion of tested variants that are functional in various regions along the length of the 3' UTR. The regional distribution is not different from a uniform distribution (two-sided KS-test goodness of fit p = 0.998). **b**, Absolute effect sizes of functional rare and common variants tested with MapUTR in HeLa cells. The *p*-value was calculated using a two-sided Kolmogorov–Smirnov test. **c**, Two-sided Spearman correlation of relative activity of all tested gnomAD rare variants between HEK293 and HeLa cells. Source data are provided as a Source Data file.

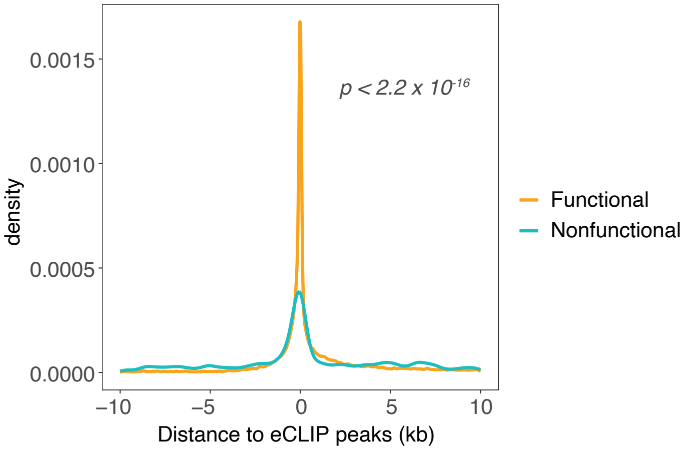

Fig. S7 | Spatial distribution of functional variants around RBP binding sites.
The spatial distribution of functional and nonfunctional variants around RBP binding sites according to ENCODE eCLIP data. P-value was calculated using a two-sided Kolmogorov-Smirnov test. Source data are provided as a Source Data file.

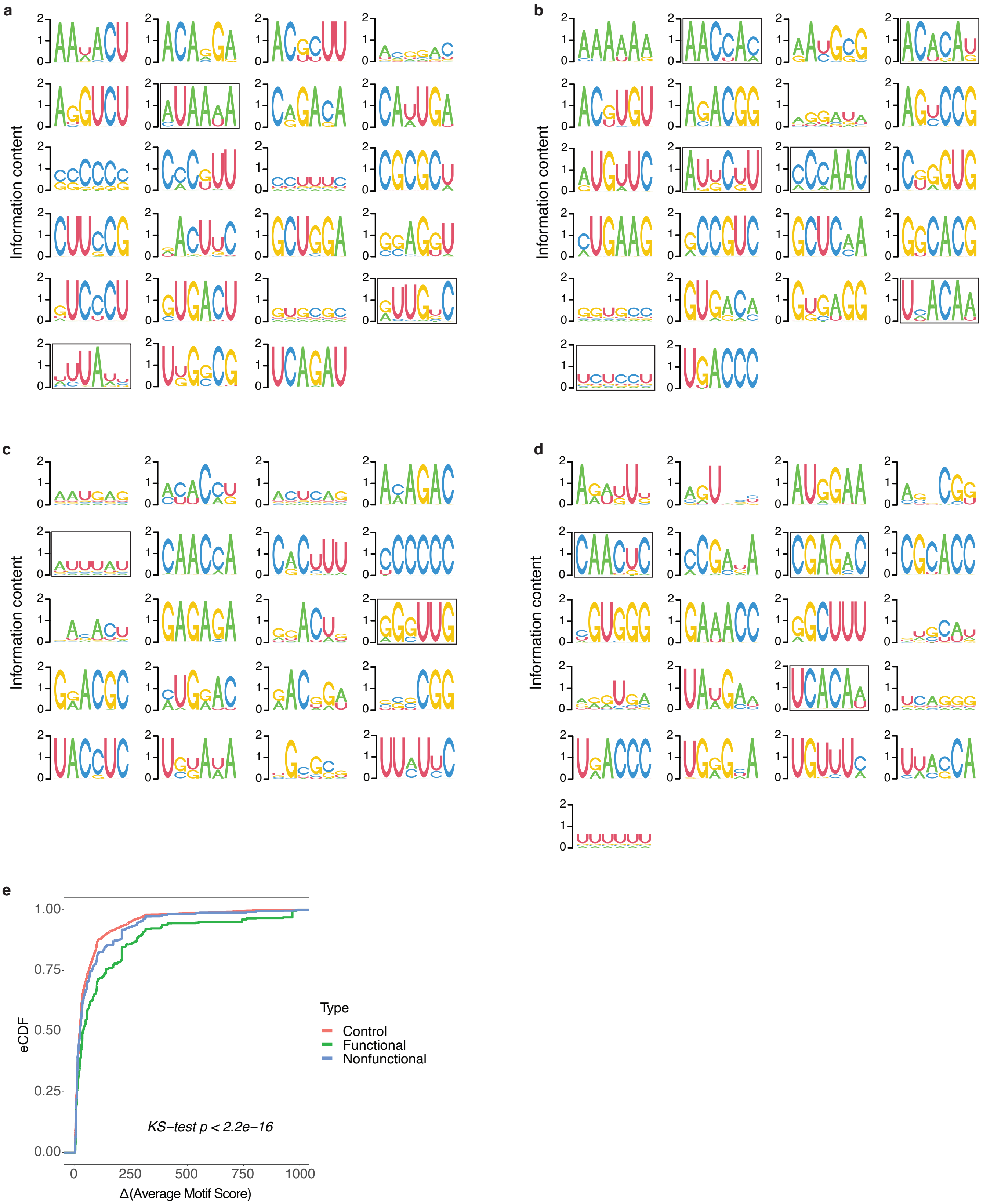

Fig. S8 I Overrepresented motifs in functional variants.
a, Downregulating funtional variants in HEK293; boxes indicate AU- and GU-rich motifs. **b**, Upregulationg functional variants in HEK293; boxes indicate CU- and CA-rich motifs. **c**, Downregulating funtional variants in HeLa; boxes indicate AU- and GU-rich motifs. **d**, Upregulationg functional variants in HeLa; boxes indicate CA- and GA-rich motifs. **e**, Motif strength is significantly altered by functional MapUTR variants compared to nonfunctional ($p < 2.2e\text{-}16$) and shuffled PWM controls ($p < 2.2e\text{-}16$). P-values were calculated using a two-sided Kolmogorov-Smirnov test. Source data are provided as a Source Data file.

a

Motifs (Downregulating)	RBPs
AAUACU	NUPL2
ACGCUU	MBNL1
ACGGAC	A1CF
AUAAAA	A1CF, DAZAP1, HNRNPD, HNRNPDL, NUPL2, RBM47, RBMS3
CCCCCC	PCBP1, PCBP2, PCBP4, TIA1
CCUUUC	PCBP1, PCBP2, PCBP4, PTBP3
CGCGCU	MBNL1, RBM4
AACUUC	PTBP3
GGAGGU	FUS, HNRNPF, HNRNPH2, ILF2, TAF15
GUCCCU	PCBP1, PCBP4
GUGCGC	FUS, HNRNPK, RBM4, TAF15
GUUGUC	CELF1
UUUAUU	A1CF, BOLL, CPEB1, ELAVL4, FUBP1, HNRNPA0, HNRNPC, HNRNPD, HNRNPDL, KHSRP, PTBP3, RBM15B, RBM24, RBM47, RBMS3, RC3H1, TIA1, ZFP36

Motifs (Upregulating)	RBPs
AAAAAA	DAZAP1, HNRNPDL, NUPL2, RBM47
AACCAC	IGF2BP2
AAUGCG	RBM4
ACACAU	IGF2BP1, IGF2BP2, NOVA1, SNRPA
ACGUGU	CELF1
AGGAUA	DAZAP1
AUGUUC	CELF1
AUUCUU	PTBP3, RBM15B, TIA1
CCCAAC	HNRNPK, PCBP1, PCBP2, PCBP4
CGGGUG	HNRNPF, ILF2
GCCGUC	CPEB1, EIF4G2
GGCACG	HNRNPK
GUGAGG	FUS, ILF2, RBM24, TAF15, TARDBP
UCACAA	IGF2BP1, IGF2BP2, NOVA1, SNRPA
UCUCCU	PTBP3

b

Motifs (Downregulating)	RBPs
AAUGAG	A1CF
AUUUAU	BOLL, ELAVL4, FUBP1, HNRNPA0, HNRNPD, HNRNPDL, KHSRP, RBM15B, RBM47, RBMS3, RC3H1, TIA1, ZFP36
UGCGCG	EIF4G2, FUS, HNRNPK, MBNL1, RBM4, SRSF2
CAACCA	IGF2BP1, IGF2BP2
CACUUU	PTBP3, RBM15B, SNRPA
CCCCCC	PCBP1, PCBP2, PCBP4, TIA1
GAGAGA	SRSF2, TARDBP
GGACUG	A1CF, FUS, ILF2, TAF15
GGGUUG	HNRNPF, ILF2
UACCUC	PTBP3
UUAUUC	HNRNPD, PTBP3, RBM15B, RC3H1, TIA1, ZFP36

Motifs (Upregulating)	RBPs
AG CGG	EIF4G2, PCBP4
AUGGAA	TARDBP
CAACUC	IGF2BP1, IGF2BP2
CGCACC	HNRNPK
GGCUUU	RBM24
UGCAU	HNRNPA2B1, RBFOX2
UAUGAA	TARDBP
UCACAA	IGF2BP1, IGF2BP2, NOVA1, SNRPA
UCAGGG	HNRNPF
UGGGCA	HNRNPA2B1, HNRNPF, ILF2, TAF15, TARDBP
UGUUUC	BOLL, CELF1, FUBP1
UUUUUU	BOLL, CELF1, CPEB1, ELAVL4, FUBP1, HNRNPA0 HNRNPC, HNRNPD, HNRNPDL, KSHRP, PTBP3, RBM15B, RBM24, RC3H1, TIA1

Fig. S9 l Motifs bound by RBPs according to RBNS data in (**a**) HEK293 and (**b**) HeLa. In each cell line, the motifs overrepresented in downregulating (left) and upregulating sequences (right) are labeled.

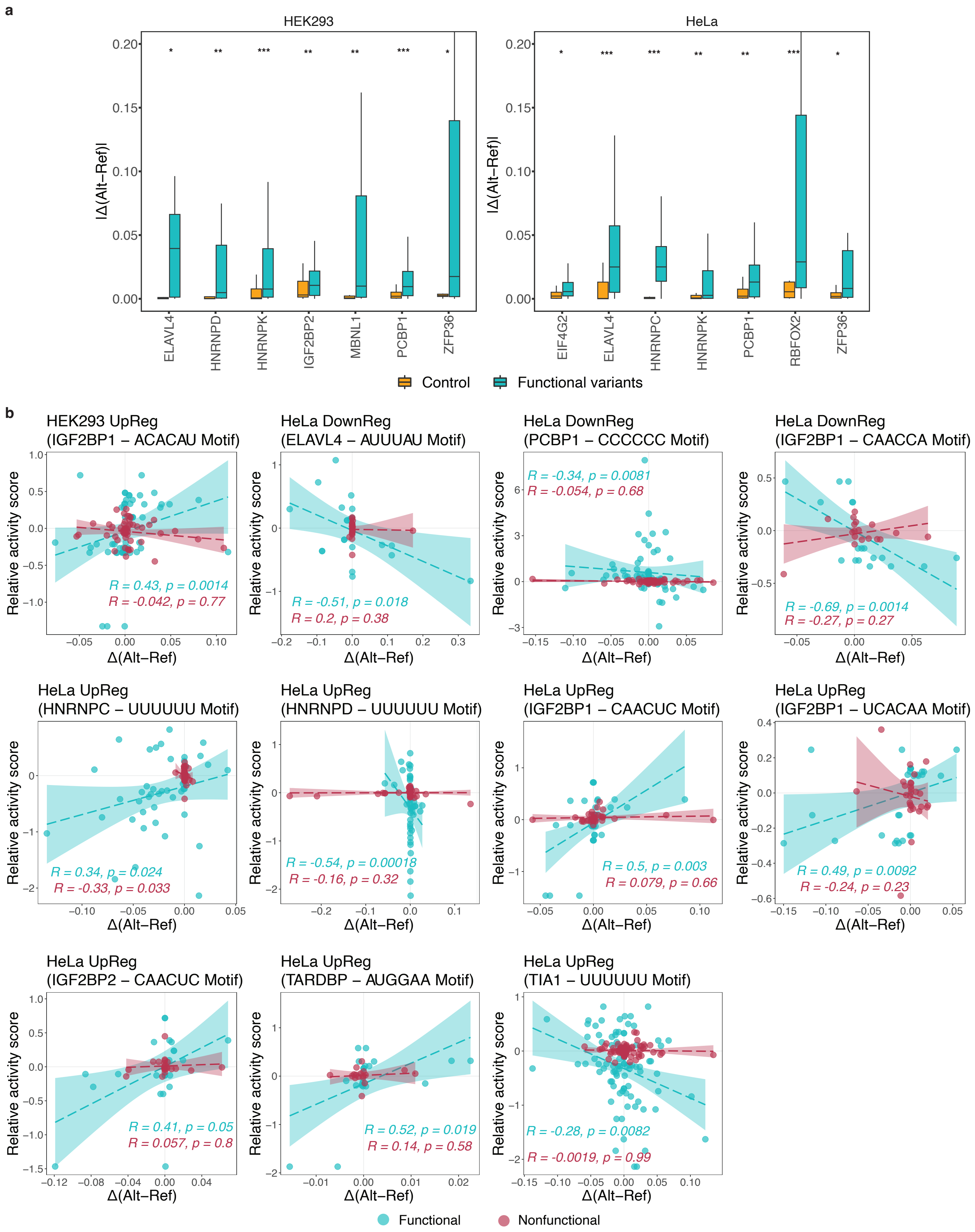

Fig. S10 | Functional impact of rare 3' UTR variants on RBP binding and motif strength.
a, Distibution of DeepRiPe score changes plotted per RBP for functiobal variants in HEK293 and HeLa. P-values were calculated using a two-sided Kolmogorov-Smirnov test. **p*<0.05, ***p*<0.01, ****p*< 0.001. **b**, Relative activity score of functional MapUTR variants tested in HEK293 and HeLa is corroborated by predicted changes in RBP binding to discovered motifs. A two-sided Spearman correlation coefficient was calculated for functional and nonfunctional variants separately. The shaded bands indicate the 95% confidence interval from the line of best fit. All boxplots depict the median as the center line, the boxes define the interquartile range (IQR: 25th to 75th percentiles) and the whiskers extend up to 1.5 times the IQR. Source data are provided as a Source Data file.

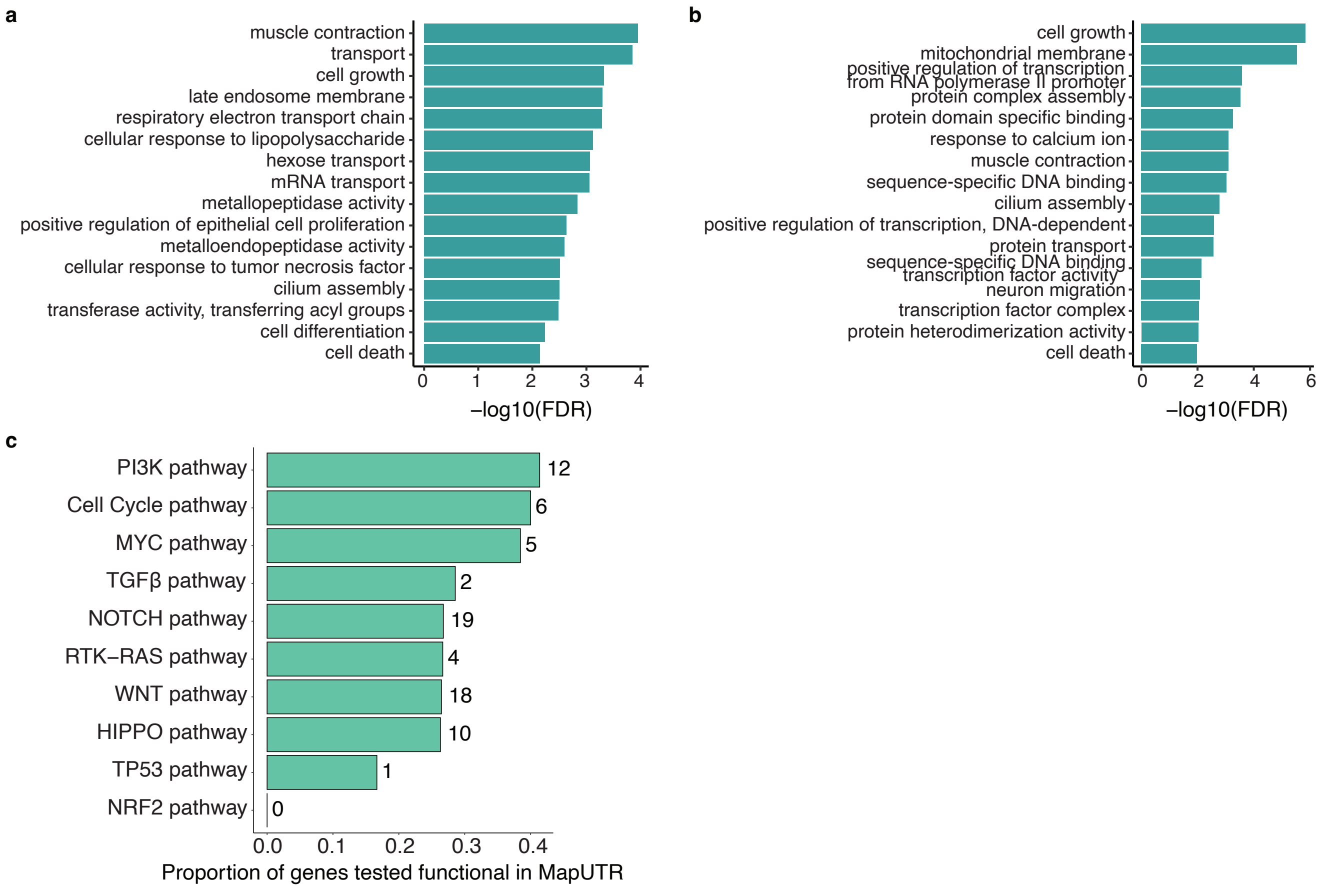

Fig. S11 | Functional relevance of functional rare variants identified by MapUTR.
a, Gene ontology terms enriched in the genes with large-effect (top 500) functional rare variants found in HEK293 cells. **b**, Gene ontology terms enriched in the genes with large-effect (top 500) functional rare variants found in HeLa cells. **c**, Proportion of genes with functional MapUTR variants in 10 oncogenic pathways. The actual number of MapUTR functional genes in the pathway is indicated by the number beside each bar. Source data are provided as a Source Data file.

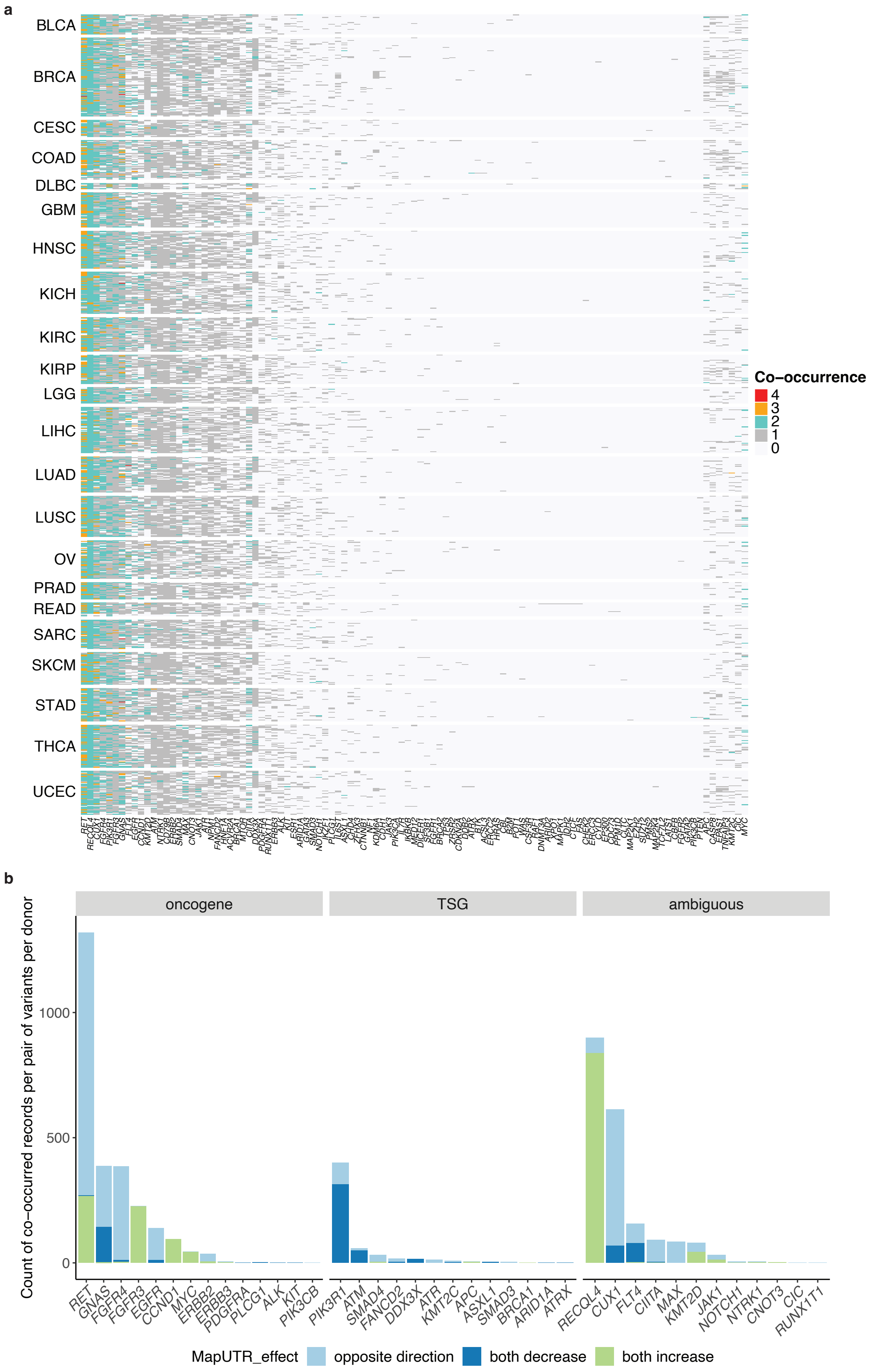

Fig. S12 | Co-occurrence pattern of functional MapUTR cancer somatic mutations in TCGA patients.
a, Heatmap of number of co-occurred variants in the same cancer driver gene (column) in each TCGA patient (row). **b**, Number of times a pair of functional MapUTR cancer somatic mutations were observed in the same gene across all patients. Genes with at least two functional variants co-occurred in one patient are plotted. TSG, tumor suppressor gene. Opposite direction, the co-occurred variants had opposite effect on mRNA abundance (one increase, one decrease). Both decrease, both of the co-occurred variants decreased mRNA abundance. Both increase, both of the co-occurred variants increased mRNA abundance. **a-b**, Source data are provided as a Source Data file.

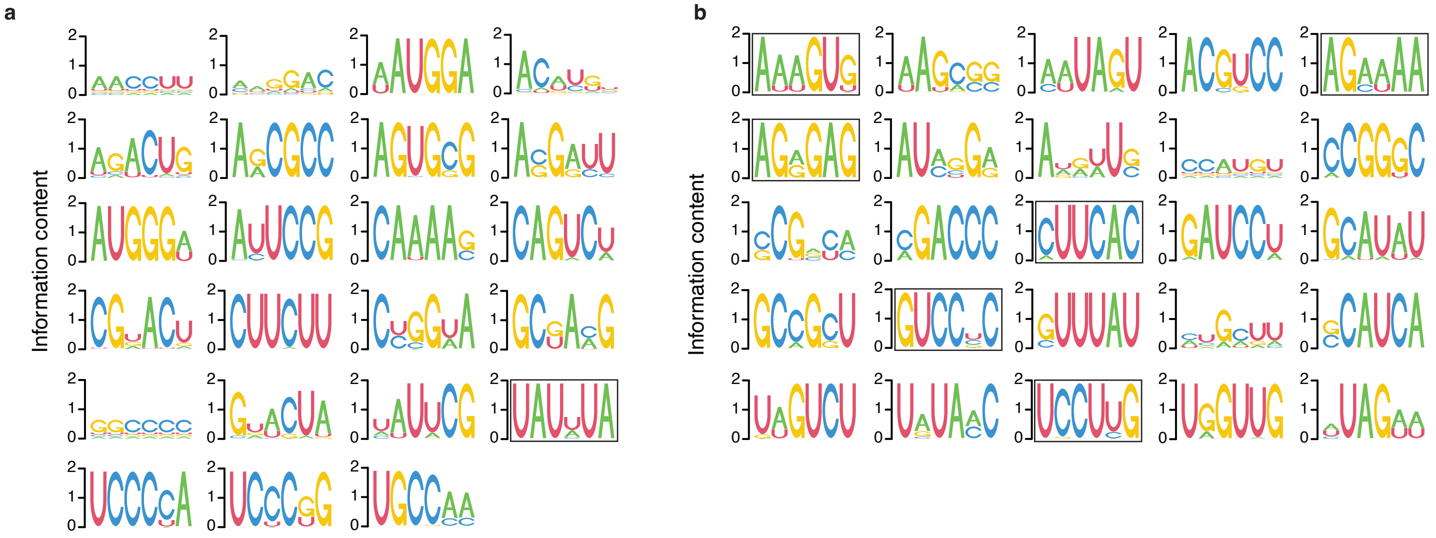

c

Motifs (Downregulating)	RBPs
AAUGGA	NUPL2
ACAUG	CELF1, PUM1
AGUGCG	RBM4, TARDBP
AUGGGA	HNRNPF, ILF2, TARDBP
CAAAAG	NUPL2
CUUCUU	PTBP3, RBM15B
GGCCCC	PCBP1, PCBP2
UAUUA	BOLL, DAZAP1, ELAVL4, FUBP1, HNRNPA0, HNRNPD, HNRNPDL, KHSRP, MSI1, PUM1, RBM15B, RBMS3, RC3H1, TIA1, UNK, ZFP36
UCCCCA	PCBP1, PCBP2, PCBP4

d

Motifs (Upregulating)	RBPs
AAAGUG	NUPL2
AAUAGU	DAZAP1, HNRNPA0, HNRNPD, HNRNPDL, MSI1, UNK
ACGUCC	HNRNPK
AGAAAA	NUPL2
AGAGAG	HNRNPA2B1, HNRNPF, HNRNPH2, ILF2, SRSF2, TARDBP
AUAGGA	DAZAP1, HNRNPA0, MSI1
AUG	CELF1
CCAUGU	CELF1, PUM1
CCGCA	HNRNPK, PCBP1
CGACCC	PCBP1, PCBP2
CUUCAC	IGF2BP1, SNRPA
GCAUAU	HNRNPA2B1, KHSRP, NOVA1, RBMS3, RC3H1
GCCGCU	EIF4G2, MBNL1, SRSF2
GUCCC	HNRNPK, PCBP1, PCBP2, PCBP4
GUUUAU	BOLL, CELF1, ELAVL4, FUBP1, HNRNPA0, KHSRP, PUM1, RBM15B, RC3H1
GCUU	MBNL1, PTBP3
GCAUCA	HNRNPK, IGF2BP1, IGF2BP2, NOVA1
UAGUCU	CELF1, DAZAP1, HNRNPA0, MSI1, UNK
UAUAAC	DAZAP1, HNRNPD, HNRNPDL, RBMS3
UAGAA	DAZAP1, HNRNPA0, HNRNPD, MSI1, UNK

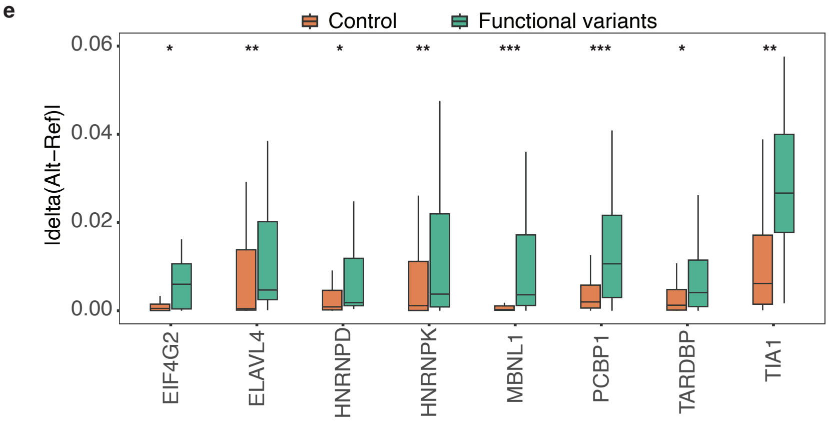

Fig. S13 | Overrepresented motifs in COSMIC functional variants.
a, Downregulating funtional variants in cancer driver genes; AU-rich element is boxed. **b**, Upregulationg functional variants in cancer driver genes; CU-rich and GA-rich motifs are boxed. **c-d**, Downregulating and upregulating motifs, respectively, bound by RBPs according to RBNS data. **e**, Functional variants in 3' UTR of cancer driver genes significantly change RBP binding to mRNA. P-values were calculated using a two-sided Kolmogorov-Smirnov test. *p<0.05, **p<0.01, ***p< 0.001. Source data are provided as a Source Data file.

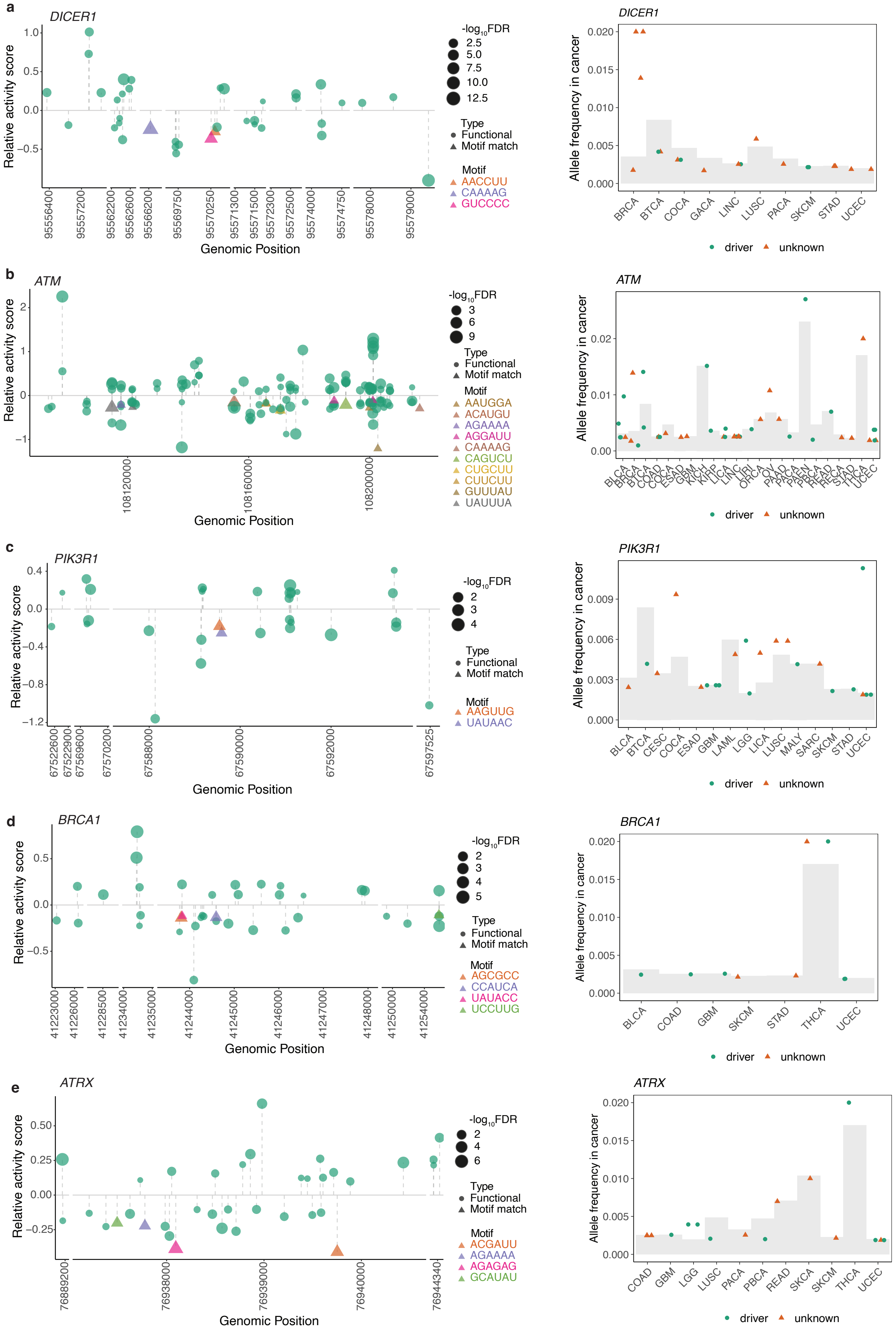
a
DICER1
Relative activity score
Genomic Position
-log10FDR
2.5
5.0
7.5
10.0
12.5
Type
Functional
Motif match
Motif
AACCUU
CAAAAG
GUCCCC
95556400
95557200
95562200
95562600
95566200
95569750
95570250
95571300
95571500
95572300
95572500
95574000
95574750
95578000
95579000
DICER1
Allele frequency in cancer
BRCA
BTCA
COCA
GACA
LINC
LUSC
PACA
SKCM
STAD
UCEC
driver
unknown
b
ATM
Relative activity score
Genomic Position
108120000
108160000
108200000
-log10FDR
3
6
9
Type
Functional
Motif match
Motif
AAUGGA
ACAUGU
AGAAAA
AGGAUU
CAAAAG
CAGUCU
CUGCUU
CUUCUU
GUUUAU
UAUUUA
ATM
Allele frequency in cancer
BLCA
BRCA
BTCA
COAD
COCA
ESAD
GBM
KICH
KIRP
LICA
LINC
LIRI
ORCA
OV
PAAD
PACA
PAEN
PBCA
READ
RECA
STAD
THCA
UCEC
driver
unknown
c
PIK3R1
Relative activity score
Genomic Position
67522600
67522900
67569600
67570200
67588000
67590000
67592000
67597525
-log10FDR
2
3
4
Type
Functional
Motif match
Motif
AAGUUG
UAUAAC
PIK3R1
Allele frequency in cancer
BLCA
BTCA
CESC
COCA
ESAD
GBM
LAML
LGG
LICA
LUSC
MALY
SARC
SKCM
STAD
UCEC
driver
unknown
d
BRCA1
Relative activity score
Genomic Position
41223000
41226000
41228500
41234000
41235000
41244000
41245000
41246000
41247000
41248000
41250000
41254000
-log10FDR
2
3
4
5
Type
Functional
Motif match
Motif
AGCGCC
CCAUCA
UAUACC
UCCUUG
BRCA1
Allele frequency in cancer
BLCA
COAD
GBM
SKCM
STAD
THCA
UCEC
driver
unknown
e
ATRX
Relative activity score
Genomic Position
76889200
76938000
76939000
76940000
76944340
-log10FDR
2
4
6
Type
Functional
Motif match
Motif
ACGAUU
AGAAAA
AGAGAG
GCAUAU
ATRX
Allele frequency in cancer
COAD
GBM
LGG
LUSC
PACA
PBCA
READ
SKCA
SKCM
THCA
UCEC
driver
unknown

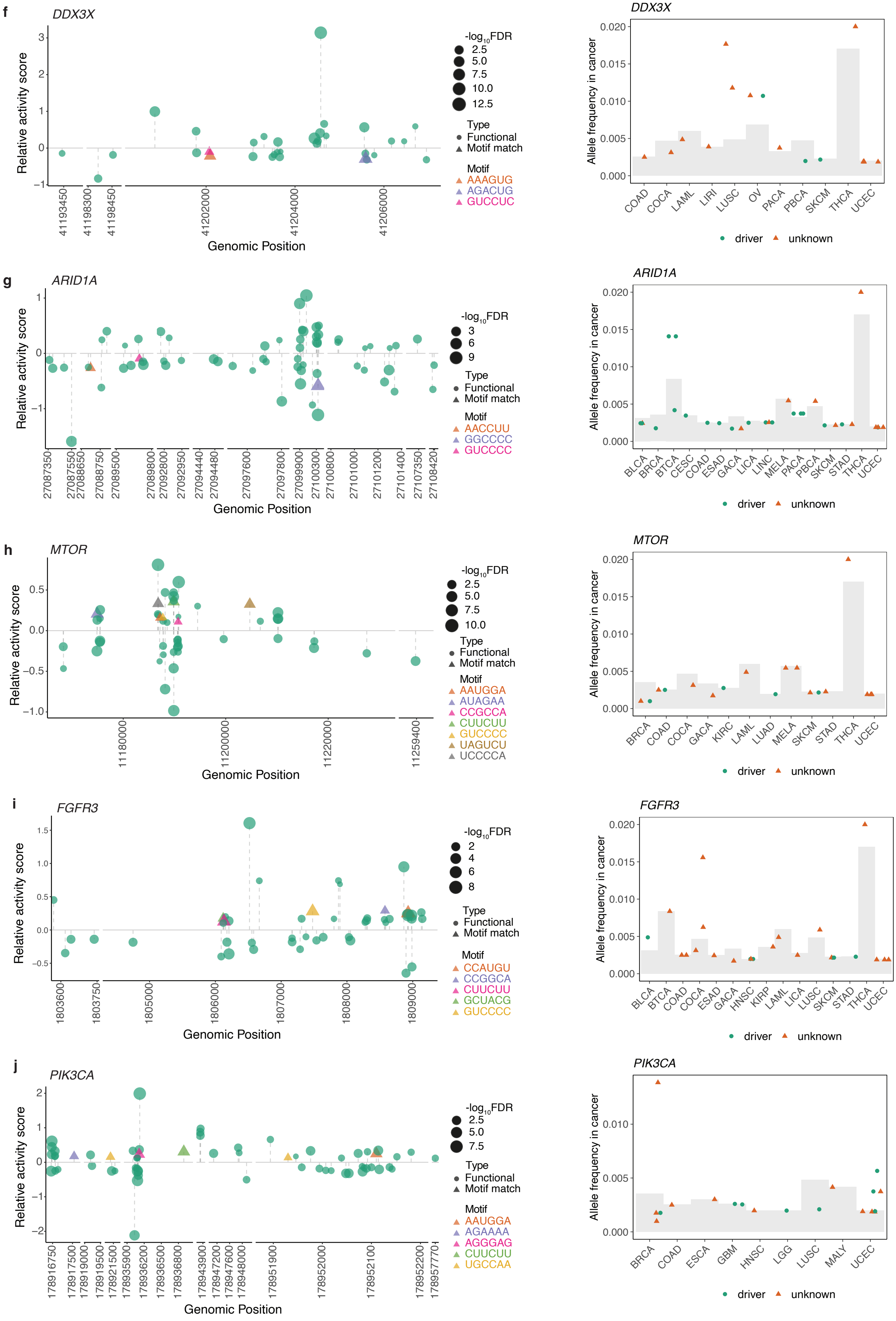

Fig. S14 l Motif analysis for pro-cancer funcitonal variants in cancer driver genes.
a-g, Left: Overlap of functional variants with discovered motifs in tumor suppressor genes (TSGs). Right: Allele frequencies of down-regulating functional variants in TSGs across various cancers. **h-j**, Left: Overlap of functional variants with discovered motifs in oncogenes. Right: Allele frequencies of up-regulating functional variants in oncogenes across various cancers. Source data are provided as a Source Data file.

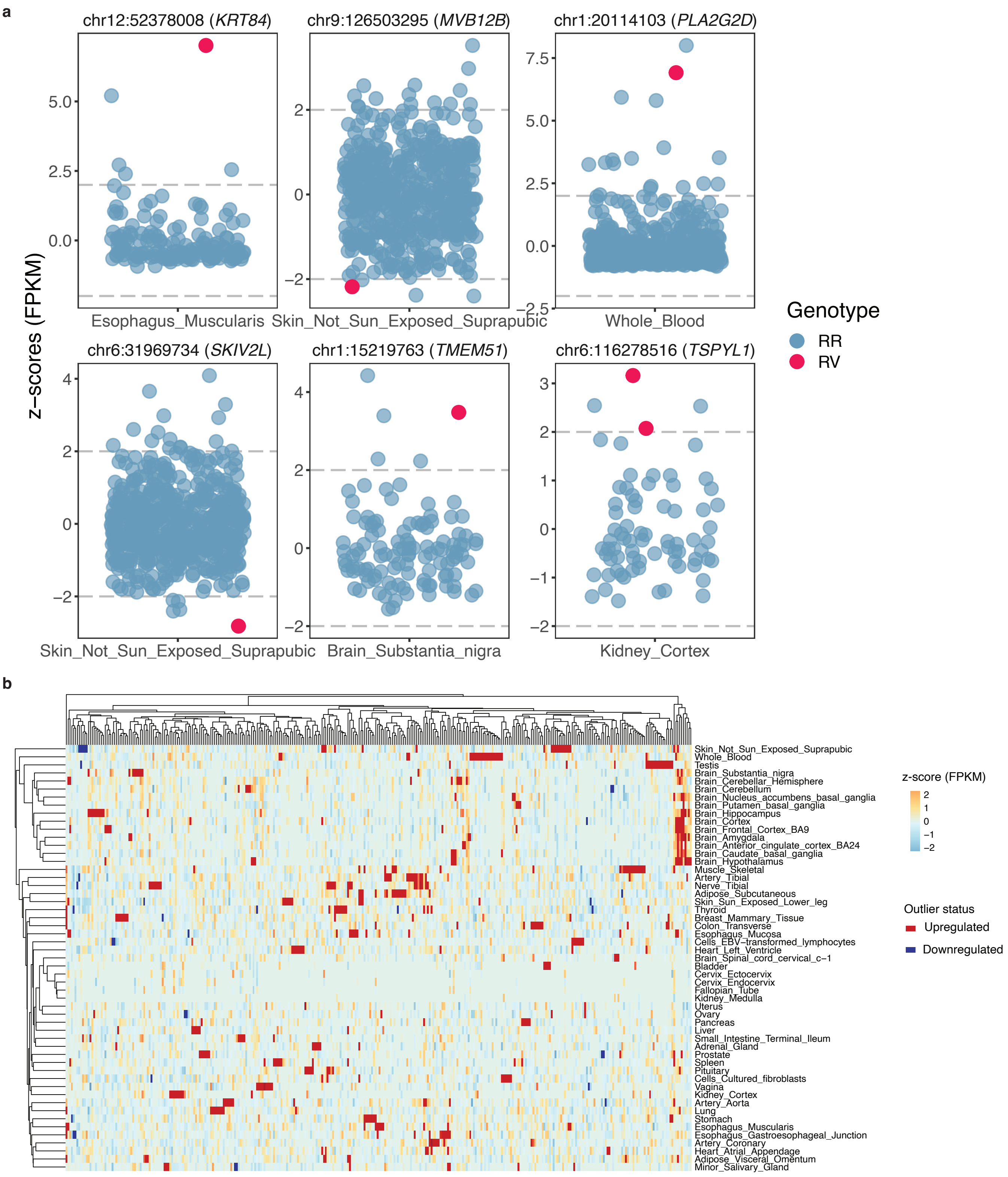

Fig. S15 | Functional MapUTR variants in GTEx tissues.
a, Examples of MapUTR variants with outlier expression in GTEx tissues. **b**, Heatmap of all outlier genes (columns) in all tissues (rows). For each gene, if an outlier was found in at least one sample of the tissue, we plotted the gene as dark red (upregulated) or dark blue (downregulated). Otherwise, we plotted the z-score of the gene's FPKM in samples with functional variants. Source data are provided as a Source Data file.

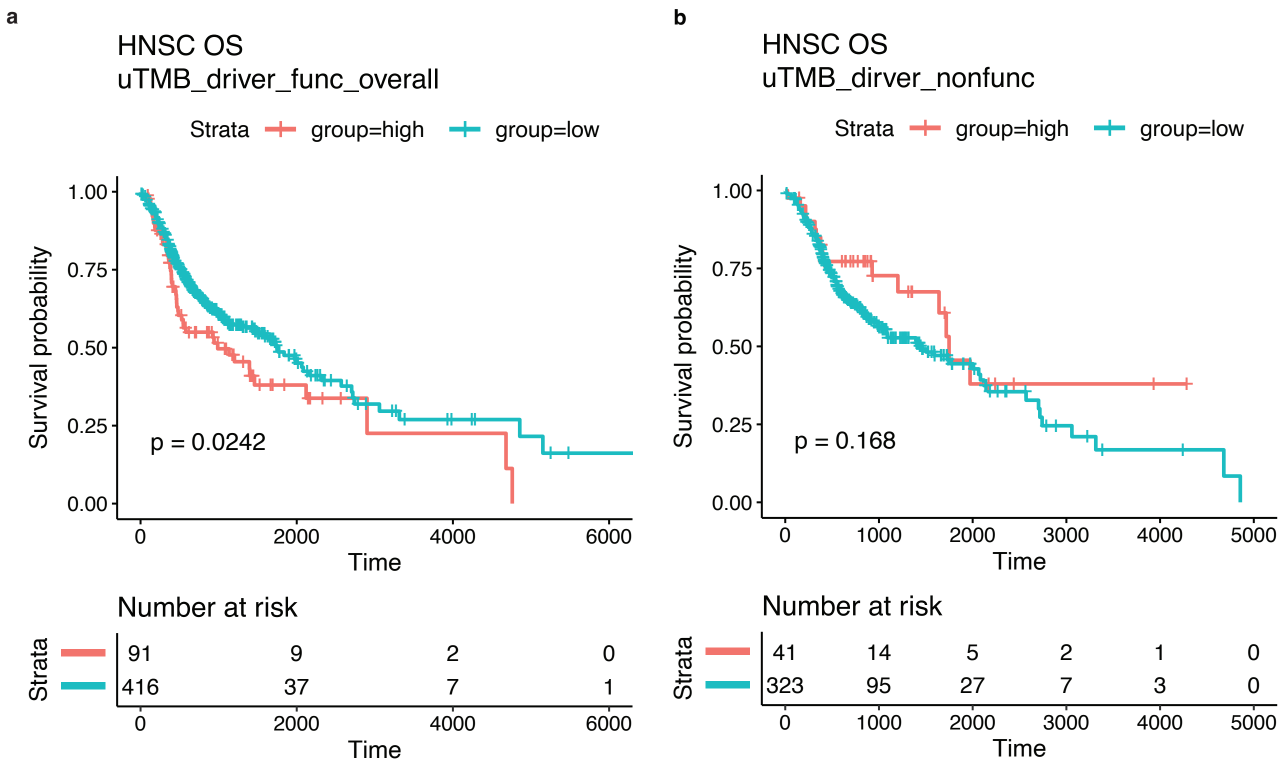

Fig. S16 | Functional MapUTR variants in cancer driver genes are associated with patient survival in HNSC.
a, Higher uTMB of functional MapUTR variants in cancer driver genes (uTMB_driver_func_overall) is associated with worse overall survival in HNSC. Patients were grouped into high (orange) and low (turquoise) groups by uTMB_driver_func_overall level tertiles. The *p*-value was calculated by the log-rank test. **b**, The uTMB of non-functional MapUTR variants in cancer driver genes (uTMB_driver_nonfunc) is not associated with overall survival in HNSC. Patients were grouped into high (orange) and low (turquoise) groups by uTMB_driver_nonfunc level tertiles. The *p*-value was calculated by two-sided log-rank tests. Source data are provided as a Source Data file.

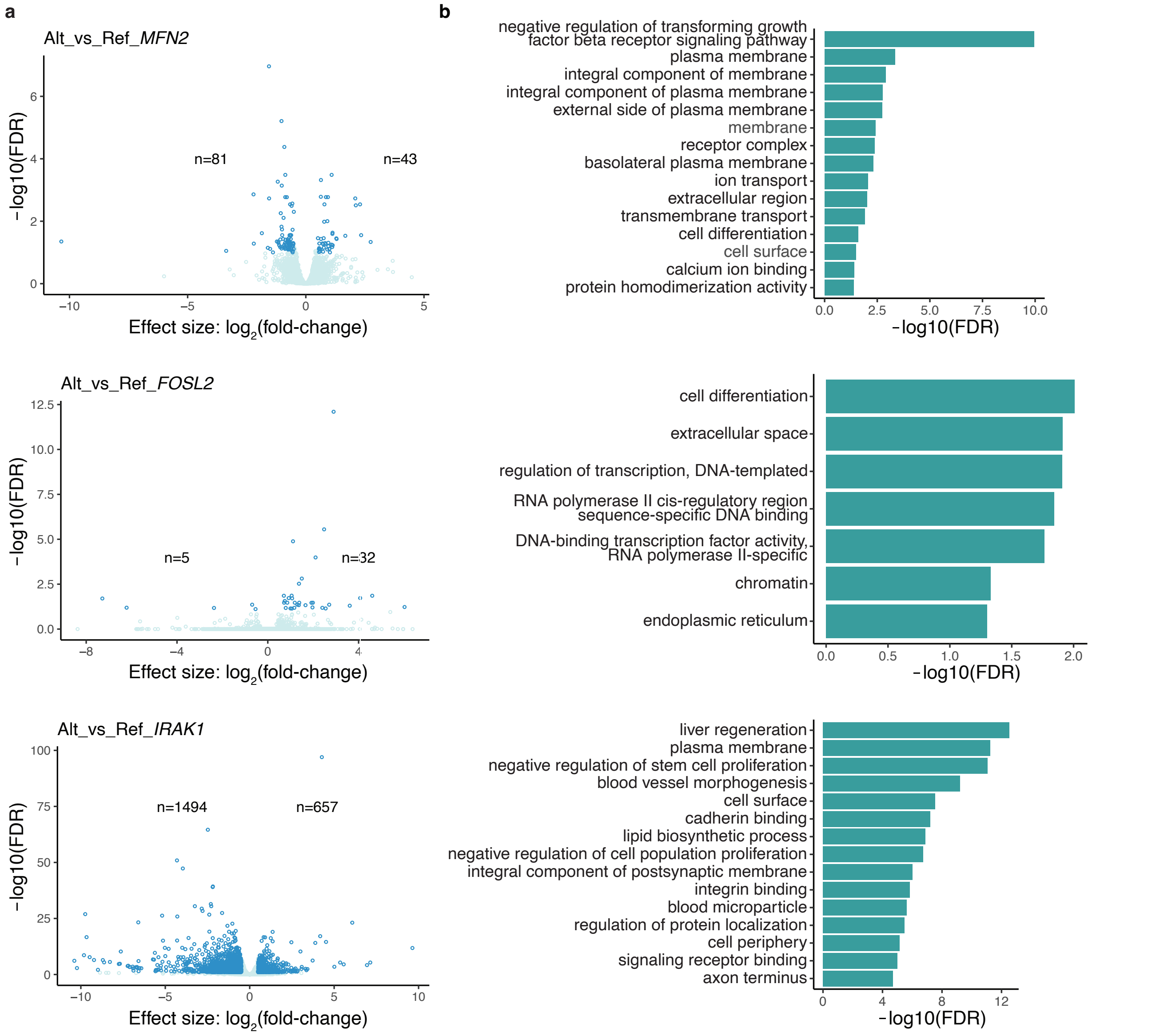

Fig. S17 l Differentially expressed genes between alternative and reference alleles of *MFN2*, *FOSL2*, and *IRAK1*.
a, Volcano plots of differentially expressed (DE) genes identified by DESeq2 between alternative and reference alleles of gene *MFN2* (top), *FOSL2* (middle), and *IRAK1* (bottom). Three single-cell clones (N=3 biologically independent samples) per allele per gene were included for each comparison, except for IRAK1 alternative allele, where two single-cell clones (N=2 biologically independent samples) were included. DE genes were defined as $|\log_2(\text{fold-change})| > 0.5$ and FDR < 0.1. DE genes were highlighted with dark blue circles. **b**, Gene ontology terms enriched in the DE genes defined in **a** for *MFN2* (top), *FOSL2* (middle), and *IRAK1* (bottom). For *IRAK1*, the top 500 genes ranked by FDR instead of all DE genes were used as query genes for analysis (See Methods). Top 15 (or less) GO terms ranked by FDR were plotted. **a-b**, Source data are provided as a Source Data file.

Supplementary Table 1. Well-known 3' UTR motifs tested in Fig. 1f.

Gene	Chrom	Start (hg19)	End	Strand	Motif	Sequence
CXCL2	chr4	74963304	74963325	-	ARE	UAUUUAUUUAUUUAUUU AUUUAU
RBBP5	chr1	205056883	205056898	-	CDE	UCCUUUCUGUGAAAGG
CHRDL1	chrX	109919400	109919417	-	SAMD4A	AAGCUGCAGCUGGACUGC
MYOD1	chr11	17743520	17743529	+	hPUM	UGUAAAUAAG
SIPA1L2	chr1	232534007	232534014	-	dPUM	UGUACAGA

Supplementary Table 2. Filtering Criteria for MPRA Library Design.

Design	Sequence	Filters
Design 1: F+rec1+lib+R	Original sequence	• Only one occurrence of REC1 (EcorRI)<br>• Does not contain REC2 (BamHI)<br>• Only one occurrence of each of the corresponding subpool1 forward and reverse amplification sequences (8 nt in the 3' end of the primer)<br>• Does not contain any pair of other subpools' (subpool 2,3,4) forward and reverse amplification sequences (8 nt in the 3' end of the primer)
	Reverse complement of the original sequence	• Only one occurrence of REC1 (EcorRI)<br>• Does not contain REC2 (BamHI)<br>• Does not contain corresponding subpool1 forward amplification sequence (8 nt in the 3' end of the primer)<br>• Does not contain corresponding subpool1 reverse amplification sequence (8 nt in the 3' end of the primer)<br>• Does not contain any pair of other subpools' (subpool 2,3,4) forward and reverse amplification sequences (8 nt in the 3' end of the primer)
Design 2: F+rec1+lib+rec2+R	Original sequence	• Only one occurrence of REC1 (EcorRI)<br>• Only one occurrence of REC2 (BamHI)<br>• Only one occurrence of each of the corresponding subpool1 forward and reverse amplification sequences (8 nt in the 3' end of the primer)<br>• Does not contain any pair of other subpools' (subpool 2,3,4) forward and reverse amplification sequences (8 nt in the 3' end of the primer)
	Reverse complement of the original sequence	• Only one occurrence of REC1 (EcorRI)<br>• Only one occurrence of REC2 (BamHI)<br>• Does not contain corresponding subpool1 forward amplification sequence (8 nt in the 3' end of the primer)<br>• Does not contain corresponding subpool1 reverse amplification sequence (8 nt in the 3' end of the primer)<br>• Does not contain any pair of other subpools' (subpool 2,3,4) forward and reverse amplification sequences (8 nt in the 3' end of the primer)

Supplementary Table 3. Off-target sites for guide RNAs used in prime editing.

Name	crRNA	DNA	Cluster Position (hg38, 0-based)	Direction	Mismatches	Bulge Size	CFD
MFN2_offtarget1	GGCCATACTTCTTTCAGAAANNN	tGaCAcACTTCTTTCAaAAATGG	chr5:96295408	+	4	0	0.73535354
	G-GCCATACTTCTTTCAGAAANNN	aTGaCAcACTTCTTTCAaAAATGG	chr5:96295408	+	4	1	0.73535354
MFN2_offtarget2	G-GCCATACTTCTTTCAGAAANNN	GAGaCAcACaTCcTTCAGAAAAGG	chr10:95408920	-	4	1	0.69309638
MFN2_offtarget3	GGCCATACTTCTTTCAGAAANNN	t-CaATAtTTCTTTCAGAAATGG	chrX:4717017	+	3	1	0.67927632
FOSL2_offtarget1	TCCCTCCCCAGCTCCGGAGGNNN	aCCCTtCCCAGCTCCaGAGaAGG	chr16:31090772	+	4	0	0.87053571
	T-CCCTCCCCAGCTCCGGAGGNNN	cACCCTtCCCAGCTCCaGAGaAGG	chr16:31090772	+	4	1	0.87053571
FOSL2_offtarget2	TCCCTCCCCAGCTCCGGAGGNNN	aCCCTtCCCAGCTCCaGAGaAGG	chr16:31092500	-	4	0	0.87053571
	T-CCCTCCCCAGCTCCGGAGGNNN	cACCCTtCCCAGCTCCaGAGaAGG	chr16:31092500	-	4	1	0.87053571
FOSL2_offtarget3	TCCCTCCCCAGCTCCGGAGGNNN	g-CCTtCCCAGCTCCGGAGGCGG	chr19:2294399	-	2	1	0.86044255
FOSL2_offtarget4	GGAGCGAGGAGGACTCCCTCNNN	GGAGtGAaGtGGACTaCCTCGGG	chrX:29617781	-	4	0	0.56149733
	G-GAGCGAGGAGGACTCCCTCNNN	GGGAGtGAaGtGGACTaCCTCGGG	chrX:29617781	-	4	1	0.56149733
	GGAGCGAGGAGGACTCCCTCNNN	G-AGtGAaGtGGACTaCCTCGGG	chrX:29617781	-	4	1	0.51763035
FOSL2_offtarget5	GGAGCGAGGAGGACTCCCTCNNN	tG-agGAGGAaGACTCCCTCAGG	chr7:28766739	+	4	1	0.48
FOSL2_offtarget6	GGAGCGAGGAGGACTCCCTCNNN	GG-GCaAGGAacACTaCCTCAGG	chr9:96062501	+	4	1	0.47058824
IRAK1_offtarget1	CTTCTCTCCCCCGCGGGCATNNN	C-TCctTCCCCCGCaGaCATGGG	chr8:143586472	-	4	1	0.77745098
	CTTCTCTCCCCCGCGGGCATNNN	CT-CctTCCCCCGCaGaCATGGG	chr8:143586472	-	4	1	0.75196078
IRAK1_offtarget2	C-TTCTCTCCCCCGCGGGCATNNN	CTTTCTtTCCtCCaCaGGCATTGG	chr11:80157991	+	4	1	0.75926841
IRAK1_offtarget3	CTTCTCTCCCCCGCGGGCATNNN	Cc-CTCTCCaCCaCaGGCATGGG	chr1:158091341	+	4	1	0.59495798
IRAK1_offtarget4	GGG-TGGGGGCTCATGCCCGCNNN	tGGCTGaaGGaTCATGCCCGCTGG	chr8:29664635	-	4	1	0.57777778
	GGGTGGGGGCTCATGCCCGCNNN	Gc-TGaaGGaTCATGCCCGCTGG	chr8:29664635	-	4	1	0.49354839
IRAK1_offtarget5	GGG-TGGGGGCTCATGCCCGCNNN	GtGCTGGaGGCTCATaCCCaCGGG	chr10:77375945	-	4	1	0.44817927
IRAK1_offtarget6	G-GGTGGGGGCTCATGCCCGCNNN	cCGGTGGGGGCTCAaGCCCtCAGG	chr12:132538552	-	3	1	0.41269841

Supplementary Method

Part I. Plasmid library cloning

Materials

Reagents

- UltraPure DNase/RNase-Free Distilled Water (Invitrogen, Cat# 10977015)
- PowerUp™ SYBR® Green Master Mix (Thermo Fisher Scientific, Cat# A25743)
- Q5 High-Fidelity 2x Master Mix (NEB, Cat# M0492L)
- Zymo DNA clean & Concentrator Kit (Zymo Research, Cat# D4004)
- Zymoclean™ Gel DNA Recovery Kit (Zymo Research, Cat# D4002)
- EcoRI-HF (NEB, Cat# R3101S)
- BamHI-HF (NEB, Cat# R3136S)
- T7 DNA Ligase (NEB, Cat# M0318)
- 10-beta Electrocompetent *E. coli* (NEB, Cat# C3020K)
- Electroporation Cuvettes, 0.1cm gap (Bio-Rad, Cat. # 1652089)
- Fisherbrand™ Petri Dishes with Clear Lid, 150mm x 15mm (Fisher Scientific, Cat# FB0875714)
- ZymoPURE II Plasmid Midiprep Kit (Zymo Research, Cat# D4200)

Primers

Name	Sequence
Subpool1_F	GGTCGAGCCGGAACT
subpool2_F	CGATCGCCCTTGGTG
subpool3_F	GGGTCACGCGTAGGA
Subpool4_F	CGCGTCGAGTAGGGT
Subpool1_BamHI_R	TTACGTGGATCCGGATGCGCACCCAGA
subpool2_BamHI_R	TTACGTGGATCCGGTTTAGCCGGCGTG
subpool3_BamHI_R	TTACGTGGATCCGTTCCGCAGCCACAC
Subpool4_BamHI_R	TTACGTGGATCCGCCGTGTGAAGCTGG
polyA_MluI_R	TTTACGCGTTAAGATACATTGATGAG

*All primers listed above were synthesized by IDT with standard desalting purification.

Procedure

1. Resuspend oligo library (Twist Biosciences) in Ultrapure distilled water at a final concentration of 1ng/µl.
2. Assemble qPCR reaction for each subpool (below showing the reaction for chip1.subpool1, same for other subpools)

Reagent	Volume(µl)
PowerUp SYBR 2x master mix	25
Subpool1_F (10µM)	2.5
Subpool1_BamHI_R (10µM)	2.5

Resuspended oligos	1
dH_2O	19

Load 20µl of the mixed reaction to the qPCR 96-well plate (2 wells per subpool)

Run qPCR as follows:

UDG activation	50 °C	10min
Initial denaturation	95 °C	2min
45 cycles	95 °C	15s
	60 °C	30s

3. Repeat PCR by replacing the SYBR master mix with the Q5 high-fidelity 2x master mix, set up three 50µl reactions for each subpool, use the cycle number where the slope begins to decrease in the qPCR pre-run (usually 17-19 cycles).

Reagent	Volume(µl)
Q5 2x master mix	75
Subpool1_F (10µM)	7.5
Subpool1_BamHI_R (10µM)	7.5
Resuspended oligos	3
dH_2O	57

Distribute the mixed reaction to 3 PCR tubes (50µl each).

Run PCR as follows:

Initial denaturation	98 °C	30s
17-19 cycles	98 °C	10s
	60 °C	30s
	72 °C	30s
Final extension	72 °C	2min

4. Run 20µl of PCR products on a 2% agarose gel to check the band size; save the rest PCR products for direct PCR clean up using the Zymo DNA clean & concentrator kit, elute with distilled water.
5. Quantitate DNA concentration of purified PCR products with BioDrop Fluorometer.
6. Digest 2µg of the master plasmids and 100ng of the purified PCR products with EcoRI-HF and BamHI-HF overnight at 37 °C.
7. Heat inactivation of restriction enzymes at 65 °C for 20min.
8. Load the digested master plasmid reaction on a 1% agarose gel and gel purify the band at 5.7kb.
9. Clean up the digested PCR products directly with the Zymo DNA clean & concentrator kit.
10. Set up ligation with freshly cut vector and inserts at 1:10 molar ratio.

Reagent	Amount
2x T7 ligase buffer	20µl
Digested master plasmid	100ng
Digested inserts	36.6ng
T7 DNA ligase	2µl
dH_2O	to 40µl

*Set up one ligation reaction per subpool; set up a ligation reaction without inserts (inserts replaced with water) for background calculation.
Incubate the reaction at 25°C for 1hour and then keep on ice.

11. Clean up the ligation reaction using the Zymo DNA clean & concentrator kit, elute with 16µl distilled water.
12. Mix 1µl purified ligation products with 25µl 10-beta electrocompetent *E. coli*. Transfer the mixture to a prechilled 0.1cm electroporation cuvette, and perform electroporation following the manufactory's protocol. After electroporation, immediately add 750µl **pre-warmed** 10-beta outgrowth medium into the cuvette and transfer the mixture to a 1.5ml microcentrifuge tube.
13. Recover transformed *E. coli* at 37 °C for an hour.
14. Make a serial dilution of the transformed *E. coli* (1:1, 1:10, 1:100, 1:1000). Plate 250µl transformed *E. coli* per 150mm Kanamycin-selective plate. For the no-insert ligation control, perform a 1-to-50 dilution before plating.
15. Grow plates at 37 °C overnight
16. Count colonies on the serial dilution plates. The number of colonies represents the plasmid complexity per subpool library. For a subpool with 2000 variants, to ensure 100x coverage, harvest 0.2M colonies (harvest 0.4M colonies to account for the loss during plasmid isolation). One 25µl transformation typically yields colonies ranging from 0.4M~4.5M. Set up multiple electroporation reactions to make sure to get enough colonies for a given subpool.
17. Add 5ml LB media to the selective plate and gently scrape off the colonies. Combine all the colonies for a subpool in a 50ml tube. Mix well.
18. Measure the OD600 of the colony suspension. Make 1-to-10 or 1-to-100 dilutions as necessary for accurate measurement.
19. The plasmid library can be directly extracted from the harvested colonies. Pellet 5ml colony suspension (OD>10) for a library with 0.2M colonies; scale the amount of colony suspension according to the library coverage. Extract plasmids from the pellet using the ZymoPURE II Plasmid Midiprep Kit, same day preferred.
20. (optional) Alternatively, for a subpool with 0.2M colonies, seed 22M *E. coli* (1 OD= 80M *E. coli*/ml) in 50ml LB media (Kan-selective). Grow the culture overnight at 37 °C and extract plasmids using the ZymoPURE II Plasmid Midiprep Kit.
21. Send the plasmid library to Sanger sequencing using the "polyA_MluI_R" primer.

Part II. Cell Electroporation and isolation of mRNA

Materials

Reagents

- Electroporation Cuvettes, 0.4cm gap (Bio-Rad, Cat# 1652086)
- OptiMEM (Gibco, Cat# 31985062)
- Growth Media
  - DMEM (Gibco, Cat# 11995065)
  - 10% FBS (Gibco, Cat# 26140079)

    - Antibiotic-Antimycotic reagent (Gibco, Cat# 15240062)
- Trypsin-EDTA (Gibco, Cat# 25300120)
- TRIzol (Thermo Fisher Scientific, Cat# 15596026)
- Direct-zol RNA Miniprep Plus kit (Zymo Research, Cat# R2072)
- Dynabeads™ Oligo(dT)$_{25}$ (Thermo Fisher Scientific, Cat# 61005)
- Qubit RNA HS Assay Kit (Thermo Fisher Scientific, Cat# Q32852)

Procedure

*The following numbers are designed for the subpool with 2000 variants (ref+alt) and 100x coverage (i.e., 0.2M colonies, see table below for scale up recommendations)

1. On day 0, seed >45M HEK293 (or HeLa) cells in 150mm dishes and make sure they are less than 80% confluent (should be actively dividing cells) by the time of electroporation.
2. One day 1, trypsinize the HEK293 (or HeLa) cells, resuspend with growth media, and count cell numbers.
3. Spin down the cells and resuspend with ice-cold OptiMEM at a cell density of 10M/ml.
4. For each electroporation, mix 750µl (7.5M) cells with 1.5µg plasmid libraries in a pre-chilled microcentrifuge tube, transfer the mixture to a pre-chilled 0.4cm electroporation cuvette, perform electroporation (HEK293: square wave, 25msec, 220V, 0.4cm, HeLa: exponential decay, 500µF, 320V, 0.4cm).
5. Immediately add 1ml warm growth media to the cuvette and transfer the cells to a 150mm petri dish.
6. Combine 2 x 7.5M cell transformants in one 150mm petri dish for one replicate. Perform three replicates for each subpool.
7. Incubate cells at 37 °C for 24h.
8. After 24h, wash the cells in one 150mm petri dish with 10ml pre-warmed PBS, then add 5ml TRIzol to each plate. Lyse the cells at RT for 10min, then transfer the mixture to 1.5ml microcentrifuge tubes. Distribute 500µl lysed mixture per tube. Add 100µl chloroform to each tube and then mix well. Incubate at RT for 5min. Centrifuge at >13,000 g, 4 °C for 15min. Carefully transfer the aqueous upper phase into a new 1.5ml tube. Add equal volume of 100% ethanol and mix well. Load the mixture to six columns supplied by Direct-zol RNA Miniprep Plus kit (Zymo Research, Cat# R2072) to isolate total RNA following the manufacturer's protocol.
9. Isolate the mRNA using the Dynabeads™ Oligo(dT)$_{25}$ (Thermo Fisher Scientific, Cat# 61002). Use 400µg total RNA for one replicate.
    a. Distribute 200µl beads to one 1.5ml microcentrifuge tube, scale up tube numbers based on the amount of total RNA and selection rounds needed. (200µl beads can process 100µg total RNA at each round)
    b. Wash the beads with 1ml binding buffer (20mM Tris-HCl, pH 7.5, 1.0M LiCl and 2mM EDTA) in each tube for two times.
    c. Resuspend beads with 200µl binding buffer.
    d. Dilute 100µg total RNA into 200µl total volume with water, heat at 65 °C for 5min, put on ice.
    e. Binding step: add 200µl diluted total RNA (100µg) into the 200µl beads, mix, incubate at RT for 5min, resuspend, then incubate another 5min.
    f. Wash step: wash the magnetic beads with 1ml wash buffer B (10mM Tris-HCl PH 7.5, 0.15M LiCl, 1mM EDTA) for two times.

g. Elute step: add 15µl Tris buffer to elute mRNA. Elute by heating the beads at 80 °C for 2min. Quickly transfer the elute to a new tube and keep on ice.
h. Next round selection: add 200µl binding buffer to the beads and keep at RT, then repeat steps d-g. Perform 2-3 rounds of selection until all total RNA are processed.
i. Mix all samples in one tube and check concentration with Qubit Fluorometer. Store the mRNA at -80 °C.

Table. DNA and cells used during electroporation for plasmid libraries with different complexity

Variants	Coverage	Colonies	DNA/replicate	Cells/replicate
2000	100x	0.2M	3µg	15M
6000	100x	0.6M	6µg	30M

Part III. Generation of UMI measurement libraries

Materials

Reagents

- SuperScript™ IV First-Strand Synthesis System (Thermo Fisher Scientific, Cat# 18091050)
- Q5 High-Fidelity 2x Master Mix (NEB, Cat# M0492L)
- UltraPure DNase/RNase-Free Distilled Water (Invitrogen, Cat# 10977015)
- PowerUp™ SYBR® Green Master Mix (Thermo Fisher Scientific, Cat# A25743)
- Zymo DNA clean & Concentrator Kit (Zymo Research, Cat# D4004)
- Zymoclean™ Gel DNA Recovery Kit (Zymo Research, Cat# D4002)

Primers

Name	Sequence
MPP3	GTGATTGGAGTTCAGACGTGTGTTCTGCTGACGNNNNNNNNNNNNNNNCGCTCTTCCGATCTGGATCC
MPP2_352	CAAGCAGAAGACGGCATACGAGATTTGGACTTCACCTTAGGCATGGACGAGCTGTACAAATAAGAATTC
MPP2_361	CAAGCAGAAGACGGCATACGAGATCCTCGGTACACCTTAGGCATGGACGAGCTGTACAAATAAGAATTC
MPP2_362	CAAGCAGAAGACGGCATACGAGATAGACTTGGCACCTTAGGCATGGACGAGCTGTACAAATAAGAATTC
MPP2_363	CAAGCAGAAGACGGCATACGAGATATGAGGCTCACCTTAGGCATGGACGAGCTGTACAAATAAGAATTC
MPP2_364	CAAGCAGAAGACGGCATACGAGATGCAGAATCCACCTTAGGCATGGACGAGCTGTACAAATAAGAATTC
MPP2_365	CAAGCAGAAGACGGCATACGAGATGTTGTCCGCACCTTAGGCATGGACGAGCTGTACAAATAAGAATTC
MPP2_366	CAAGCAGAAGACGGCATACGAGATCATGCCATCACCTTAGGCATGGACGAGCTGTACAAATAAGAATTC
MPP2_367	CAAGCAGAAGACGGCATACGAGATTCTCATTCCACCTTAGGCATGGACGAGCTGTACAAATAAGAATTC
MPP2_368	CAAGCAGAAGACGGCATACGAGATGCGCCTGTCACCTTAGGCATGGACGAGCTGTACAAATAAGAATTC
MPP4	AATGATACGGCGACCACCGAGATCTACACTACTCATAGTGATTGGAGTTCAGACGTGTGTTCTGCTGAC*G
MPP1	AATGATACGGCGACCACCGAGATCTACACTATGAGTATTTCCCTACACGACGCTCTTCCG
MPP2	CAAGCAGAAGACGGCATACGAGATTTGGACTTCACCTTAGGCATGGACGAGCTGTAC
UMI.R1.seq	GTGATTGGAGTTCAGACGTGTGTTCTGCTGACG
Read2.seq	CACCTTAGGCATGGACGAGCTGTACAAATAAGAATTC
Index7.seq	GAATTCTTATTTGTACAGCTCGTCCATGCCTAAGGTG

*All primers listed above were synthesized by IDT with PAGE purification, red highlighted sequences are the indexes for sample pooling purposes.

Procedure

a. UMI addition for mRNA

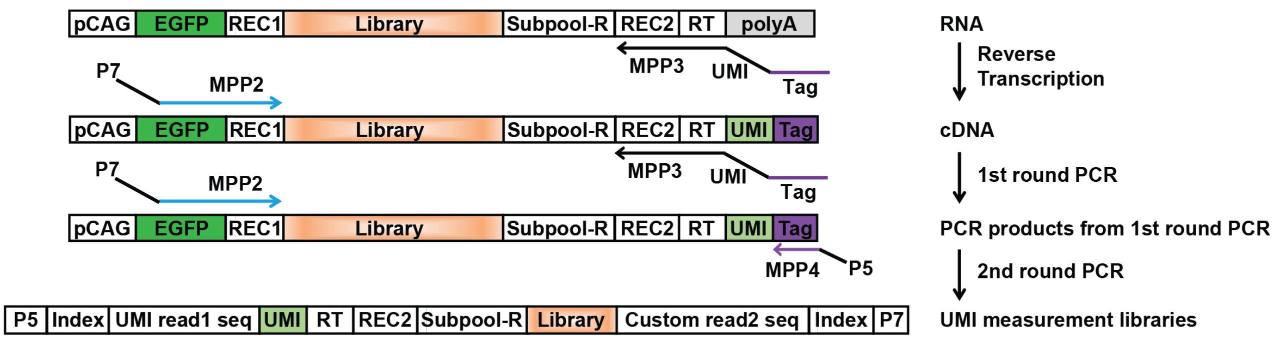

1. Anneal RT primers to the template mRNA.

Reagent	Amount
MPP3 (2µM)	1µl
dNTP mix (10mM)	1µl
mRNA	1µg
Nuclease-free water	To 13µl

   a. Heat on a thermal cycler at 65 °C for 5min.
   b. Promptly remove the samples and put them on ice for 2min.

   *Set-up one no-RT control
   **Use all mRNA(~7µg) isolated from 400µg total RNA for a subpool with 0.2M colonies. Determine the mRNA input by a trial run (e.g., 1 RT reaction) of this protocol with input standards to estimate the complexity of the libraries. The complexity of the libraries should match the downstream sequencing read coverage. (e.g., 20M complexity for 20M reads) Do multiple RT reactions when making the real libraries.

2. Prepare RT reaction mix.

Reagent	Volume(µl)
5x SSIV Buffer	4
DTT (100mM)	1
RNase Inhibitor	1
SuperScriptIV RT	1

   Mix and do a short spin down.

3. Combine RT reaction mix with the annealed RNA by pipetting up and down.
4. Incubation reactions:

RT incubation	50 °C	30min
Inactivation	80 °C	10min

5. Add 1µl RNase H to the 20µl RT reaction and incubate at 37 °C for 20min.

6. Add the UMI with 3-cycle of PCR (first-round)

Reagent	Volume(µl)
Q5 2x master mix	20
MPP2_352 (10µM)	2
MPP3 (10µM)	2
cDNA	10
dH_2O	6

*Use MPP2 primers with different indexes for different samples
**Assemble more PCR reactions as needed to use all the cDNA samples when making the libraries.

Run PCR as follows:

Initial denaturation	98 °C	1min
3 cycles	98 °C	15s
	50 °C	30s
	72 °C	1min
Final extension	72 °C	10min

7. Pool the PCR reactions with the same index in step 6. Directly purify the PCR reactions with the Zymo DNA Clean & Concentrator Kit. Use multiple columns as needed. Elute the samples with distilled water (5µl for one RT reaction).

b. UMI addition for plasmid DNA

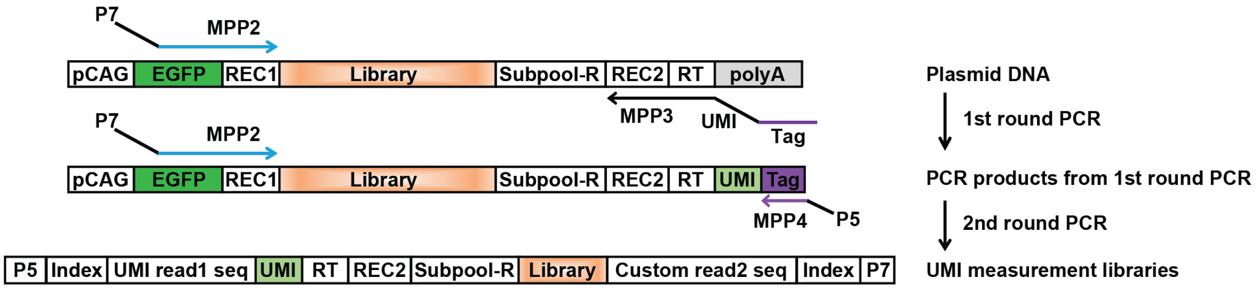

8. Add the UMI to the plasmid DNA using Q5 polymerase (first-round)

Reagent	Amount
2x Q5 master mix	30µl
MPP2_366 (10µM)	3µl
MPP3 (10µM)	3µl
DNA	1200ng
dH_2O	to 60µl

*Use 1200ng DNA for a subpool with 0.2-0.8M colonies. Set up one 60µl reaction per replicate, aliquot in two tubes.

**Determine the DNA input by a trial run of this protocol with standards to estimate the complexity of the libraries. The complexity of the libraries should match the downstream sequencing read coverage. (e.g., 20M complexity for 20M reads)

Run PCR as follows:

Initial denaturation	98 °C	1min
3 cycles	98 °C	15s
	50 °C	30s
	72 °C	1min
Final extension	72 °C	10min

9. Pool the PCR reactions with the same index in step 8. Directly purify the PCR reactions with the Zymo DNA Clean & Concentrator Kit. Elute the samples with 32µl distilled water.

c. Second round library amplification and complexity estimation for mRNA or plasmid DNA

10. Assemble qPCR reactions using part of the eluents from step 7 or step 9 to find the maximum PCR cycles numbers:

Reagent	Volume(µl)
PowerUp SYBR master mix	10
MPP2_X# (10µM)	1
MPP4 (10µM)	1
Eluent from step7	4
dH_2O	4

Load 20µl of the mixed reaction to the qPCR 96-well plate.

#MPP2_X represents the same MPP2 indexed primer used in step 6 or step 8.

Run qPCR as follows:

UDG activation	50 °C	10min
Initial denaturation	95 °C	2min
25 cycles	95 °C	15s
	60 °C	30s

For each sample, check the amplification curve to determine the cycle number before the plateau. Use this cycle number as a cap for the second-round PCR cycles.

11. Set up PCR reaction with DNA standards for library complexity calculation. Make dilutions of the plasmid library to generate DNA standards with the following concentrations: 0.1ng/µl, 0.05ng/µl, 0.025ng/µl, 0.02ng/µl, 0.015ng/µl, 0.01ng/µl, 0.0075ng/µl.

Reagent	Volume(µl)
Q5 2x master mix	10
MPP1 (10µM)	1
MPP2 (10µM)	1

DNA standards	4
dH_2O	4

12. Assemble the second-round PCR run to generate the UMI measurement libraries.

Reagent	Volume(µl)
Q5 2x master mix	10
MPP2_X (10µM)	1
MPP4 (10µM)	1
Eluent from step 6 or 8	4
dH_2O	4

Set up multiple reactions for the same sample until all the eluents from step 7 or 9 are used.

For both library amplification reactions and the standard reactions from step 11, run PCR as follows:

Initial denaturation	98 °C	30s
7-11 cycles	98 °C	10s
	60 °C	30s
	72 °C	30s
Final extension	72 °C	2min

*PCR cycles should be determined by running different PCR cycles with the real library material and find the lowest cycles with visible library band on the agarose gel. PCR cycle numbers should be less than the cycle number determined in step 10. For a subpool of 0.2M colonies, the optimized second-round PCR cycle numbers are 11 cycles for mRNA (step 7) and 8 cycles for plasmid DNA (step 9). For a subpool of 0.6M colonies, the PCR cycles for DNA can be lowered to 7 cycles.

13. Mix 20µl PCR reactions of libraries or standards with 4µl 6x loading dye. Run 20µl of each mixture on a 2% agarose gel. Estimate the library complexity by comparing the band intensity of the libraries (377bp) with the band intensity of the amplicon (342bp) generated from the DNA standards. Use ImageJ for band intensity quantification.
14. Pool the remaining second-round PCR reactions of UMI measurement libraries for the same sample (same index). Purify the reactions with the Zymo DNA Clean & Concentrator Kit. Elute with 20ul distilled water. Resolve the eluents on a 2% agarose gel. Gel purify the band of libraries (377bp) with the Zymoclean™ Gel DNA Recovery Kit. Elute the UMI measurement libraries with 20µl distilled water.
15. Quantitate the libraries with Qubit Fluorometer
16. Mix UMI measurement libraries (generated from both mRNA and plasmid DNA) equally and sequence on Hiseq3000 PE150 or Novaseq SP PE150 with 15% PhiX spike-in and custom sequencing primers (UMI.R1.seq, Read2.seq, and Index7.seq).

Supplementary Note 1. Off-target results for prime editing experiments

Sequence: MFN2_offtarget1_chr5_96295408.pirnt.ver.dna (Linear / 59 bp)
Features: 1 visible, 1 total

10 20 30 40 50

5′ GACTTTATGCCCTGGGCATGACACACTTCTTTCAAAAATGGGATTTGATAATGACATTG 3′

3′ CTGAAATACGGGACCCGTACTGTGTGAAGAAAGTTTTTACCCTAAACTATTACTGTAAC 5′

off-target

GACTTTATGCCCTGGGCATGACACACTTCTTTCAAAAATGGGATTTGATAATGACATTG

1 GACTTTATGCCCTGGGCATGACACACTTCTTTCAAAAATGGGATTTGGTAATGACATTG

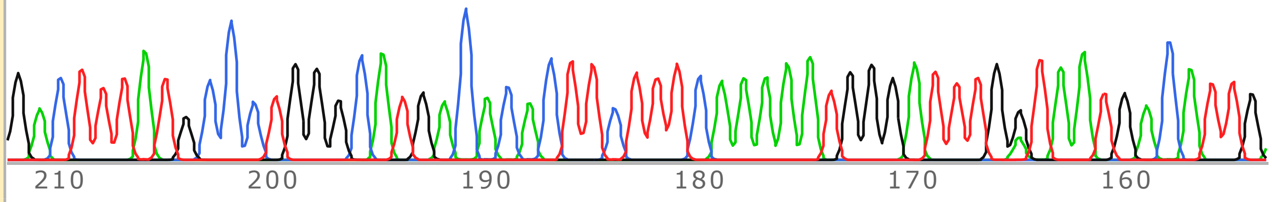

2 GACTTTATGCCCTGGGCATGACACACTTCTTTCAAAAATGGGATTTGATAATGACATTG

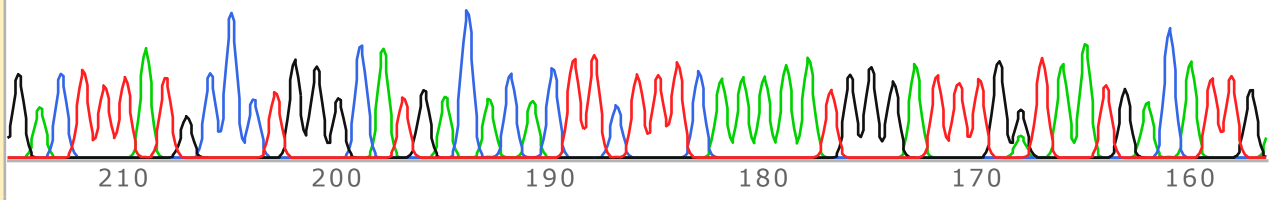

3 GACTTTATGCCCTGGGCATGACACACTTCTTTCAAAAATGGGATTTGGTAATGACATTG

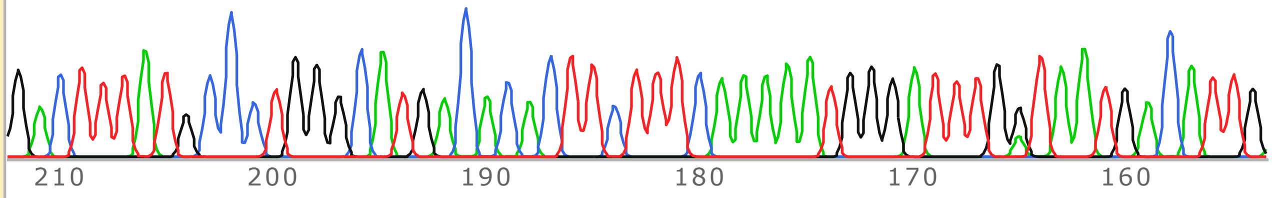

4 GACTTTATGCCCTGGGCATGACACACTTCTTTCAAAAATGGGATTTGATAATGACATTG

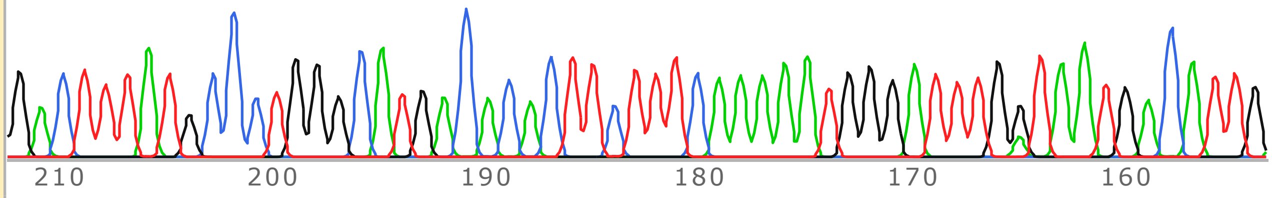

5 GACTTTATGCCCTGGGCATGACACACTTCTTTCAAAAATGGGATTTGATAATGACATTG

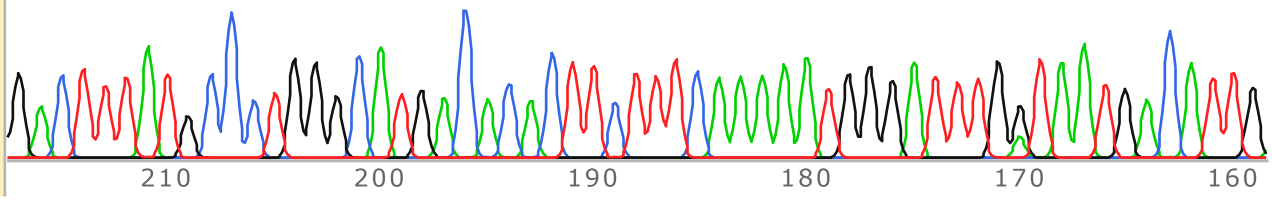

6 ← GACTTTATGCCCTGGGCATGACACACTTCTTTCAAAAATGGGATTTGGTAATGACATTG

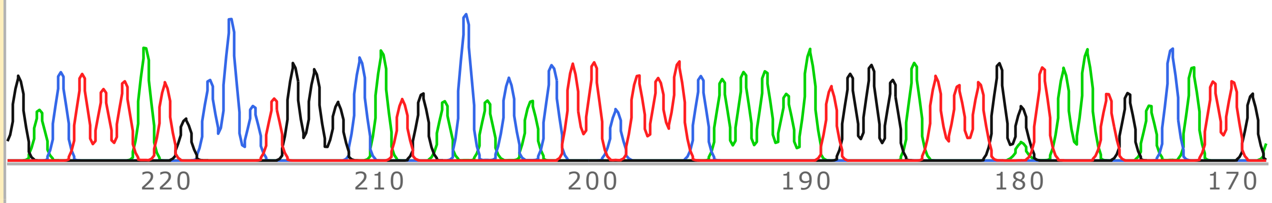

7 ← GACTTTATGCCCTGGGCATGACACACTTCTTTCAAAAATGGGATTTGGTAATGACATTG

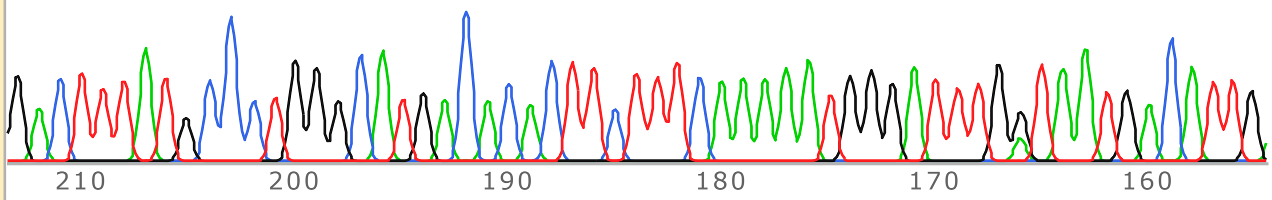

8 ← GACTTTATGCCCTGGGCATGACACACTTCTTTCAAAAATGGGATTTGATAATGACATTG

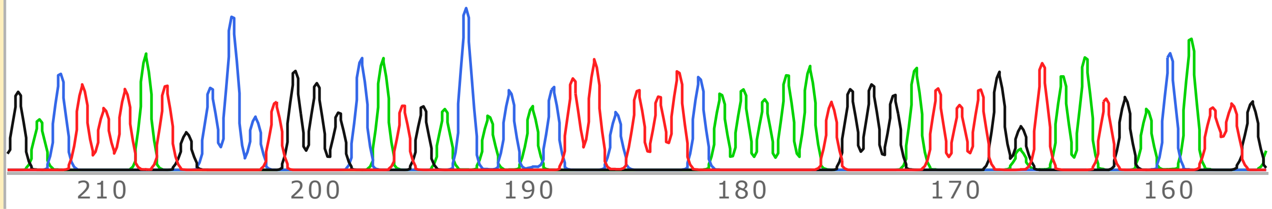

9 ← GACTTTATGCCCTGGGCATGACACACTTCTTTCAAAAATGGGATTTGGTAATGACATTG

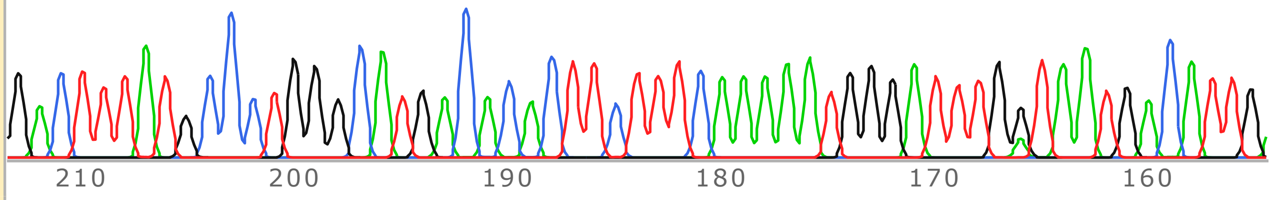

10 ← GACTTTATGCCCTGGGCATGACACACTTCTTTCAAAAATGGGATTTGGTAATGACATTG

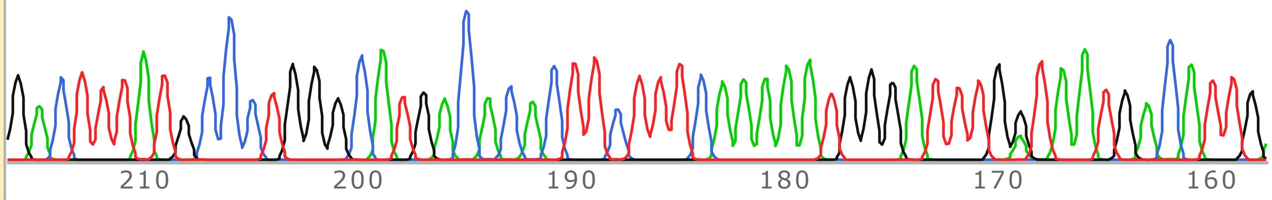

11 ← GACTTTATGCCCTGGGCATGACACACTTCTTTCAAAAATGGGATTTGGTAATGACATTG

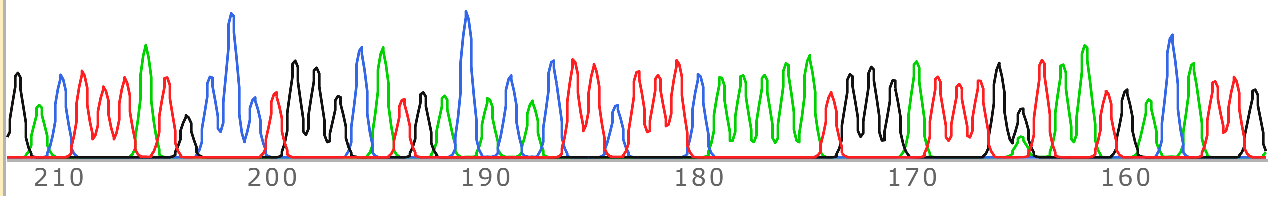

12 GACTTTATGCCCTGGGCATGACACACTTCTTTCAAAAATGGGATTTGGGTAATGACATTG

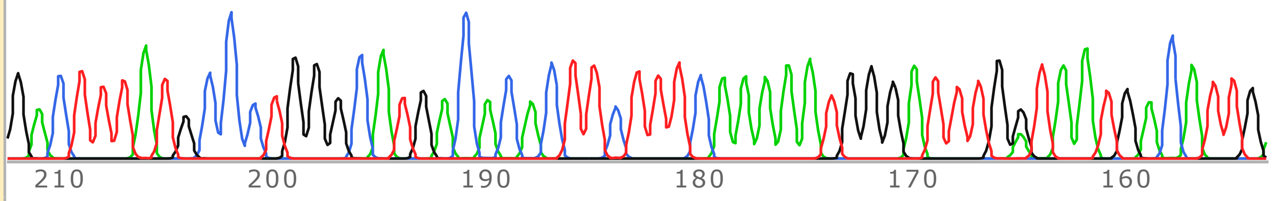

13 GACTTTATGCCCTGGGCATGACACACTTCTTTCAAAAATGGGATTTGGGTAATGACATTG

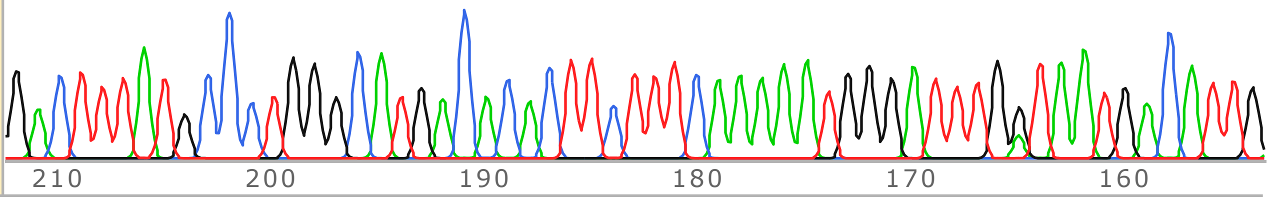

Original Sequence:

1: 293T_WT_OT1_PREMIX_Plate_MFN2_OT_E05
428 bases / Sep 22, 2023
154 .. 212 (1 mismatch)

2: MFN2_G2_OT1_PREMIX_Plate_MFN2_OT_A01
435 bases / Sep 22, 2023
157 .. 215

3: MFN2_G4_OT1_PREMIX_Plate_MFN2_OT_B01
428 bases / Sep 22, 2023
154 .. 212 (1 mismatch)

4: MFN2_G6_OT1_PREMIX_Plate_MFN2_OT_C01
434 bases / Sep 22, 2023
154 .. 212

5: MFN2_G20_OT1_PREMIX_Plate_MFN2_OT_D01
440 bases / Sep 22, 2023
159 .. 217

6: MFN2_G47_OT1_PREMIX_Plate_MFN2_OT_E01
445 bases / Sep 22, 2023
169 .. 227 (1 mismatch)

7: MFN2_G49_OT1_PREMIX_Plate_MFN2_OT_F01
430 bases / Sep 22, 2023
155 .. 213 (1 mismatch)

8: MFN2_T1_OT1_PREMIX_Plate_MFN2_OT_G01
434 bases / Sep 22, 2023
156 .. 214

9: MFN2_T51_OT1_PREMIX_Plate_MFN2_OT_H01
431 bases / Sep 22, 2023
155 .. 213 (1 mismatch)

10: MFN2_T37-3_OT1_PREMIX_Plate_MFN2_OT_A02
435 bases / Sep 22, 2023
158 .. 216 (1 mismatch)

11: MFN2_T37-6_OT1_PREMIX_Plate_MFN2_OT_B02
433 bases / Sep 22, 2023
154 .. 212 (1 mismatch)

12: MFN2_T37-9_OT1_PREMIX_Plate_MFN2_OT_C02
429 bases / Sep 22, 2023
154 .. 212 (1 mismatch)

13: MFN2_T37-12_OT1_PREMIX_Plate_MFN2_OT_D02
433 bases / Sep 22, 2023
154 .. 212 (1 mismatch)

Sequence: MFN2_offtarget2_chr10_95408920.print.ver.dna (Linear / 60 bp)
Features: 1 visible, 1 total

10 20 30 40 50 60

5′ ATCAGAATTGTGCAGCCTGAGACACACATCCTTCAGAAAAGGATACAAGTAATGACATTC 3′

3′ TAGTCTTAACACGTCGGACTCTGTGTGTAGGAAGTCTTTTCCTATGTTCATTACTGTAAG 5′

off-target

ATCAGAATTGTGCAGCCTGAGACACACATCCTTCAGAAAAGGATACAAGTAATGACATTC

1 ATCAGAATTGTGCAGCCTGAGACACACATCCTTCAGAAAAGGATACAAGTAATGACATTC

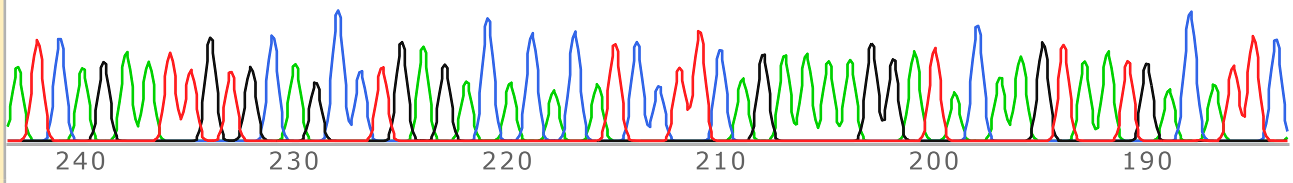

2 ATCAGAATTGTGCAGCCTGAGACACACATCCTTCAGAAAAGGATACAAGTAATGACATTC

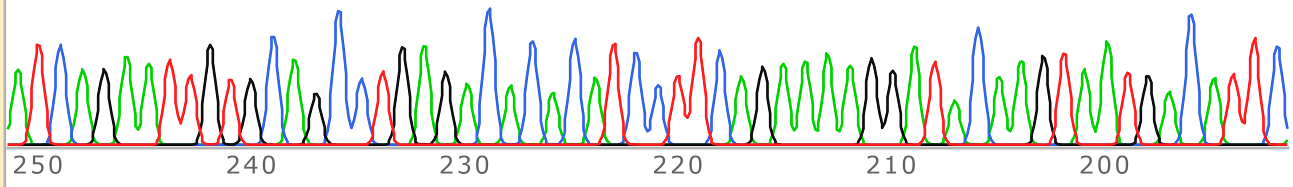

3 ATCAGAATTGTGCAGCCTGAGACACACATCCTTCAGAAAAGGATACAAGTAATGACATTC

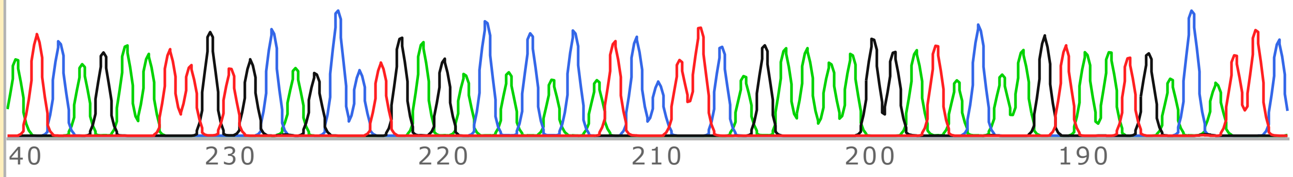

4 ATCAGAATTGTGCAGCCTGAGACACACATCCTTCAGAAAAGGATACAAGTAATGACATTC

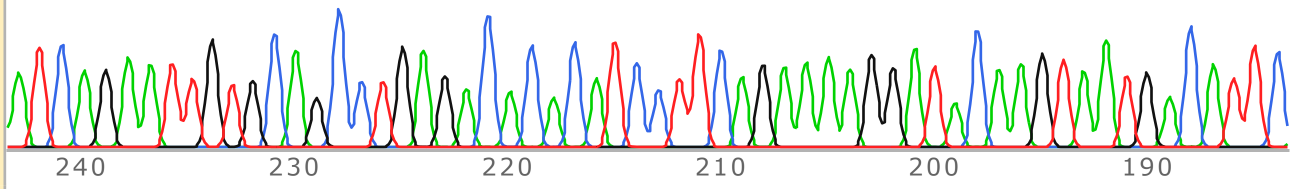

5 ATCAGAATTGTGCAGCCTGAGACACACATCCTTCAGAAAAGGATACAAGTAATGACATTC

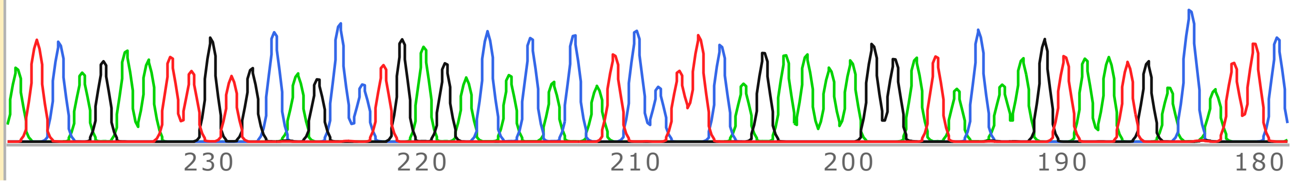

6 ATCAGAATTGTGCAGCCTGAGACACACATCCTTCAGAAAAGGATACAAGTAATGACATTC

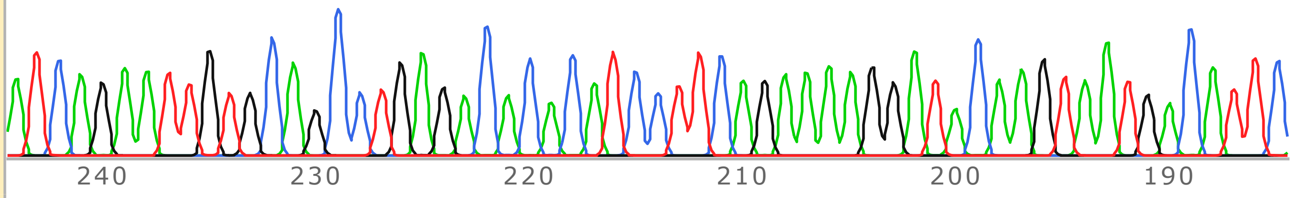

7 ATCAGAATTGTGCAGCCTGAGACACACATCCTTCAGAAAAGGATACAAGTAATGACATTC

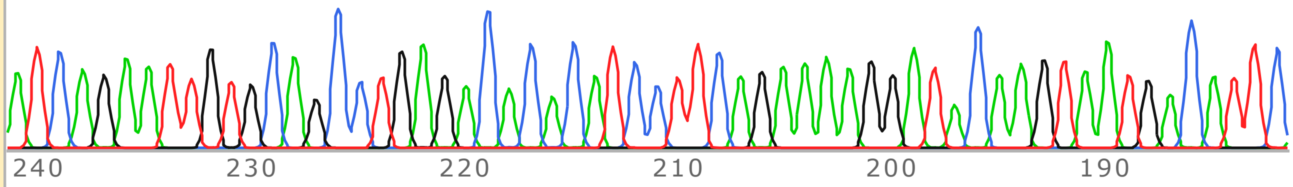

8 ATCAGAATTGTGCAGCCTGAGACACACATCCTTCAGAAAAGGATACAAGTAATGACATTC

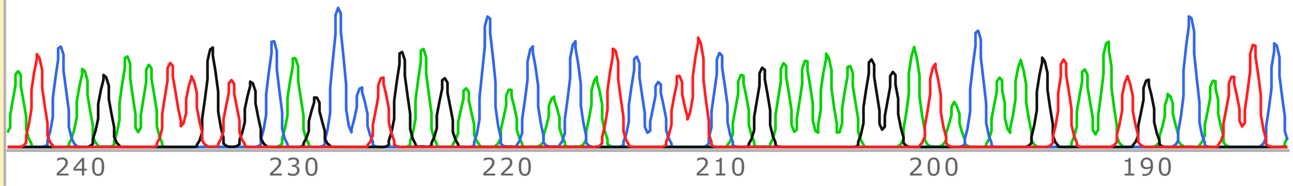

9 ATCAGAATTGTGCAGCCTGAGACACACATCCTTCAGAAAAGGATACAAGTAATGACATTC

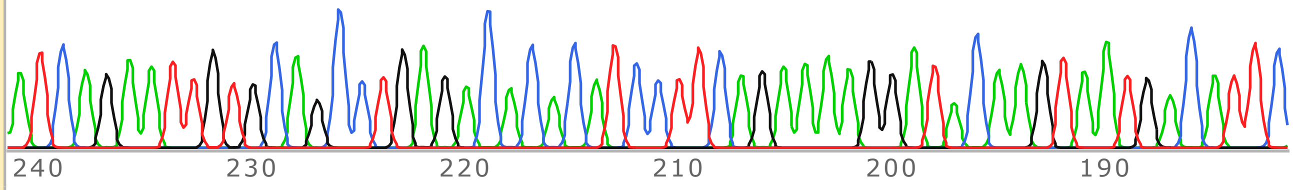

10 ATCAGAATTGTGCAGCCTGAGACACACATCCTTCAGAAAAGGATACAAGTAATGACATTC

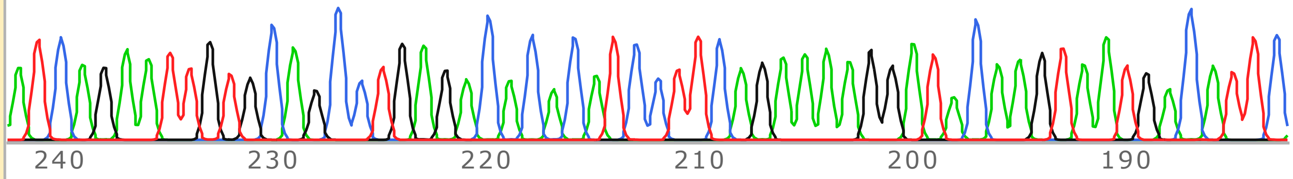

11 ATCAGAATTGTGCAGCCTGAGACACACATCCTTCAGAAAAGGATACAAGTAATGACATTC

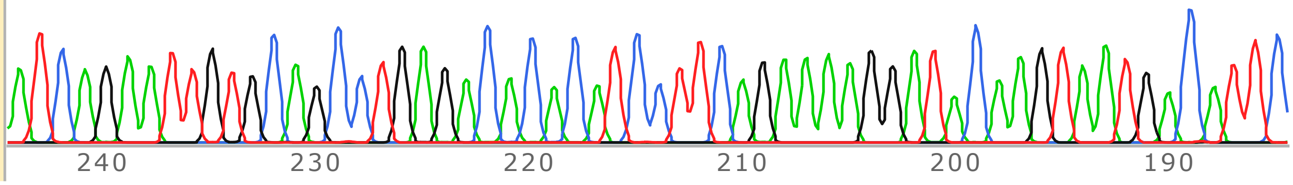

12 ATCAGAATTGTGCAGCCTGAGACACACATCCTTCAGAAAAGGATACAAGTAATGACATTC

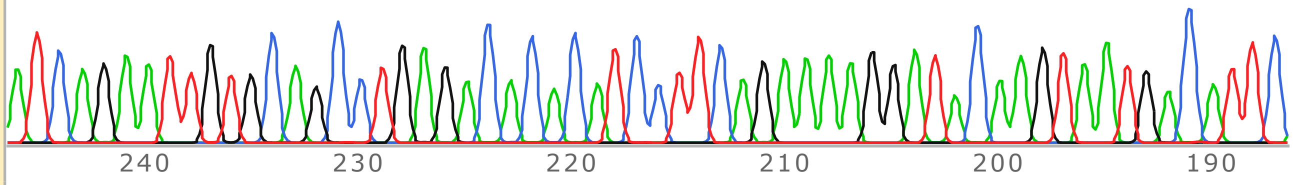

13 ATCAGAATTGTGCAGCCTGAGACACACATCCTTCAGAAAAGGATACAAGTAATGACATTC

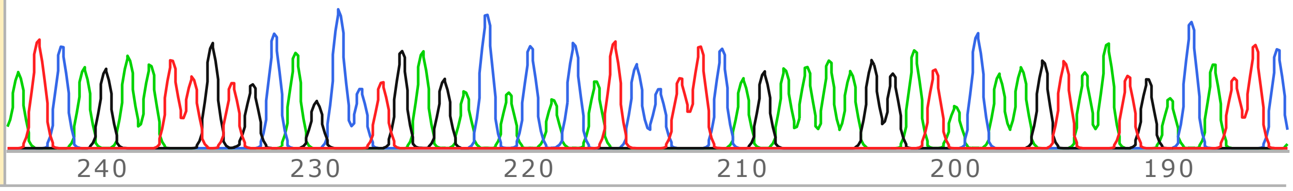

Original Sequence:

1: 293T_WT_OT2_PREMIX_Plate_MFN2_OT_F05
473 bases / Sep 22, 2023
184 .. 243

2: MFN2_G2_OT2_PREMIX_Plate_MFN2_OT_E02
482 bases / Sep 22, 2023
192 .. 251

3: MFN2_G4_OT2_PREMIX_Plate_MFN2_OT_F02
467 bases / Sep 22, 2023
181 .. 240

4: MFN2_G6_OT2_PREMIX_Plate_MFN2_OT_G02
472 bases / Sep 22, 2023
184 .. 243

5: MFN2_G20_OT2_PREMIX_Plate_MFN2_OT_H02
465 bases / Sep 22, 2023
180 .. 239

6: MFN2_G47_OT2_PREMIX_Plate_MFN2_OT_A03
469 bases / Sep 22, 2023
185 .. 244

7: MFN2_G49_OT2_PREMIX_Plate_MFN2_OT_B03
471 bases / Sep 22, 2023
182 .. 241

8: MFN2_T1_OT2_PREMIX_Plate_MFN2_OT_C03
473 bases / Sep 22, 2023
184 .. 243

9: MFN2_T51_OT2_PREMIX_Plate_MFN2_OT_D03
470 bases / Sep 22, 2023
182 .. 241

10: MFN2_T37-3_OT2_PREMIX_Plate_MFN2_OT_E03
473 bases / Sep 22, 2023
183 .. 242

11: MFN2_T37-6_OT2_PREMIX_Plate_MFN2_OT_F03
473 bases / Sep 22, 2023
185 .. 244

12: MFN2_T37-9_OT2_PREMIX_Plate_MFN2_OT_G03
476 bases / Sep 22, 2023
187 .. 246

13: MFN2_T37-12_OT2_PREMIX_Plate_MFN2_OT_H03
472 bases / Sep 22, 2023
185 .. 244

Sequence: MFN2_offtarget3_chrX_4717017.print.ver.dna (Linear / 58 bp)
Features: 1 visible, 1 total

10 20 30 40 50

5′ TTATCCAGTATCACATTATCAATATTTCTTTCAGAAATGGAGACCATCGTAGTCCATA 3′

3′ AATAGGTCATAGTGTAATAGTTATAAAGAAAGTCTTTACCTCTGGTAGCATCAGGTAT 5′

off-target

TTATCCAGTATCACATTATCAATATTTCTTTCAGAAATGGAGACCATCGTAGTCCATA

1 TTATCCAGTATCACATTATCAATATTTCTTTCAGAAATGGAGACCATCGTAGTCCATA

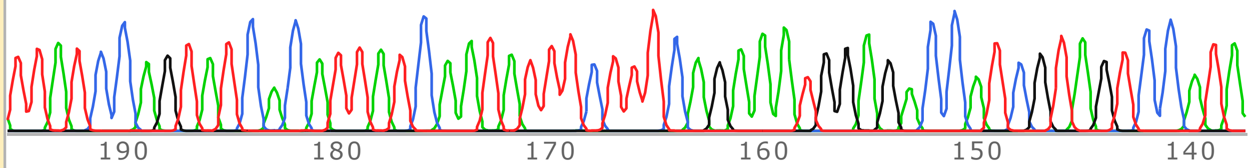

2 TTATCCAGTATCACATTATCAATATTTCTTTCAGAAATGGAGACCATCGTAGTCCATA

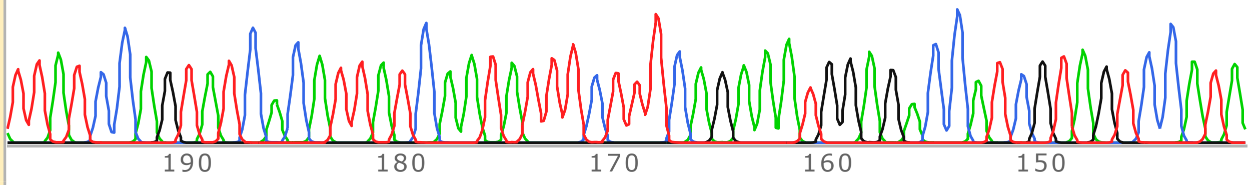

3 TTATCCAGTATCACATTATCAATATTTCTTTCAGAAATGGAGACCATCGTAGTCCATA

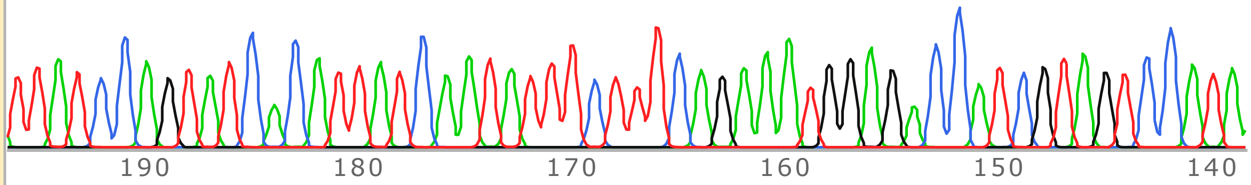

4 TTATCCAGTATCACATTATCAATATTTCTTTCAGAAATGGAGACCATCGTAGTCCATA

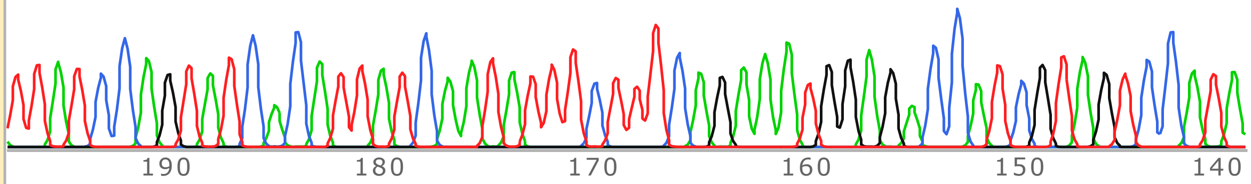

5 TTATCCAGTATCACATTATCAATATTTCTTTCAGAAATGGAGACCATCGTAGTCCATA

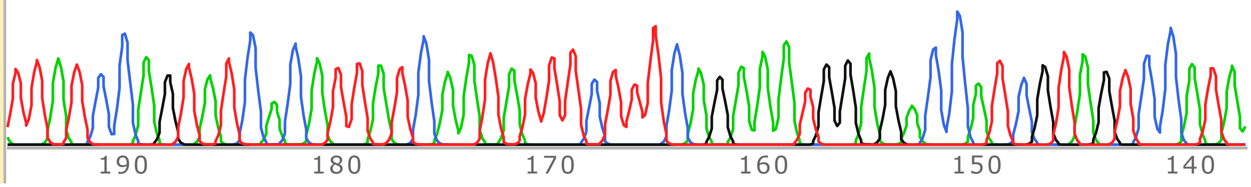

6 TTATCCAGTATCACATTATCAATATTTCTTTCAGAAATGGAGACCATCGTAGTCCATA

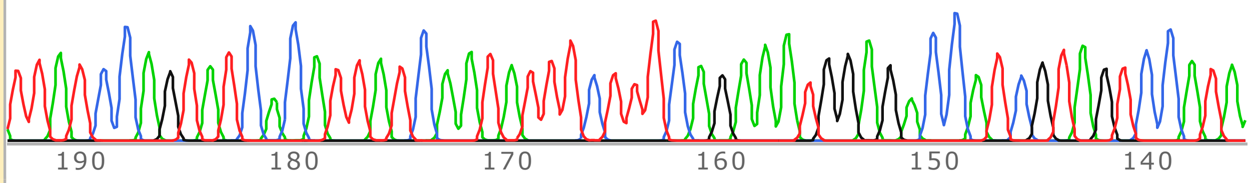

7 TTATCCAGTATCACATTATCAATATTTCTTTCAGAAATGGAGACCATCGTAGTCCATA

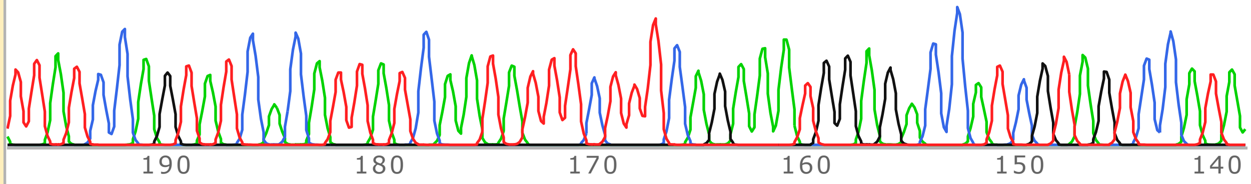

8 TTATCCAGTATCACATTATCAATATTTCTTTCAGAAATGGAGACCATCGTAGTCCATA

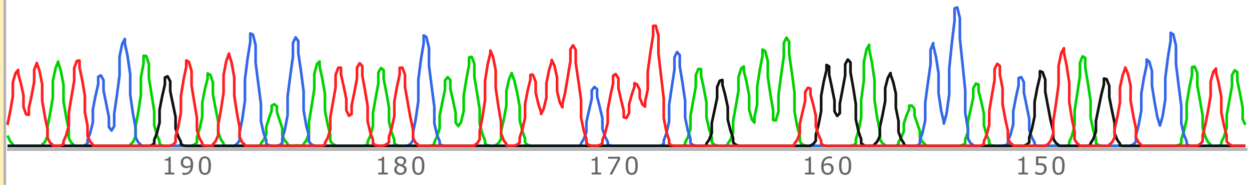

9 TTATCCAGTATCACATTATCAATATTTCTTTCAGAAATGGAGACCATCGTAGTCCATA

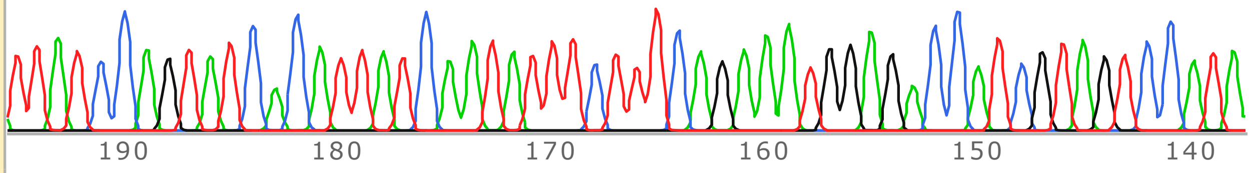

10 TTATCCAGTATCACATTATCAATATTTCTTTCAGAAATGGAGACCATCGTAGTCCATA

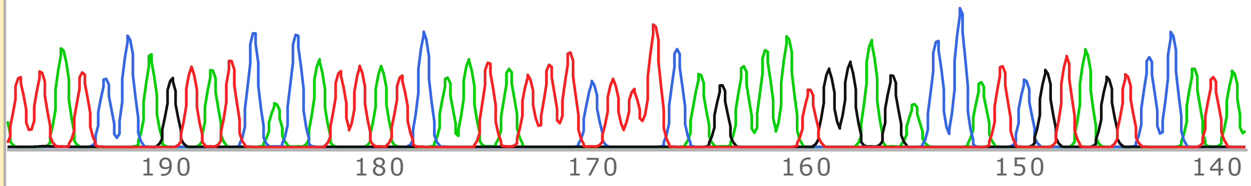

11 TTATCCAGTATCACATTATCAATATTTCTTTCAGAAATGGAGACCATCGTAGTCCATA

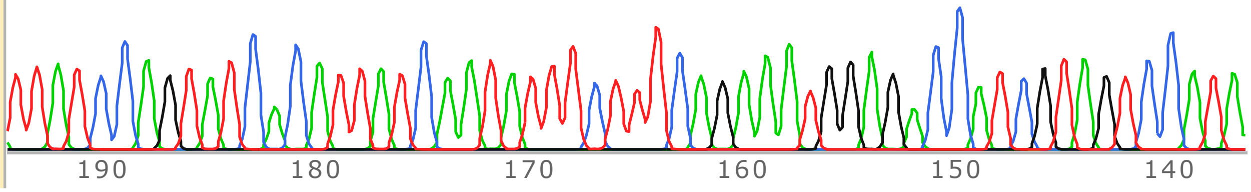

12 TTATCCAGTATCACATTATCAATATTTCTTTCAGAAATGGAGACCATCGTAGTCCATA

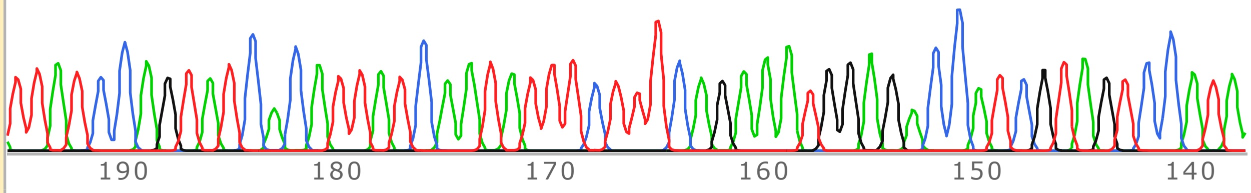

13 TTATCCAGTATCACATTATCAATATTTCTTTCAGAAATGGAGACCATCGTAGTCCATA

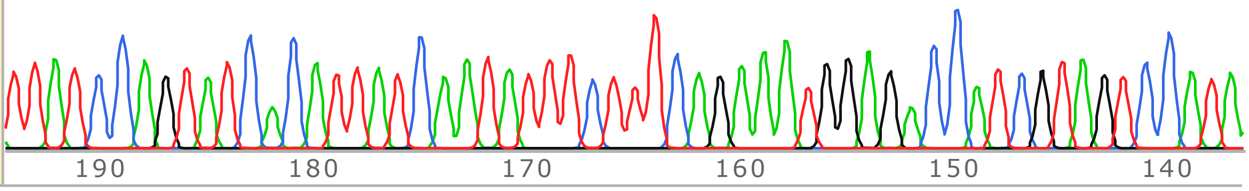

Original Sequence:

1: 293T_WT_OT3_PREMIX_Plate_MFN2_OT_G05
418 bases / Sep 22, 2023
138 .. 195

2: MFN2_G2_OT3_PREMIX_Plate_MFN2_OT_A04
420 bases / Sep 22, 2023
141 .. 198

3: MFN2_G4_OT3_PREMIX_Plate_MFN2_OT_B04
417 bases / Sep 22, 2023
139 .. 196

4: MFN2_G6_OT3_PREMIX_Plate_MFN2_OT_C04
419 bases / Sep 22, 2023
140 .. 197

5: MFN2_G20_OT3_PREMIX_Plate_MFN2_OT_D04
419 bases / Sep 22, 2023
138 .. 195

6: MFN2_G47_OT3_PREMIX_Plate_MFN2_OT_E04
419 bases / Sep 22, 2023
136 .. 193

7: MFN2_G49_OT3_PREMIX_Plate_MFN2_OT_F04
421 bases / Sep 22, 2023
140 .. 197

8: MFN2_T1_OT3_PREMIX_Plate_MFN2_OT_G04
421 bases / Sep 22, 2023
141 .. 198

9: MFN2_T51_OT3_PREMIX_Plate_MFN2_OT_H04
417 bases / Sep 22, 2023
138 .. 195

10: MFN2_T37-3_OT3_PREMIX_Plate_MFN2_OT_A05
434 bases / Sep 22, 2023
140 .. 197

11: MFN2_T37-6_OT3_PREMIX_Plate_MFN2_OT_B05
415 bases / Sep 22, 2023
137 .. 194

12: MFN2_T37-9_OT3_PREMIX_Plate_MFN2_OT_C05
416 bases / Sep 22, 2023
138 .. 195

13: MFN2_T37-12_OT3_PREMIX_Plate_MFN2_OT_D05
415 bases / Sep 22, 2023
137 .. 194

Sequence: FOSL2_offtarget1_chr16_31090772.print.ver.dna (Linear / 59 bp)
Features: 1 visible, 1 total

10 20 30 40 50

5′ CCACTCTCCCTCTGACTCACCCTTCCCAGCTCCAGAGAAGGCAACACCGAGGGAGGCCC 3′

3′ GGTGAGAGGGAGACTGAGTGGGAAGGGTCGAGGTCTCTTCCGTTGTGGCTCCCTCCGGG 5′

offtarget

CCACTCTCCCTCTGACTCACCCTTCCCAGCTCCAGAGAAGGCAACACCGAGGGAGGCCC

1 CCACTCTCCCTCTGACTCACCCTTCCCAGCTCCAGAGAAGGCAACACCGAGGGAGGCCC

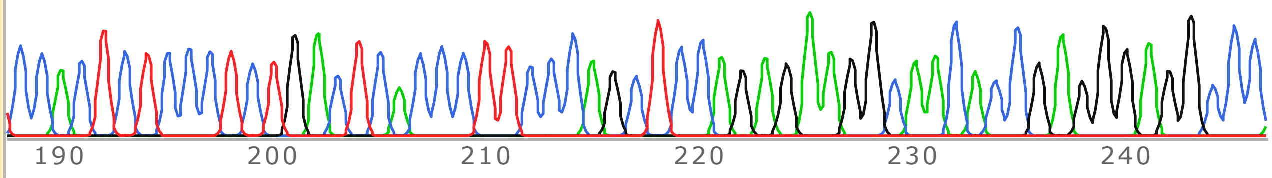

2 CCACTCTCCCTCTGACTCACCCTTCCCAGCTCCAGAGAAGGCAACACCGAGGGAGGCCC

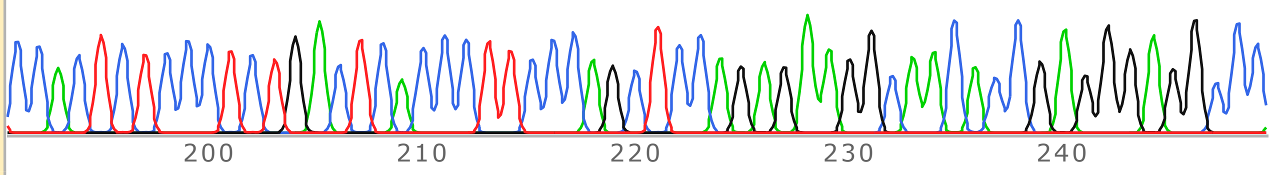

3 CCACTCTCCCTCTGACTCACCCTTCCCAGCTCCAGAGAAGGCAACACCGAGGGAGGCCC

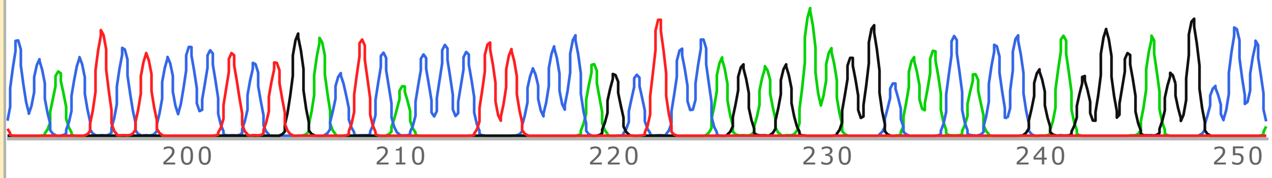

4 CCACTCTCCCTCTGACTCACCCTTCCCAGCTCCAGAGAAGGCAACACCGAGGGAGGCCC

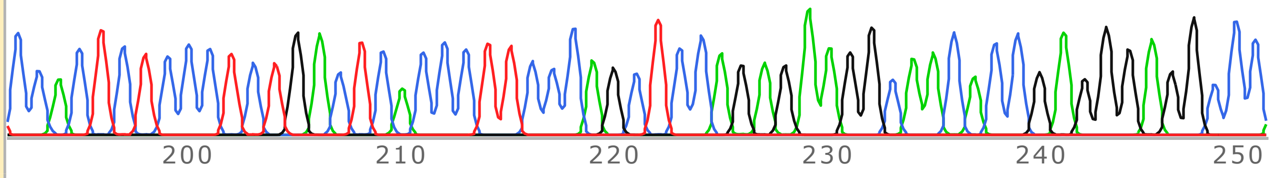

5 CCACTCTCCCTCTGACTCACCCTTCCCAGCTCCAGAGAAGGCAACACCGAGGGAGGCCC

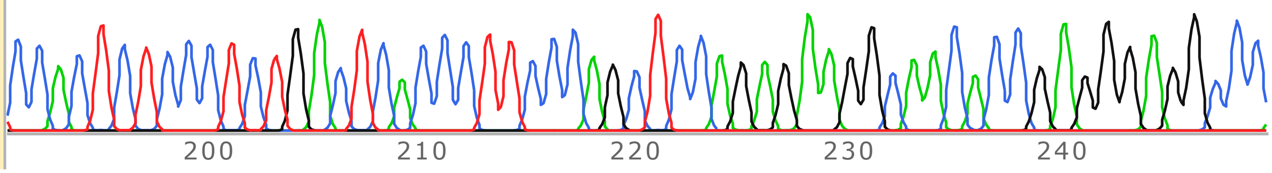

6 CCACTCTCCCTCTGACTCACCCTTCCCAGCTCCAGAGAAGGCAACACCGAGGGAGGCCC

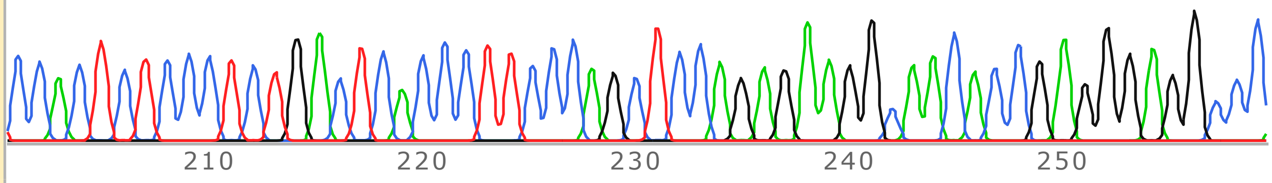

7 CCACTCTCCCTCTGACTCACCCTTCCCAGCTCCAGAGAAGGCAACACCGAGGGAGGCCC

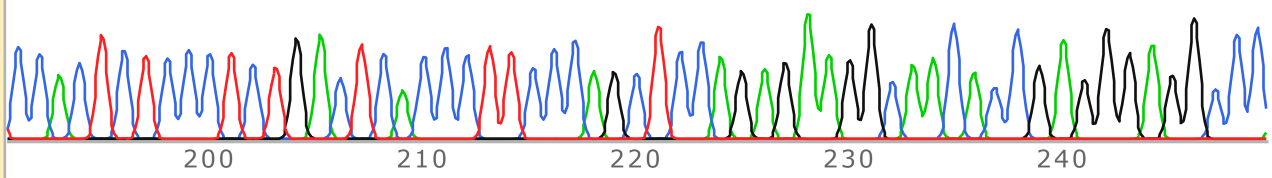

8 CCACTCTCCCTCTGACTCACCCTTCCCAGCTCCAGAGAAGGCAACACCGAGGGAGGCCC

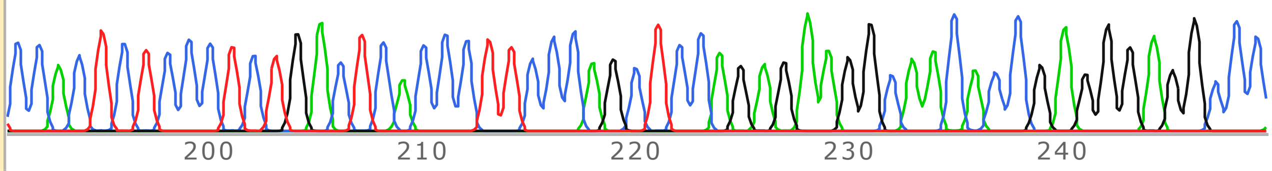

9 CCACTCTCCCTCTGACTCACCCTTCCCAGCTCCAGAGAAGGCAACACCGAGGGAGGCCC

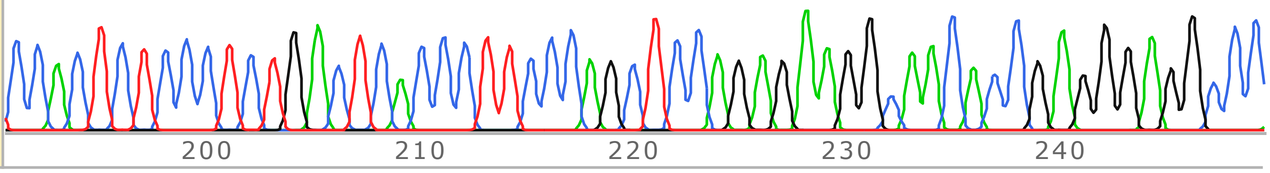

Original Sequence:

1: HEK293T_WT_OT1_PREMIX_Plate_Plate01_A06
481 bases / Sep 23, 2023
188 .. 246

2: FOSL2_G3_OT1_PREMIX_Plate_Plate01_A01
484 bases / Sep 23, 2023
191 .. 249

3: FOSL2_G8_OT1_PREMIX_Plate_Plate01_B01
485 bases / Sep 23, 2023
192 .. 250

4: FOSL2_G16_OT1_PREMIX_Plate_Plate01_C01
484 bases / Sep 23, 2023
192 .. 250

5: FOSL2_G17_OT1_PREMIX_Plate_Plate01_D01
484 bases / Sep 23, 2023
191 .. 249

6: FOSL2_A4_OT1_PREMIX_Plate_Plate01_E01
492 bases / Sep 23, 2023
201 .. 259

7: FOSL2_A7-1_OT1_PREMIX_Plate_Plate01_G01
482 bases / Sep 23, 2023
191 .. 249

8: FOSL2_A7-2_OT1_PREMIX_Plate_Plate01_H01
483 bases / Sep 23, 2023
191 .. 249

9: FOSL2_A20_OT1_PREMIX_Plate_Plate01_F01
482 bases / Sep 23, 2023
191 .. 249

Sequence: FOSL2_offtarget2_chr16_31092500.print.ver.dna (Linear / 59 bp)
Features: 1 visible, 1 total

10 20 30 40 50

5′ CCACGCTCCCTTTGAGTCACCCTTCCCAGCTCCAGAGAAGGCATCACTGAGGGAGGCCC 3′

3′ GGTGCGAGGGAAACTCAGTGGGAAGGGTCGAGGTCTCTTCCGTAGTGACTCCCTCCGGG 5′

offtarget

CCACGCTCCCTTTGAGTCACCCTTCCCAGCTCCAGAGAAGGCATCACTGAGGGAGGCCC

1 CCACGCTCCCTTTGAGTCACCCTTCCCAGCTCCAGAGAAGGCATCACTGAGGGAGGCCC

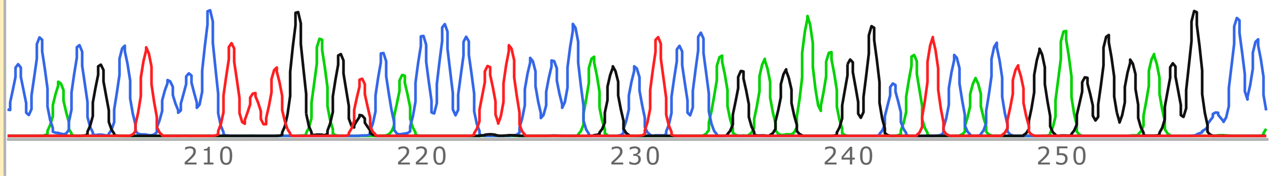

2 CCACGCTCCCTTTGAGTCACCCTTCCCAGCTCCAGAGAAGGCATCACTGAGGGAGGCCC

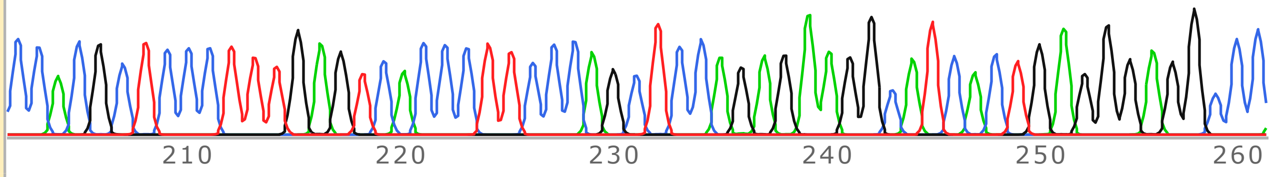

3 CCACGCTCCCTTTGAGTCACCCTTCCCAGCTCCAGAGAAGGCATCACTGAGGGAGGCCC

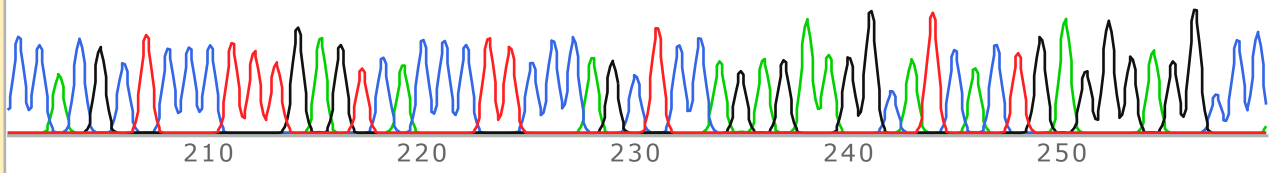

4 CCACGCTCCCTTTGAGTCACCCTTCCCAGCTCCAGAGAAGGCATCACTGAGGGAGGCCC

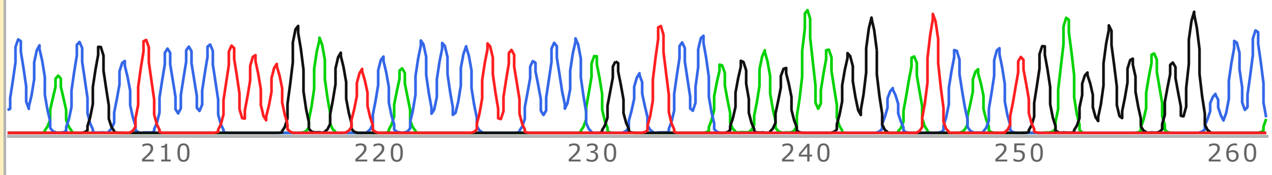

5 CCACGCTCCCTTTGAGTCACCCTTCCCAGCTCCAGAGAAGGCATCACTGAGGGAGGCCC

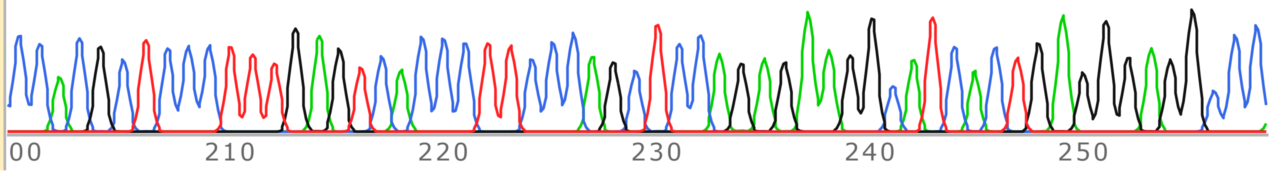

6 CCACGCTCCCTTTGAGTCACCCTTCCCAGCTCCAGAGAAGGCATCACTGAGGGAGGCCC

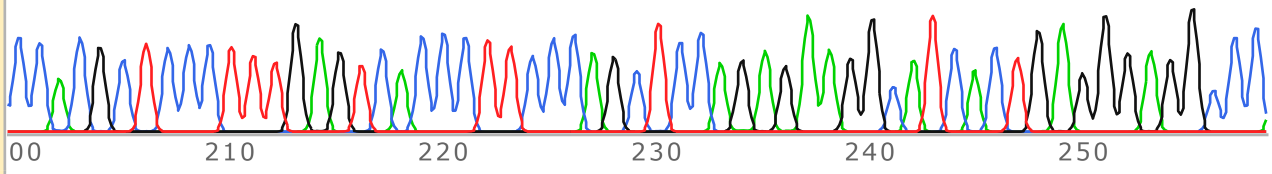

7 CCACGCTCCCTTTGAGTCACCCTTCCCAGCTCCAGAGAAGGCATCACTGAGGGAGGCCC

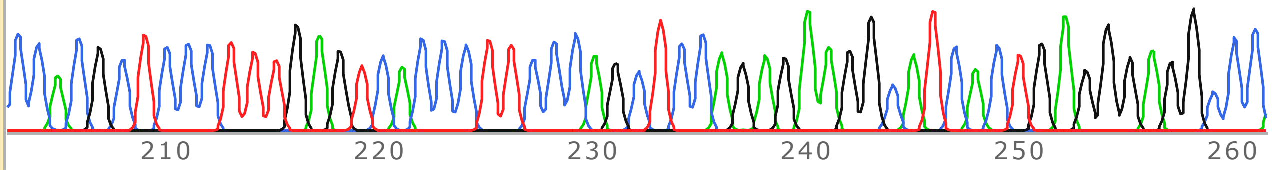

8 CCACGCTCCCTTTGAGTCACCCTTCCCAGCTCCAGAGAAGGCATCACTGAGGGAGGCCC

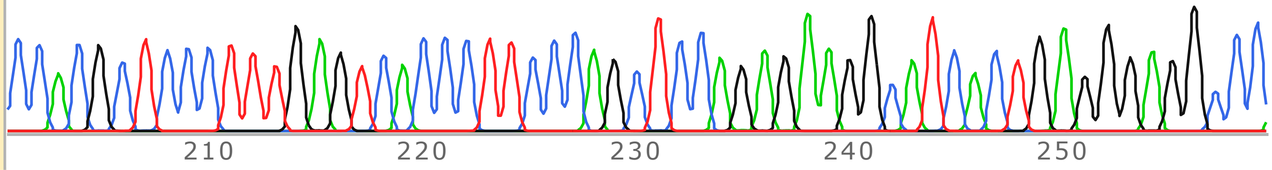

9 CCACGCTCCCTTTGAGTCACCCTTCCCAGCTCCAGAGAAGGCATCACTGAGGGAGGCCC

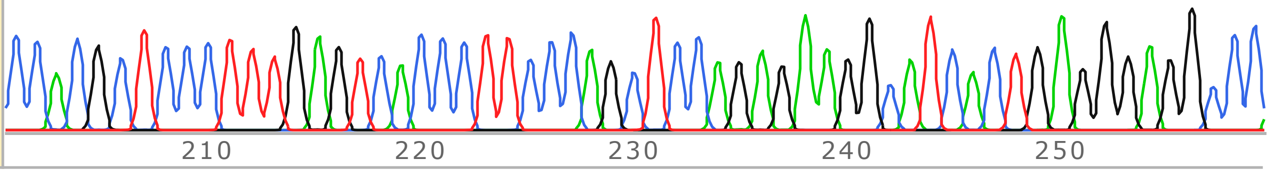

Original Sequence:

1: HEK293T_WT_OT2_PREMIX_Plate_Plate01_B06
700 bases / Sep 23, 2023
201 .. 259

2: FOSL2_G3_OT2_PREMIX_Plate_Plate01_A01
496 bases / Sep 28, 2023
202 .. 260

3: FOSL2_G8_OT2_PREMIX_Plate_Plate01_B01
495 bases / Sep 28, 2023
201 .. 259

4: FOSL2_G16_OT2_PREMIX_Plate_Plate01_C01
498 bases / Sep 28, 2023
203 .. 261

5: FOSL2_G17_OT2_PREMIX_Plate_Plate01_D01
495 bases / Sep 28, 2023
200 .. 258

6: FOSL2_A4_OT2_PREMIX_Plate_Plate01_E01
495 bases / Sep 28, 2023
200 .. 258

7: FOSL2_A7-1_OT2_PREMIX_Plate_Plate01_G01
497 bases / Sep 28, 2023
203 .. 261

8: FOSL2_A7-2_OT2_PREMIX_Plate_Plate01_H01
495 bases / Sep 28, 2023
201 .. 259

9: FOSL2_A20_OT2_PREMIX_Plate_Plate01_F01
495 bases / Sep 28, 2023
201 .. 259

Sequence: FOSL2_offtarget3_chr19_2294399.print.ver.dna (Linear / 58 bp)
Features: 1 visible, 1 total

10 20 30 40 50

5′ AAAACAACCGAAATATATGCCTTCCCAGCTCCGGAGGCGGAAGTCCAAAATCAAGGTG 3′

3′ TTTTGTTGGCTTTATATACGGAAGGGTCGAGGCCTCCGCCTTCAGGTTTTAGTTCCAC 5′

offtarget

AAAACAACCGAAATATATGCCTTCCCAGCTCCGGAGGCGGAAGTCCAAAATCAAGGTG

1 ➡ AAAACAACCGAAATATATGCCTTCCCAGCTCCGGAGGCGGAAGTCCAAAATCAAGGTG

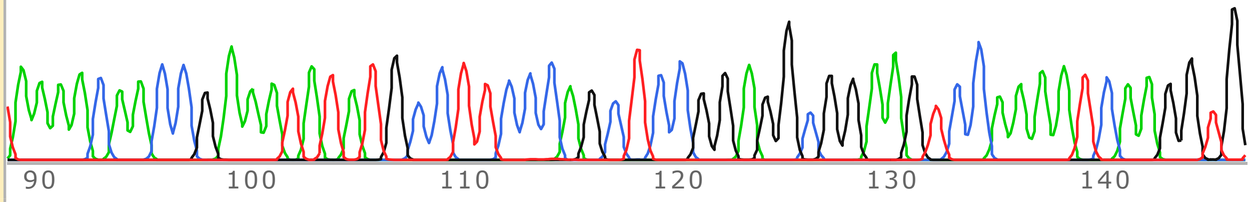

2 ➡ AAAACAACCGAAATATATGCCTTCCCAGCTCCGGAGGCGGAAGTCCAAAATCAAGGTG

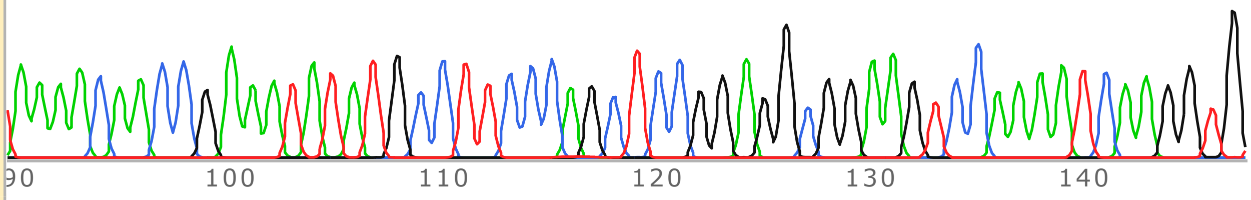

3 ➡ AAAACAACCGAAATATATGCCTTCCCAGCTCCGGAGGCGGAAGTCCAAAATCAAGGTG

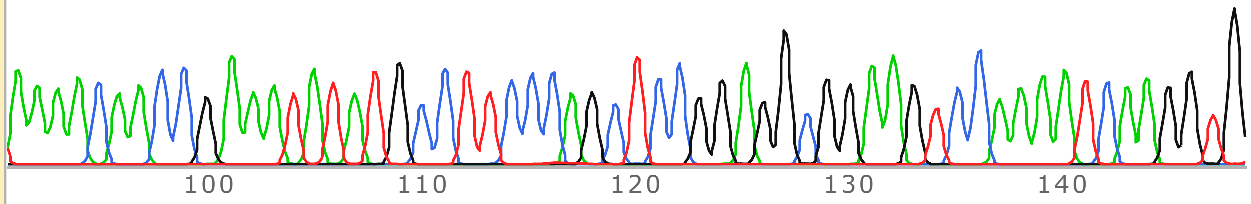

4 ➡ AAAACAACCGAAATATATGCCTTCCCAGCTCCGGAGGCGGAAGTCCAAAATCAAGGTG

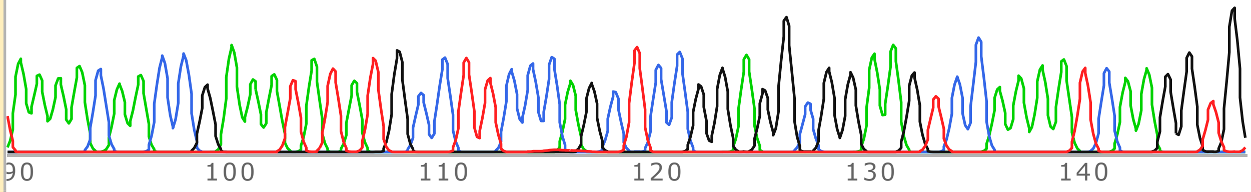

5 ➡ AAAACAACCGAAATATATGCCTTCCCAGCTCCGGAGGCGGAAGTCCAAAATCAAGGTG

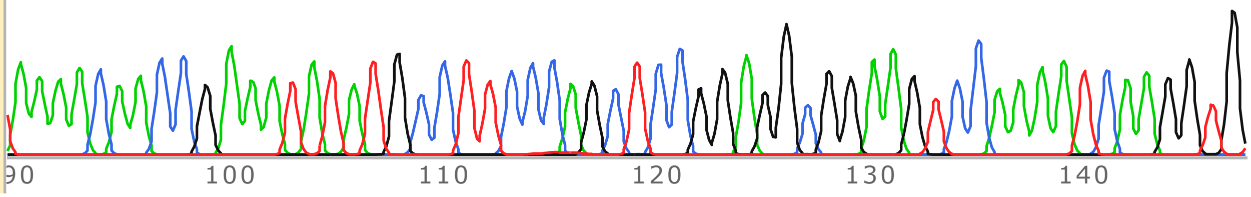

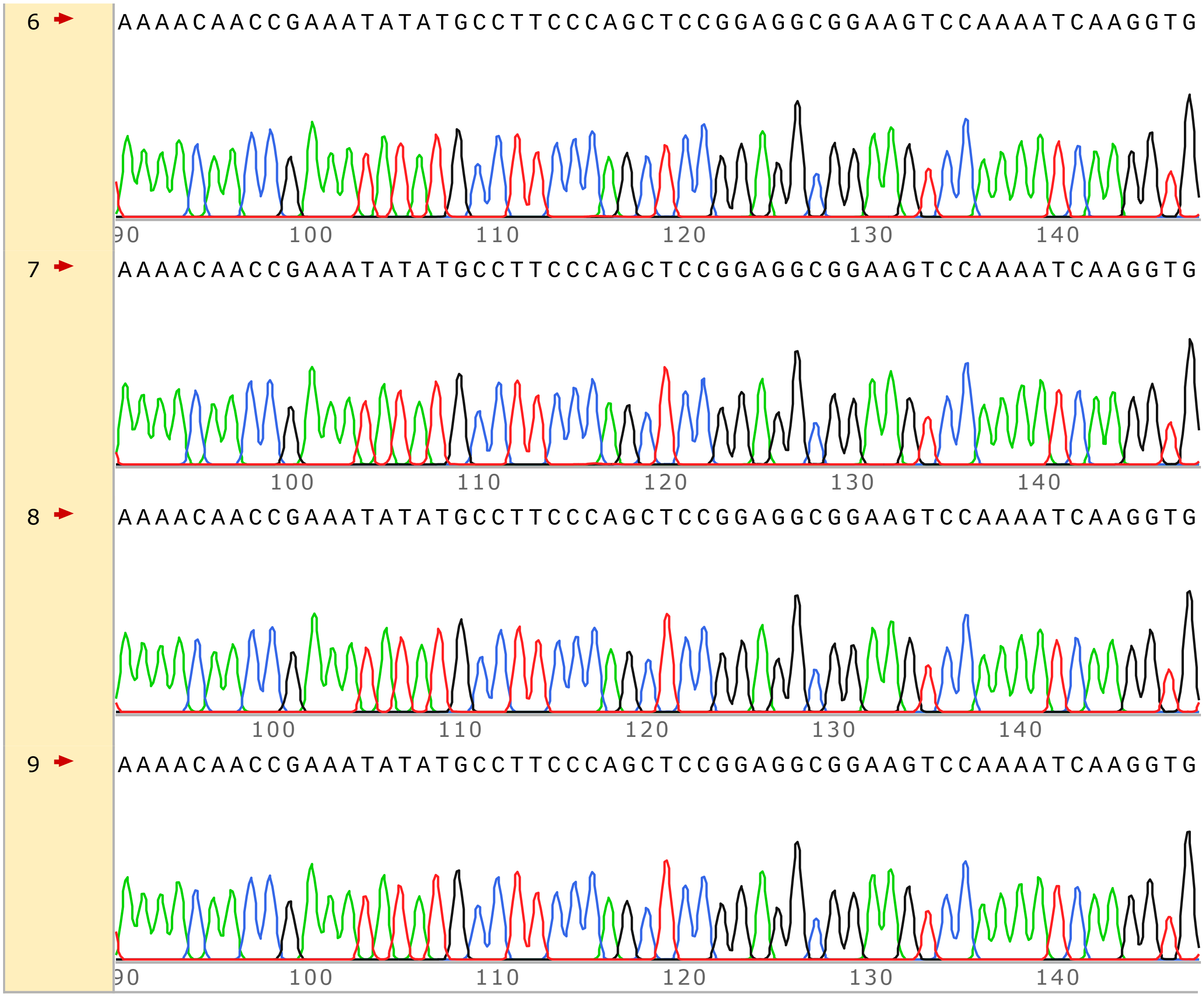
6
AAAACAACCGAAATATATGCCTTCCCAGCTCCGGAGGCGGAAGTCCAAAATCAAGGTG
90 100 110 120 130 140
7
AAAACAACCGAAATATATGCCTTCCCAGCTCCGGAGGCGGAAGTCCAAAATCAAGGTG
100 110 120 130 140
8
AAAACAACCGAAATATATGCCTTCCCAGCTCCGGAGGCGGAAGTCCAAAATCAAGGTG
100 110 120 130 140
9
AAAACAACCGAAATATATGCCTTCCCAGCTCCGGAGGCGGAAGTCCAAAATCAAGGTG
90 100 110 120 130 140

Original Sequence:

1: HEK293T_WT_OT3_PREMIX_Plate_Plate01_B05
303 bases / Sep 28, 2023
89 .. 146

2: FOSL2_G3_OT3_F_PREMIX_A10
302 bases / Sep 29, 2023
90 .. 147

3: FOSL2_G8_OT3_PREMIX_Plate_Plate01_B02
303 bases / Sep 28, 2023
91 .. 148

4: FOSL2_G16_OT3_PREMIX_Plate_Plate01_C02
303 bases / Sep 28, 2023
90 .. 147

5: FOSL2_G17_OT3_PREMIX_Plate_Plate01_D02
302 bases / Sep 28, 2023
90 .. 147

6: FOSL2_A4_OT3_PREMIX_Plate_Plate01_E02
301 bases / Sep 28, 2023
90 .. 147

7: FOSL2_A7-1_OT3_PREMIX_Plate_Plate01_G02
304 bases / Sep 28, 2023
91 .. 148

8: FOSL2_A7-2_OT3_PREMIX_Plate_Plate01_H02
307 bases / Sep 28, 2023
92 .. 149

9: FOSL2_A20_OT3_F_PREMIX_C10
302 bases / Sep 29, 2023
90 .. 147

Sequence: FOSL2_offtarget4_chrX_29617781.print.ver.dna (Linear / 59 bp)
Features: 1 visible, 1 total

10 20 30 40 50

5′ GTATTAATAGGGATCTAGGGAGTGAAGTGGACTACCTCGGGCATTCTTACTTAAATAAC 3′

3′ CATAATTATCCCTAGATCCCTCACTTCACCTGATGGAGCCCGTAAGAATGAATTTATTG 5′

offtarget

GTATTAATAGGGATCTAGGGAGTGAAGTGGACTACCTCGGGCATTCTTACTTAAATAAC

1 GTATTAATAGGGATCTAGGGAGTGAAGTGGACTACCTCGGGCATTCTTACTTAAATAAC

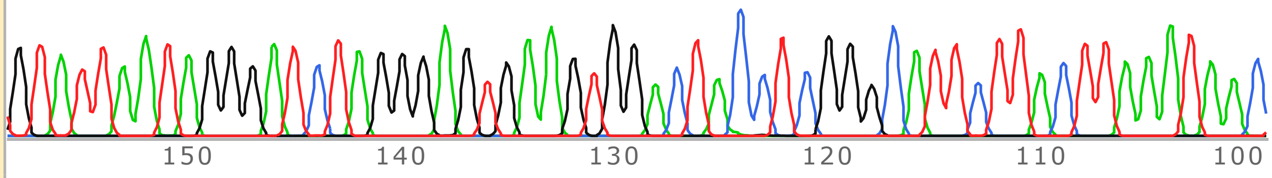

2 GTATTAATAGGGATCTAGGGAGTGAAGTGGACTACCTCGGGCATTCTTACTTAAATAAC

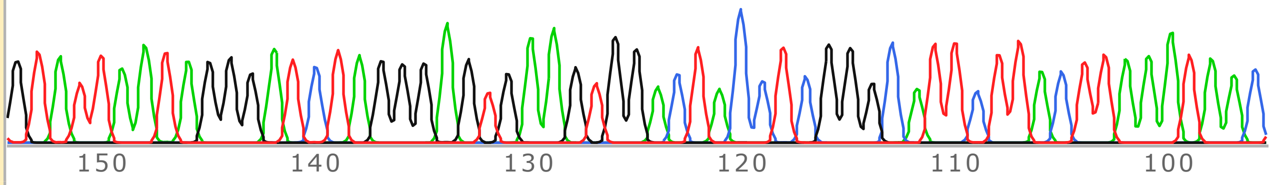

3 GTATTAATAGGGATCTAGGGAGTGAAGTGGACTACCTCGGGCATTCTTACTTAAATAAC

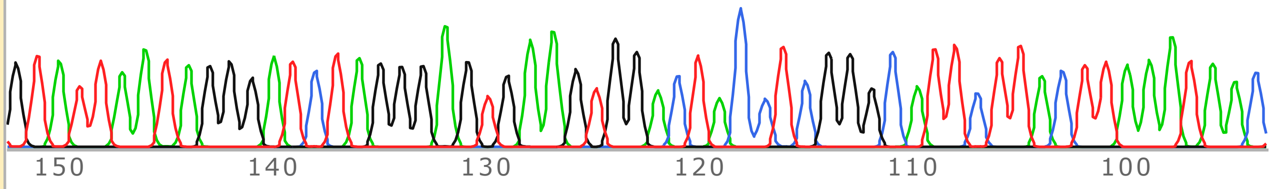

4 GTATTAATAGGGATCTAGGGAGTGAAGTGGACTACCTCGGGCATTCTTACTTAAATAAC

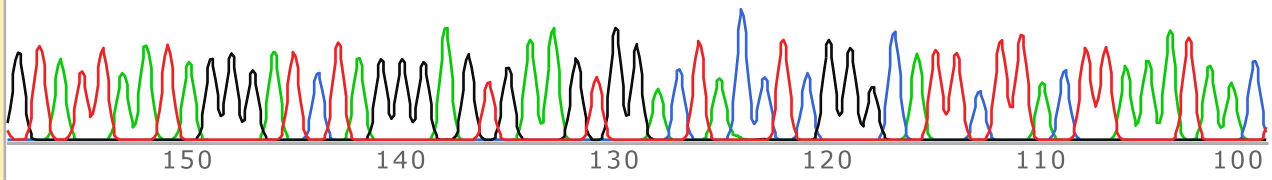

5 GTATTAATAGGGATCTAGGGAGTGAAGTGGACTACCTCGGGCATTCTTACTTAAATAAC

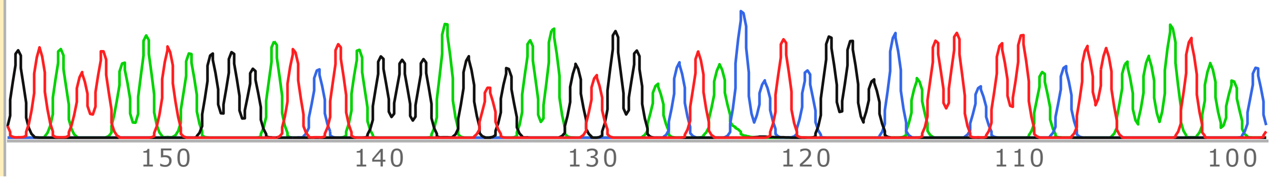

6 ← GTATTAATAGGGATCTAGGGAGTGAAGTGGACTACCTCGGGCATTCTTACTTAAATAAC

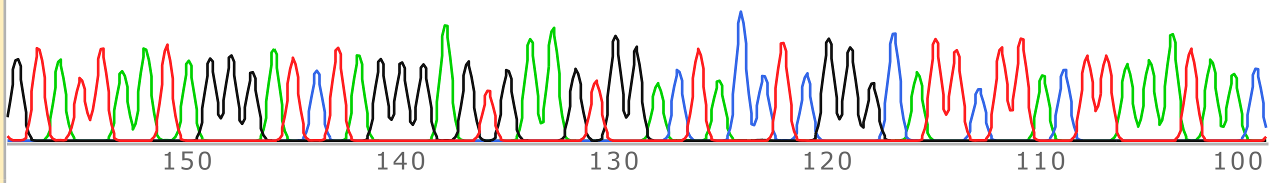

7 ← GTATTAATAGGGATCTAGGGAGTGAAGTGGACTACCTCGGGCATTCTTACTTAAATAAC

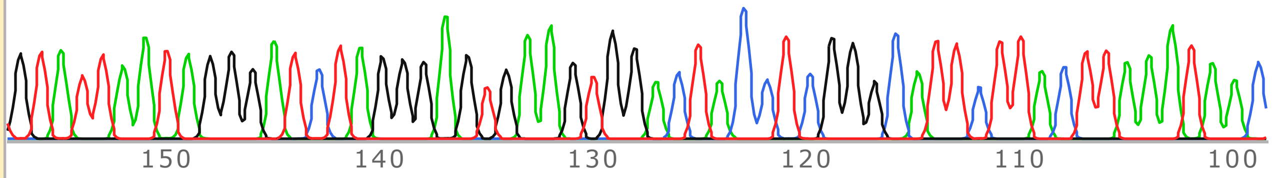

8 ← GTATTAATAGGGATCTAGGGAGTGAAGTGGACTACCTCGGGCATTCTTACTTAAATAAC

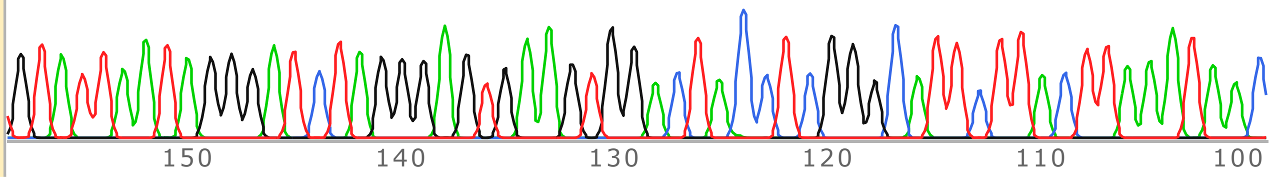

9 ← GTATTAATAGGGATCTAGGGAGTGAAGTGGACTACCTCGGGCATTCTTACTTAAATAAC

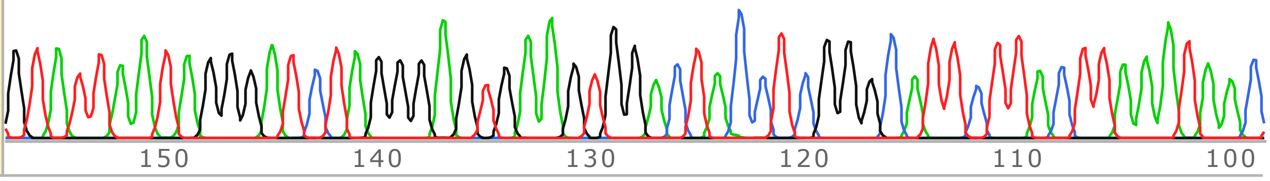

Original Sequence:

1: HEK293T_WT_OT4_PREMIX_Plate_Plate01_C05
271 bases / Sep 28, 2023
100 .. 158

2: FOSL2_G3_OT4_R_PREMIX_F10
266 bases / Sep 29, 2023
96 .. 154

3: FOSL2_G8_OT4_R_PREMIX_H10
264 bases / Sep 29, 2023
94 .. 152

4: FOSL2_G16_OT4_PREMIX_Plate_Plate01_C03
270 bases / Sep 28, 2023
100 .. 158

5: FOSL2_G17_OT4_PREMIX_Plate_Plate01_D03
269 bases / Sep 28, 2023
99 .. 157

6: FOSL2_A4_OT4_R_PREMIX_B11
270 bases / Sep 29, 2023
100 .. 158

7: FOSL2_A7-1_OT4_PREMIX_Plate_Plate01_G03
271 bases / Sep 28, 2023
99 .. 157

8: FOSL2_A7-2_OT4_PREMIX_Plate_Plate01_H03
272 bases / Sep 28, 2023
100 .. 158

9: FOSL2_A20_OT4_PREMIX_Plate_Plate01_F03
269 bases / Sep 28, 2023
99 .. 157

Sequence: FOSL2_offtarget5_chr7_28766739.print.ver.dna (Linear / 58 bp)
Features: 1 visible, 1 total

10 20 30 40 50

5′ TAACTAAGCCCTAAATAGTGAGGAGGAAGACTCCCTCAGGTGTCCAGCTCAGCCACAT 3′

3′ ATTGATTCGGGATTTATCACTCCTCCTTCTGAGGGAGTCCACAGGTCGAGTCGGTGTA 5′

offtarget

TAACTAAGCCCTAAATAGTGAGGAGGAAGACTCCCTCAGGTGTCCAGCTCAGCCACAT

1 TAACTAAGCCCTAAATAGTGAGGAGGAAGACTCCCTCAGGTGTCCAGCTCAGCCACAT

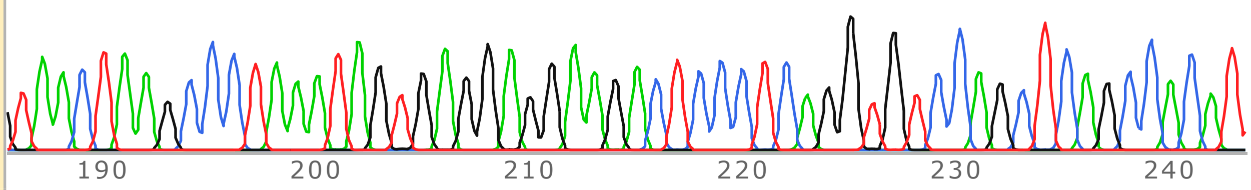

2 TAACTAAGCCCTAAATAGTGAGGAGGAAGACTCCCTCAGGTGTCCAGCTCAGCCACAT

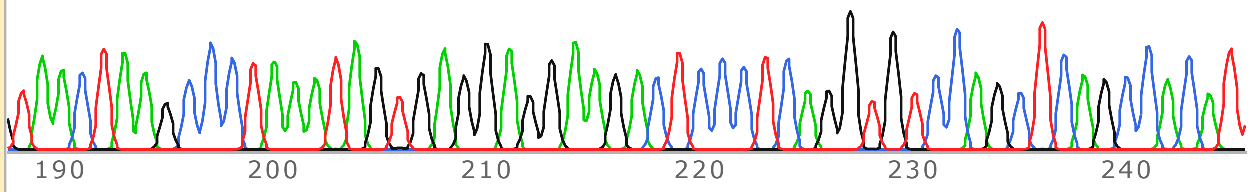

3 TAACTAAGCCCTAAATAGTGAGGAGGAAGACTCCCTCAGGTGTCCAGCTCAGCCACAT

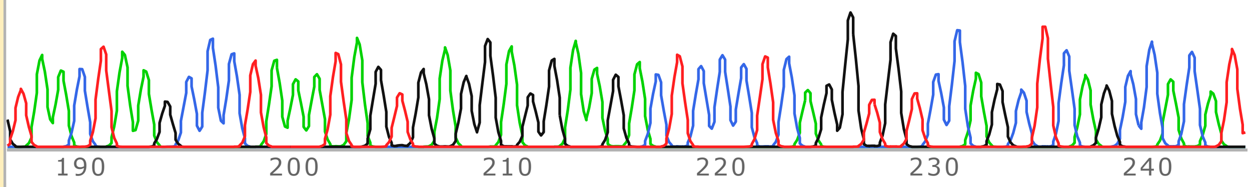

4 TAACTAAGCCCTAAATAGTGAGGAGGAAGACTCCCTCAGGTGTCCAGCTCAGCCACAT

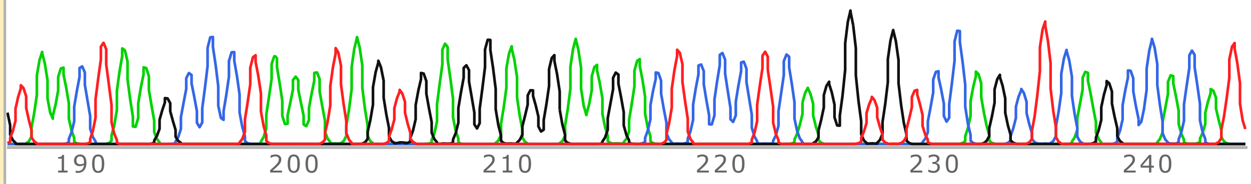

5 TAACTAAGCCCTAAATAGTGAGGAGGAAGACTCCCTCAGGTGTCCAGCTCAGCCACAT

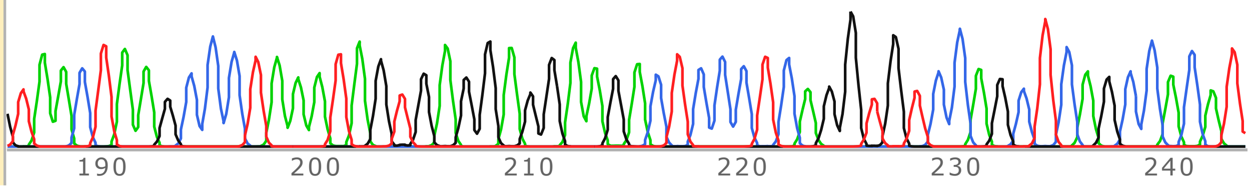

6 TAACTAAGCCCTAAATAGTGAGGAGGAAGACTCCCTCAGGTGTCCAGCTCAGCCACAT

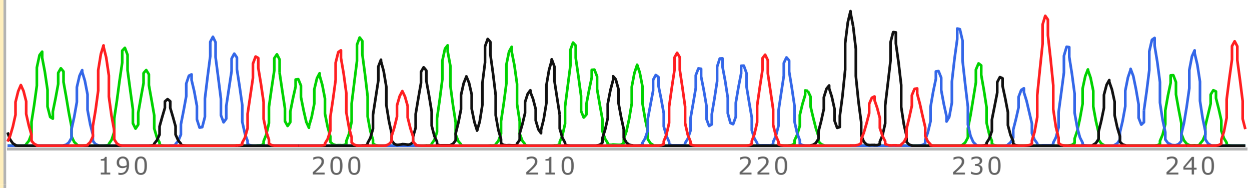

7 TAACTAAGCCCTAAATAGTGAGGAGGAAGACTCCCTCAGGTGTCCAGCTCAGCCACAT

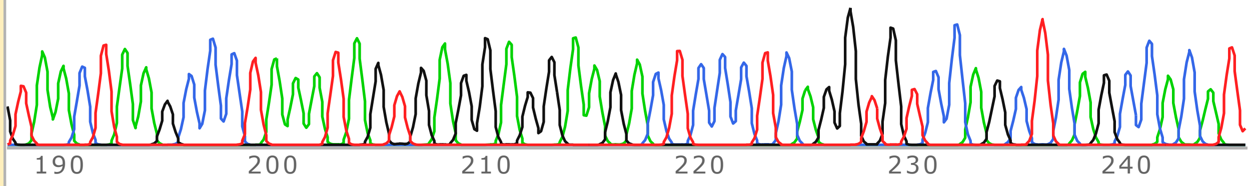

8 TAACTAAGCCCTAAATAGTGAGGAGGAAGACTCCCTCAGGTGTCCAGCTCAGCCACAT

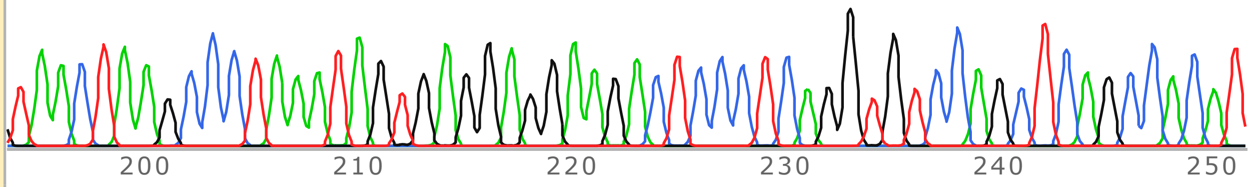

9 TAACTAAGCCCTAAATAGTGAGGAGGAAGACTCCCTCAGGTGTCCAGCTCAGCCACAT

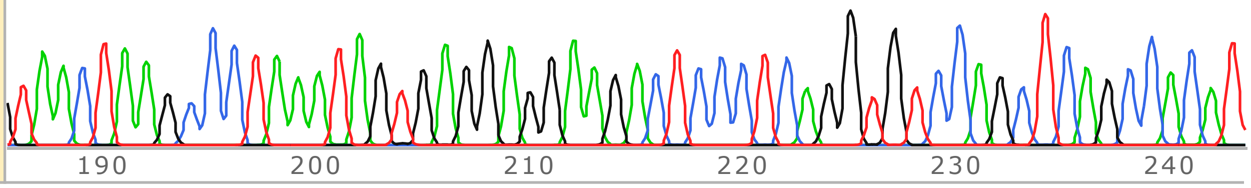

Original Sequence:

1: HEK293T_WT_OT5_PREMIX_Plate_Plate01_E06
453 bases / Sep 23, 2023
186 .. 243

2: FOSL2_G3_OT5_PREMIX_Plate_Plate01_A04
455 bases / Sep 23, 2023
188 .. 245

3: FOSL2_G8_OT5_PREMIX_Plate_Plate01_B04
455 bases / Sep 23, 2023
187 .. 244

4: FOSL2_G16_OT5_PREMIX_Plate_Plate01_C04
455 bases / Sep 23, 2023
187 .. 244

5: FOSL2_G17_OT5_PREMIX_Plate_Plate01_D04
452 bases / Sep 23, 2023
186 .. 243

6: FOSL2_A4_OT5_PREMIX_Plate_Plate01_E04
453 bases / Sep 23, 2023
185 .. 242

7: FOSL2_A7-1_OT5_PREMIX_Plate_Plate01_G04
455 bases / Sep 23, 2023
188 .. 245

8: FOSL2_A7-2_OT5_PREMIX_Plate_Plate01_H04
463 bases / Sep 23, 2023
194 .. 251

9: FOSL2_A20_OT5_PREMIX_Plate_Plate01_F04
453 bases / Sep 23, 2023
186 .. 243

Sequence: FOSL2_offtarget6_chr9_96062501.print.ver.dna (Linear / 58 bp)
Features: 1 visible, 1 total

10 20 30 40 50

5′ CTGTGATCCTGGTAGAAGGGGCAAGGAACACTACCTCAGGAACAAAGGTCACAATGGA 3′

3′ GACACTAGGACCATCTTCCCCGTTCCTTGTGATGGAGTCCTTGTTTCCAGTGTTACCT 5′

offtarget

CTGTGATCCTGGTAGAAGGGGCAAGGAACACTACCTCAGGAACAAAGGTCACAATGGA

1 CTGTGATCCTGGTAGAAGGGGCAAGGAACACTACCTCAGGAACAAAGGTCACAATGGA

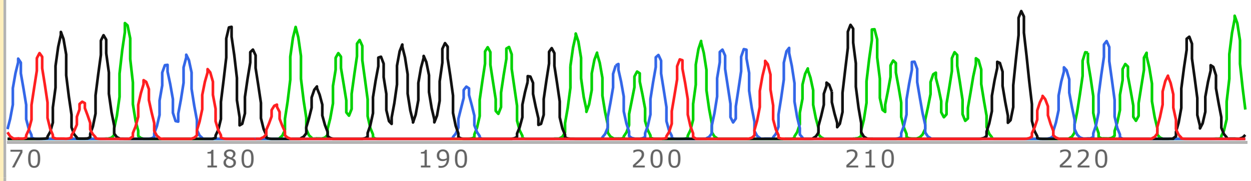

2 CTGTGATCCTGGTAGAAGGGGCAAGGAACACTACCTCAGGAACAAAGGTCACAATGGA

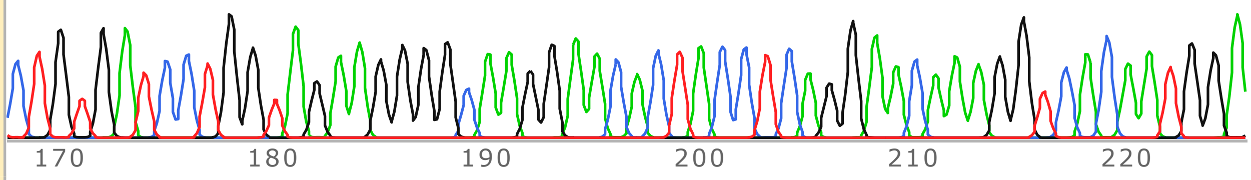

3 CTGTGATCCTGGTAGAAGGGGCAAGGAACACTACCTCAGGAACAAAGGTCACAATGGA

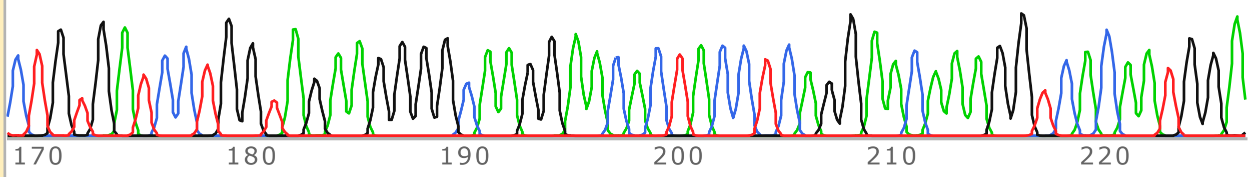

4 CTGTGATCCTGGTAGAAGGGGCAAGGAACACTACCTCAGGAACAAAGGTCACAATGGA

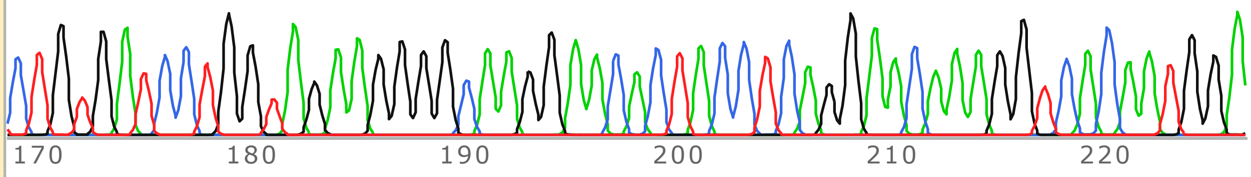

5 CTGTGATCCTGGTAGAAGGGGCAAGGAACACTACCTCAGGAACAAAGGTCACAATGGA

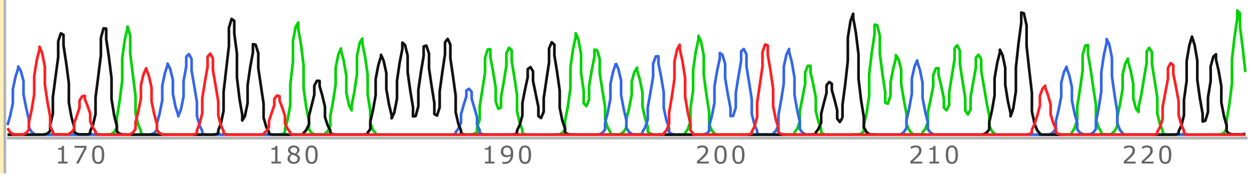

6 CTGTGATCCTGGTAGAAGGGGCAAGGAACACTACCTCAGGAACAAAGGTCACAATGGA

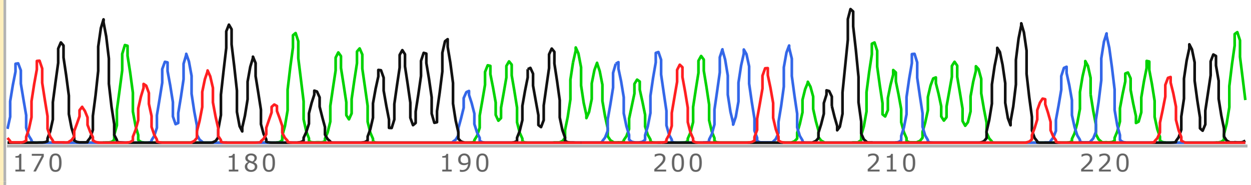

7 CTGTGATCCTGGTAGAAGGGGCAAGGAACACTACCTCAGGAACAAAGGTCACAATGGA

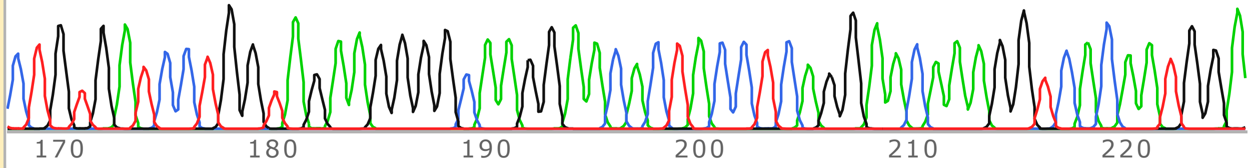

8 CTGTGATCCTGGTAGAAGGGGCAAGGAACACTACCTCAGGAACAAAGGTCACAATGGA

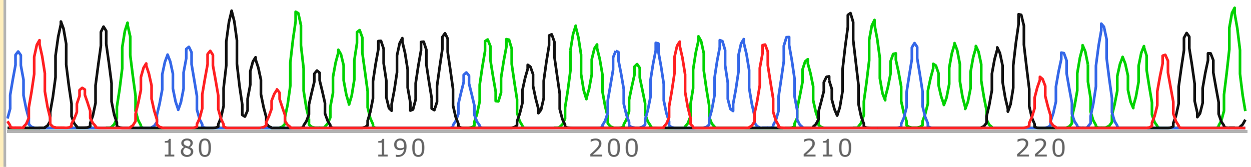

9 CTGTGATCCTGGTAGAAGGGGCAAGGAACACTACCTCAGGAACAAAGGTCACAATGGA

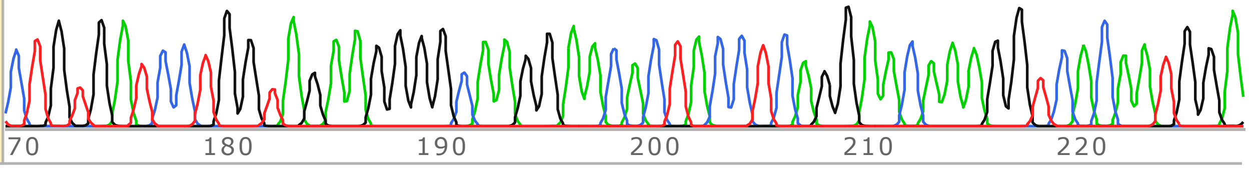

Original Sequence:

1: HEK293T_WT_OT6_PREMIX_Plate_Plate01_F06
459 bases / Sep 23, 2023
170 .. 227

2: FOSL2_G3_OT6_PREMIX_Plate_Plate01_A05
458 bases / Sep 23, 2023
168 .. 225

3: FOSL2_G8_OT6_PREMIX_Plate_Plate01_B05
460 bases / Sep 23, 2023
169 .. 226

4: FOSL2_G16_OT6_PREMIX_Plate_Plate01_C05
455 bases / Sep 23, 2023
169 .. 226

5: FOSL2_G17_OT6_PREMIX_Plate_Plate01_D05
457 bases / Sep 23, 2023
167 .. 224

6: FOSL2_A4_OT6_PREMIX_Plate_Plate01_E05
457 bases / Sep 23, 2023
169 .. 226

7: FOSL2_A7-1_OT6_PREMIX_Plate_Plate01_G05
456 bases / Sep 23, 2023
168 .. 225

8: FOSL2_A7-2_OT6_PREMIX_Plate_Plate01_H05
461 bases / Sep 23, 2023
172 .. 229

9: FOSL2_A20_OT6_PREMIX_Plate_Plate01_F05
457 bases / Sep 23, 2023
170 .. 227

Sequence: IRAK1_offtarget1_chr8_143586472.print.ver.dna (Linear / 58 bp)
Features: 1 visible, 1 total

10 20 30 40 50

5′ GTGGGGCCAGGCCTGGCACTCCTTCCCCCGCAGACATGGGCGCCTTTGCCTGTCGTCA 3′

3′ CACCCCGGTCCGGACCGTGAGGAAGGGGGCGTCTGTACCCGCGGAAACGGACAGCAGT 5′

offtarget

GTGGGGCCAGGCCTGGCACTCCTTCCCCCGCAGACATGGGCGCCTTTGCCTGTCGTCA

1 GTGGGGCCAGGCCTGGCACTCCTTCCCCCGCAGACATGGGCGCCTTTGCCTGTCGTCA

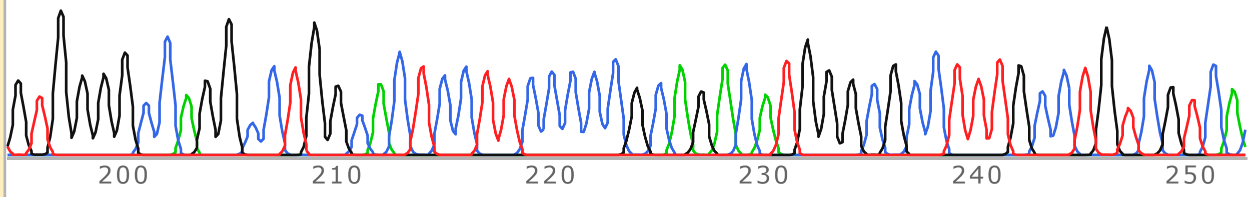

2 GTGGGGCCAGGCCTGGCACTCCTTCCCCCGCAGACATGGGCGCCTTTGCCTGTCGTCA

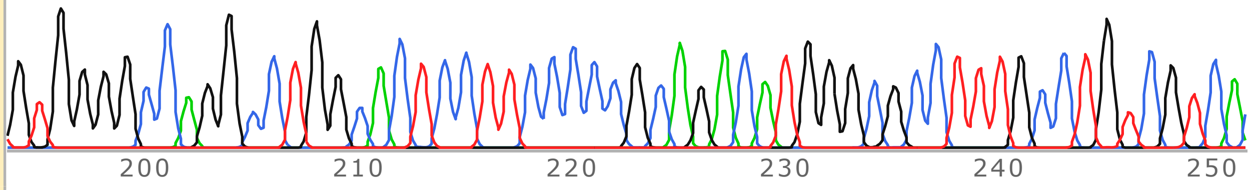

3 GTGGGGCCAGGCCTGGCACTCCTTCCCCCGCAGACATGGGCGCCTTTGCCTGTCGTCA

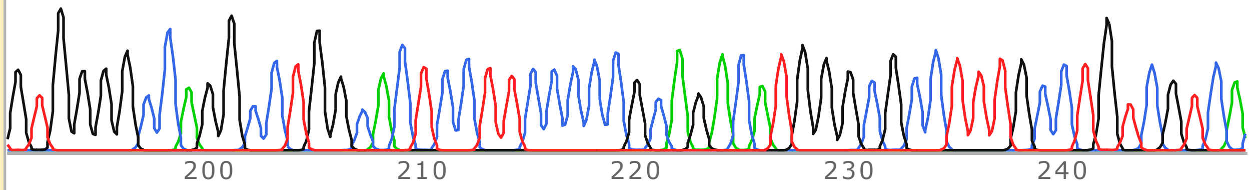

4 GTGGGGCCAGGCCTGGCACTCCTTCCCCCGCAGACATGGGCGCCTTTGCCTGTCGTCA

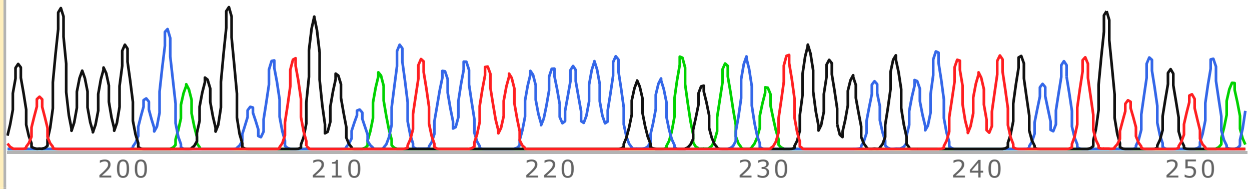

5 GTGGGGCCAGGCCTGGCACTCCTTCCCCCGCAGACATGGGCGCCTTTGCCTGTCGTCA

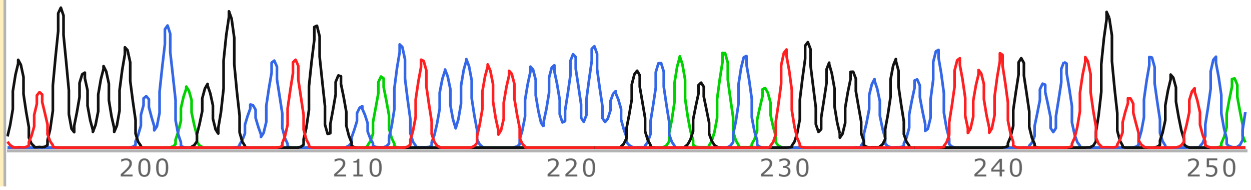

6 GTGGGGCCAGGCCTGGCACTCCTTCCCCCGCAGACATGGGCGCCTTTGCCTGTCGTCA

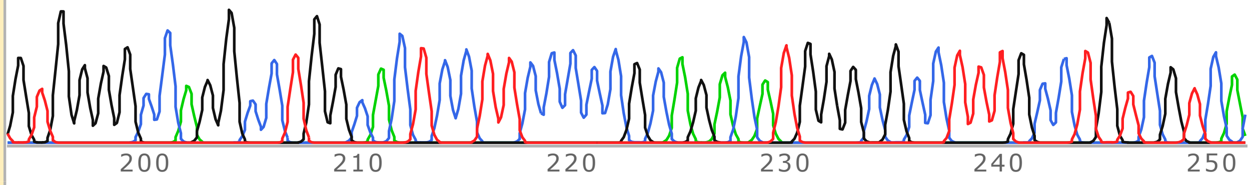

7 GTGGGGCCAGGCCTGGCACTCCTTCCCCCGCAGACATGGGCGCCTTTGCCTGTCGTCA

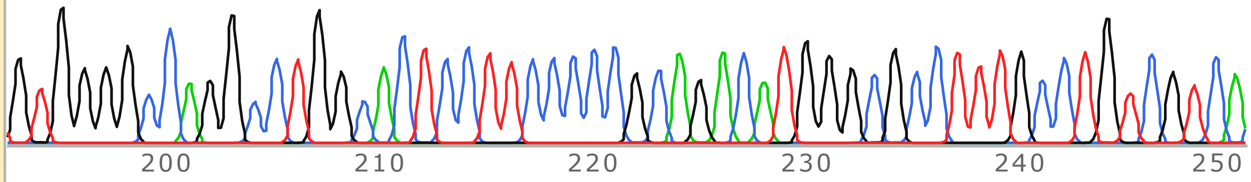

8 GTGGGGCCAGGCCTGGCACTCCTTCCCCCGCAGACATGGGCGCCTTTGCCTGTCGTCA

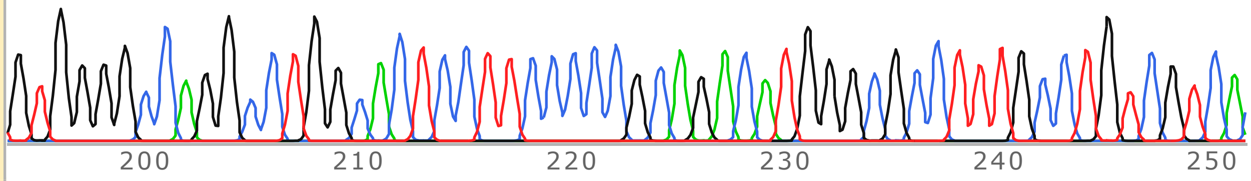

9 GTGGGGCCAGGCCTGGCACTCCTTCCCCCGCAGACATGGGCGCCTTTGCCTGTCGTCA

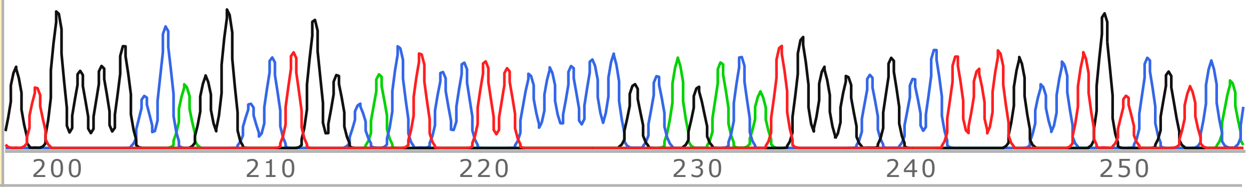

Original Sequence:

1: HEK293T_WT_OT1_PREMIX_Plate_Plate01_A07
456 bases / Sep 27, 2023
195 .. 252

2: IRAK1_G9_OT1_PREMIX_Plate_Plate01_A01
457 bases / Sep 27, 2023
194 .. 251

3: IRAK1_G9-2_OT1_PREMIX_Plate_Plate01_C01
453 bases / Sep 27, 2023
191 .. 248

4: IRAK1_G9-3_OT1_PREMIX_Plate_Plate01_D01
456 bases / Sep 27, 2023
195 .. 252

5: IRAK1_G10_OT1_PREMIX_Plate_Plate01_B01
455 bases / Sep 27, 2023
194 .. 251

6: IRAK1_A8-2_OT1_PREMIX_Plate_Plate01_E01
456 bases / Sep 27, 2023
194 .. 251

7: IRAK1_A8-3-2_OT1_PREMIX_Plate_Plate01_F01
454 bases / Sep 27, 2023
193 .. 250

8: IRAK1_A8-3-4_OT1_PREMIX_Plate_Plate01_G01
457 bases / Sep 27, 2023
194 .. 251

9: IRAK1_A8-3-5_OT1_F_PREMIX_A12
461 bases / Oct 4, 2023
198 .. 255

Sequence: IRAK1_offtarget2_chr11_80157991.print.ver.dna (Linear / 60 bp)
Features: 1 visible, 1 total

10 20 30 40 50 60

5′ CTCTGCTCAATGCAGCTGCTTTCTTTCCTCCACAGGCATTGGCCCTAAAGGCCCCTTTCA 3′

3′ GAGACGAGTTACGTCGACGAAAGAAAGGAGGTGTCCGTAACCGGGATTTCCGGGGAAAGT 5′

offtarget

CTCTGCTCAATGCAGCTGCTTTCTTTCCTCCACAGGCATTGGCCCTAAAGGCCCCTTTCA

1 CTCTGCTCAATGCAGCTGCTTTCTTTCCTCCACAGGCATTGGCCCTAAAGGCCCCTTTCA

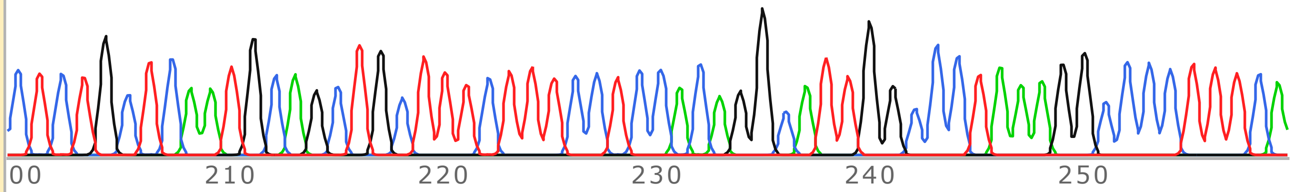

2 CTCTGCTCAATGCAGCTGCTTTCTTTCCTCCACAGGCATTGGCCCTAAAGGCCCCTTTCA

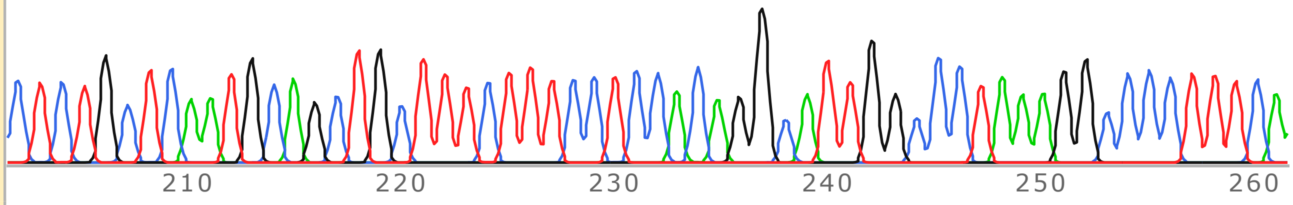

3 CTCTGCTCAATGCAGCTGCTTTCTTTCCTCCACAGGCATTGGCCCTAAAGGCCCCTTTCA

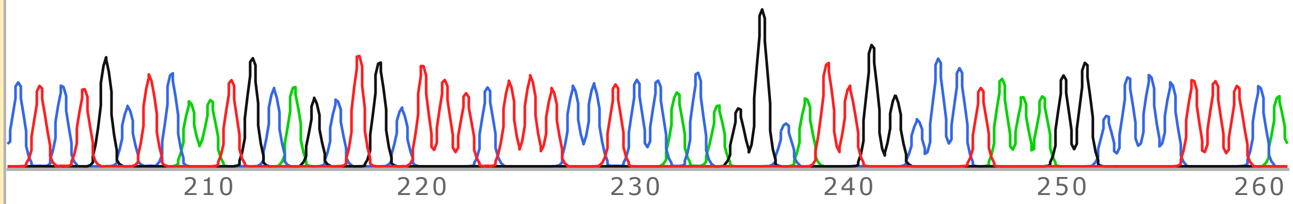

4 CTCTGCTCAATGCAGCTGCTTTCTTTCCTCCACAGGCATTGGCCCTAAAGGCCCCTTTCA

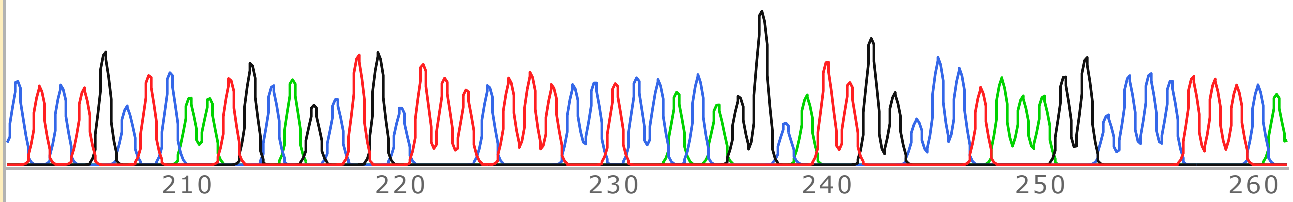

5 CTCTGCTCAATGCAGCTGCTTTCTTTCCTCCACAGGCATTGGCCCTAAAGGCCCCTTTCA

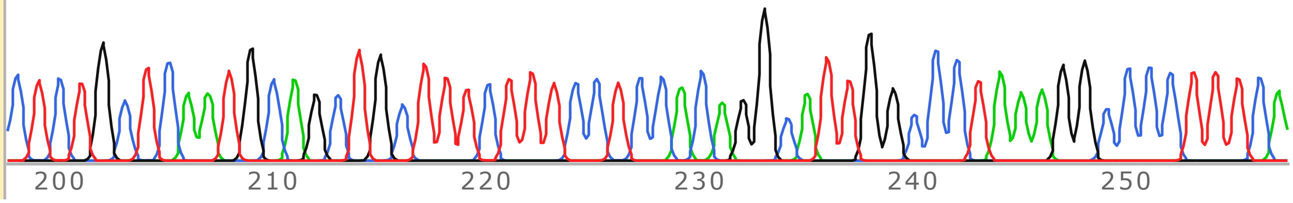

6 CTCTGCTCAATGCAGCTGCTTTCTTTCCTCCACAGGCATTGGCCCTAAAGGCCCCTTTCA

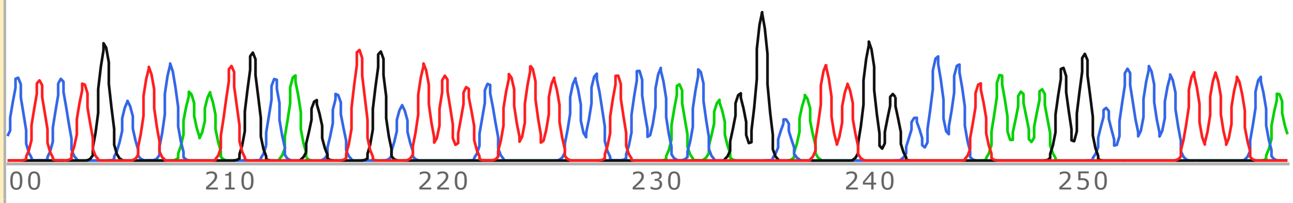

7 CTCTGCTCAATGCAGCTGCTTTCTTTCCTCCACAGGCATTGGCCCTAAAGGCCCCTTTCA

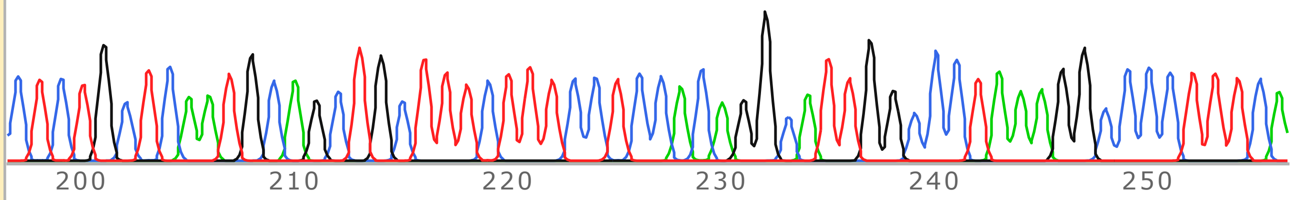

8 CTCTGCTCAATGCAGCTGCTTTCTTTCCTCCACAGGCATTGGCCCTAAAGGCCCCTTTCA

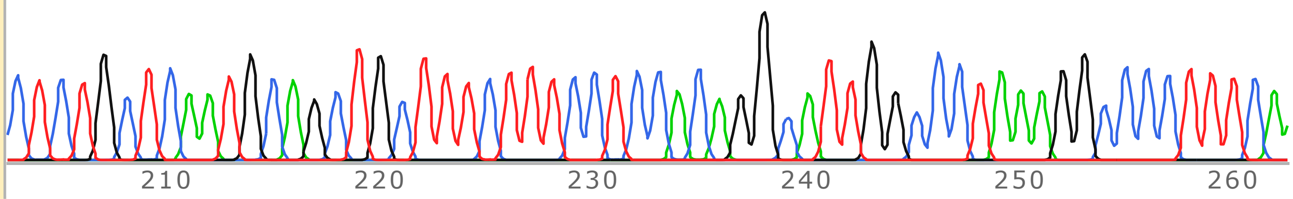

9 CTCTGCTCAATGCAGCTGCTTTCTTTCCTCCACAGGCATTGGCCCTAAAGGCCCCTTTCA

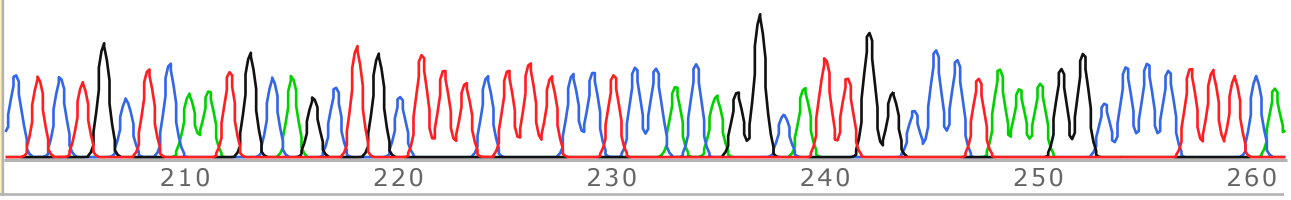

Original Sequence:

1: HEK293T_WT_OT2_PREMIX_Plate_Plate01_B07
467 bases / Sep 27, 2023
200 .. 259

2: IRAK1_G9_OT2_PREMIX_Plate_Plate01_A02
469 bases / Sep 27, 2023
202 .. 261

3: IRAK1_G9-2_OT2_PREMIX_Plate_Plate01_C02
468 bases / Sep 27, 2023
201 .. 260

4: IRAK1_G9-3_OT2_PREMIX_Plate_Plate01_D02
470 bases / Sep 27, 2023
202 .. 261

5: IRAK1_G10_OT2_PREMIX_Plate_Plate01_B02
465 bases / Sep 27, 2023
198 .. 257

6: IRAK1_A8-2_OT2_PREMIX_Plate_Plate01_E02
467 bases / Sep 27, 2023
200 .. 259

7: IRAK1_A8-3-2_OT2_PREMIX_Plate_Plate01_F02
464 bases / Sep 27, 2023
197 .. 256

8: IRAK1_A8-3-4_OT2_PREMIX_Plate_Plate01_G02
470 bases / Sep 27, 2023
203 .. 262

9: IRAK1_A8-3-5_OT2_PREMIX_Plate_Plate01_H02
468 bases / Sep 27, 2023
202 .. 261

Sequence: IRAK1_offtarget3_chr1_158091341.print.ver.dna (Linear / 58 bp)
Features: 1 visible, 1 total

10 20 30 40 50

5′ TTTCTTACCTTCTTCCTTCCCTCTCCACCACAGGCATGGGCCTGGAAGGAGAACTTCT 3′

3′ AAAGAATGGAAGAAGGAAGGGAGAGGTGGTGTCCGTACCCGGACCTTCCTCTTGAAGA 5′

offtarget

TTTCTTACCTTCTTCCTTCCCTCTCCACCACAGGCATGGGCCTGGAAGGAGAACTTCT

1 TTTCTTACCTTCTTCCTTCCCTCTCCACCACAGGCATGGGCCTGGAAGGAGAACTTCT

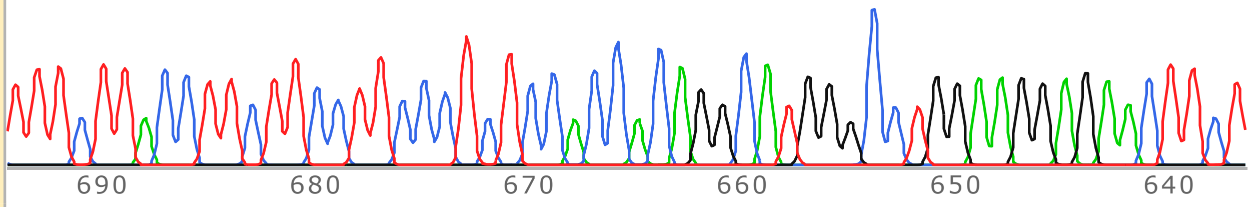

2 TTTCTTACCTTCTTCCTTCCCTCTCCACCACAGGCATGGGCCTGGAAGGAGAACTTCT

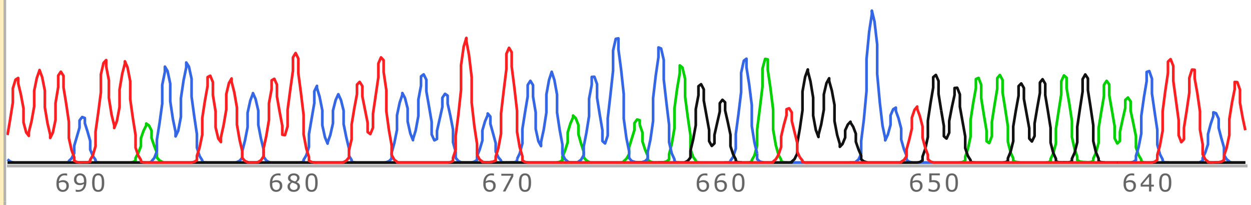

3 TTTCTTACCTTCTTCCTTCCCTCTCCACCACAGGCATGGGCCTGGAAGGAGAACTTCT

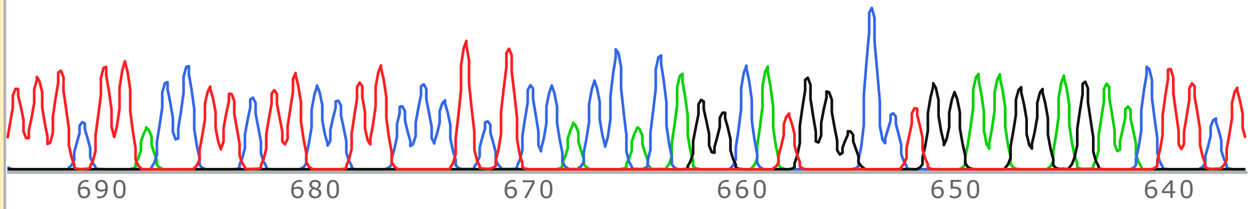

4 TTTCTTACCTTCTTCCTTCCCTCTCCACCACAGGCATGGGCCTGGAAGGAGAACTTCT

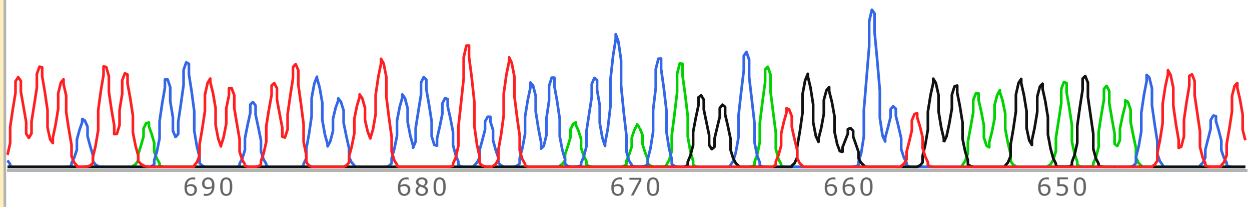

5 TTTCTTACCTTCTTCCTTCCCTCTCCACCACAGGCATGGGCCTGGAAGGAGAACTTCT

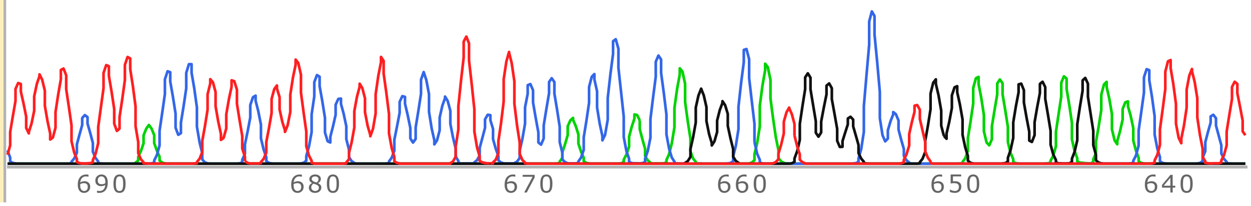

6 ← TTTCTTACCTTCTTCCTTCCCTCTCCACCACAGGCATGGGCCTGGAAGGAGAACTTCT

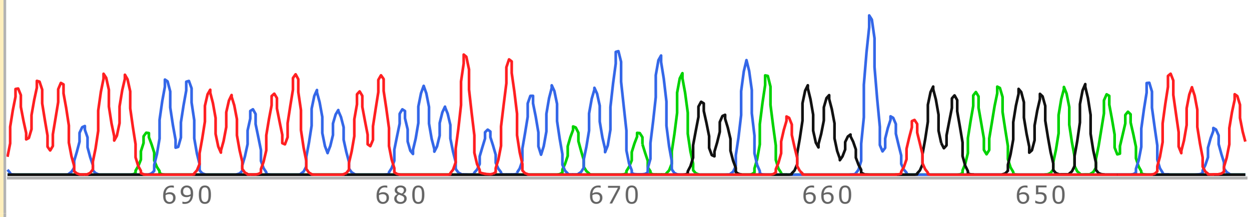

7 ← TTTCTTACCTTCTTCCTTCCCTCTCCACCACAGGCATGGGCCTGGAAGGAGAACTTCT

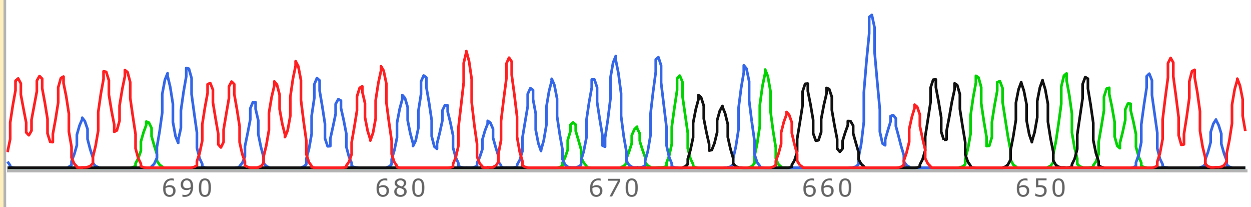

8 ← TTTCTTACCTTCTTCCTTCCCTCTCCACCACAGGCATGGGCCTGGAAGGAGAACTTCT

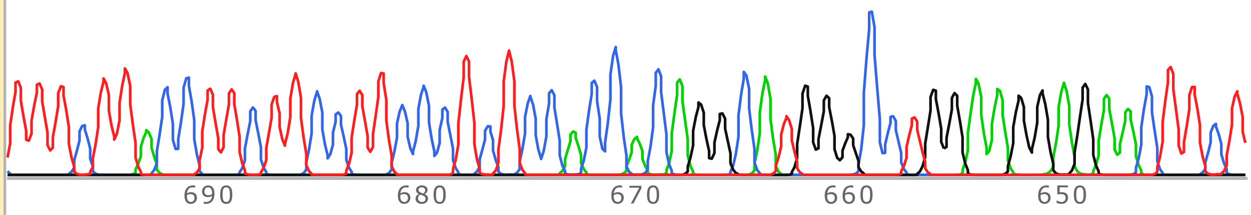

9 ← TTTCTTACCTTCTTCCTTCCCTCTCCACCACAGGCATGGGCCTGGAAGGAGAACTTCT

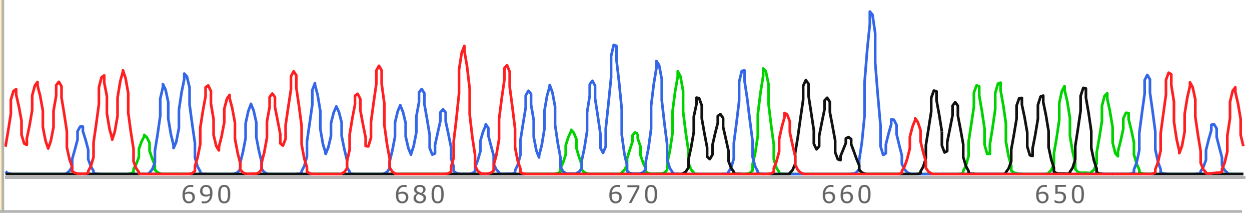

Original Sequence:

1: HEK293T_WT_OT3_PREMIX_Plate_Plate01_D05
1101 bases / Sep 28, 2023
637 .. 694

2: IRAK1_G9_OT3_PREMIX_Plate_Plate01_A04
1110 bases / Sep 28, 2023
636 .. 693

3: IRAK1_G9-2_OT3_PREMIX_Plate_Plate01_C04
1132 bases / Sep 28, 2023
637 .. 694

4: IRAK1_G9-3_OT3_PREMIX_Plate_Plate01_D04
1148 bases / Sep 28, 2023
642 .. 699

5: IRAK1_G10_OT3_PREMIX_Plate_Plate01_B04
1116 bases / Sep 28, 2023
637 .. 694

6: IRAK1_A8-2_OT3_PREMIX_Plate_Plate01_E04
1146 bases / Sep 28, 2023
641 .. 698

7: IRAK1_A8-3-2_OT3_PREMIX_Plate_Plate01_F04
1128 bases / Sep 28, 2023
641 .. 698

8: IRAK1_A8-3-4_OT3_PREMIX_Plate_Plate01_G04
1128 bases / Sep 28, 2023
642 .. 699

9: IRAK1_A8-3-5_OT3_PREMIX_Plate_Plate01_H04
1136 bases / Sep 28, 2023
642 .. 699

Sequence: IRAK1_offtarget4_chr8_29664635.print.ver.dna (Linear / 60 bp)
Features: 1 visible, 1 total

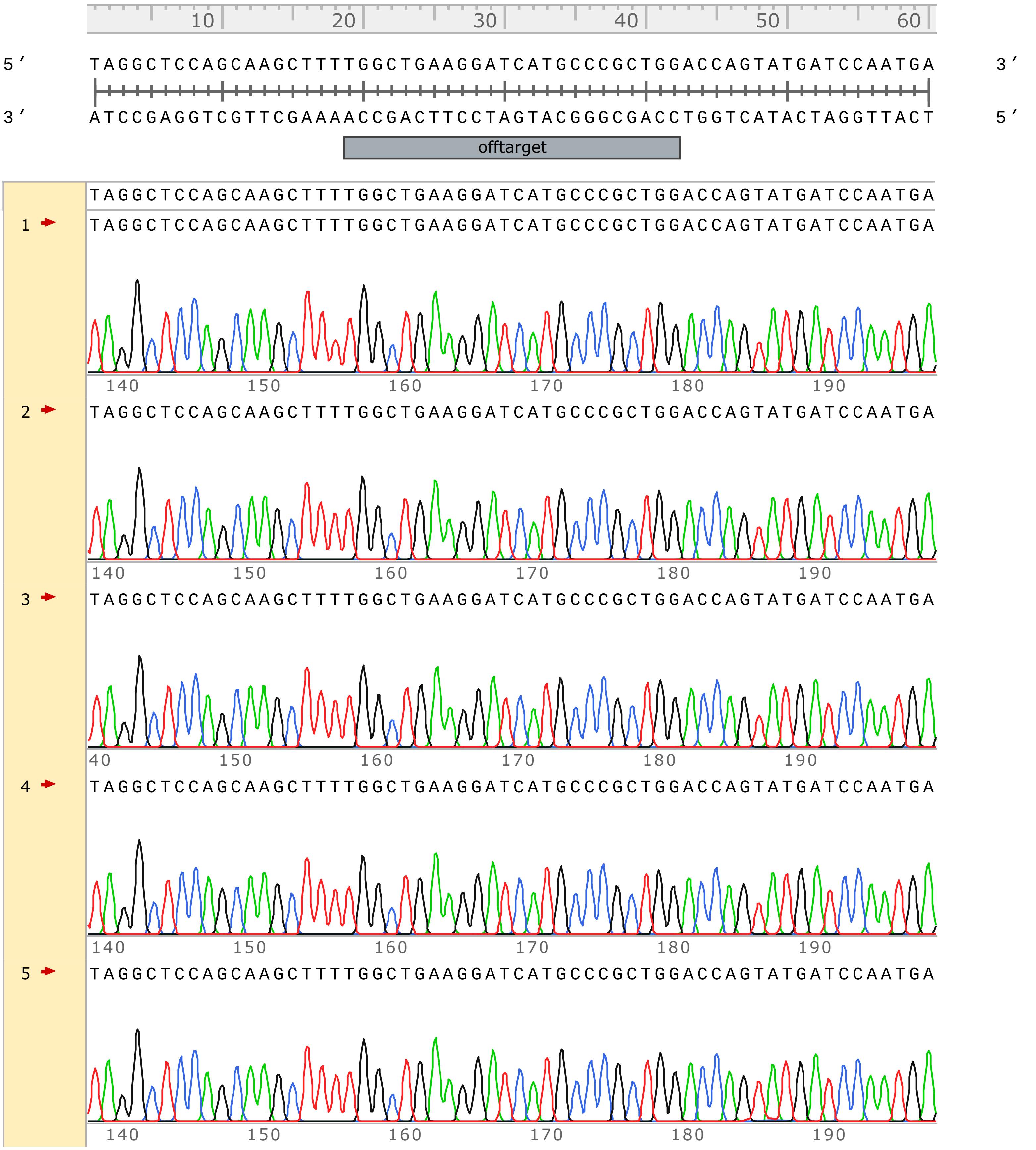

6 TAGGCTCCAGCAAGCTTTTGGCTGAAGGATCATGCCCGCTGGACCAGTATGATCCAATGA

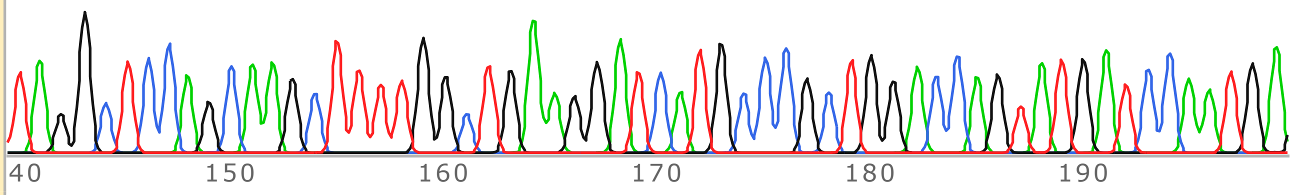

7 TAGGCTCCAGCAAGCTTTTGGCTGAAGGATCATGCCCGCTGGACCAGTATGATCCAATGA

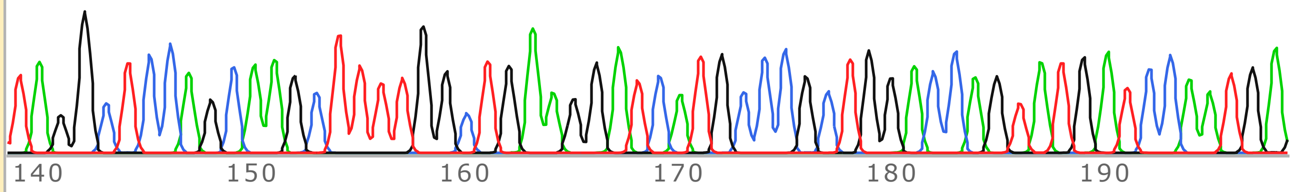

8 TAGGCTCCAGCAAGCTTTTGGCTGAAGGATCATGCCCGCTGGACCAGTATGATCCAATGA

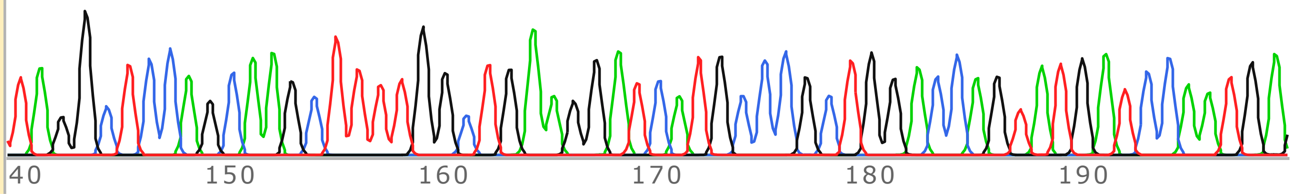

9 TAGGCTCCAGCAAGCTTTTGGCTGAAGGATCATGCCCGCTGGACCAGTATGATCCAATGA

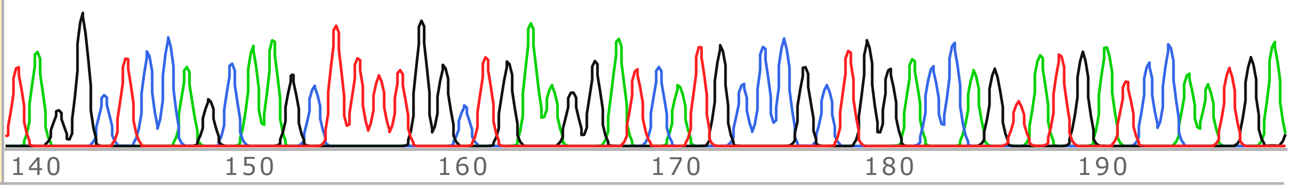

Original Sequence:

1: HEK293T_WT_OT4_PREMIX_Plate_Plate01_D07
433 bases / Sep 27, 2023
138 .. 197

2: IRAK1_G9_OT4_PREMIX_Plate_Plate01_A04
430 bases / Sep 27, 2023
139 .. 198

3: IRAK1_G9-2_OT4_PREMIX_Plate_Plate01_C04
434 bases / Sep 27, 2023
140 .. 199

4: IRAK1_G9-3_OT4_PREMIX_Plate_Plate01_D04
433 bases / Sep 27, 2023
139 .. 198

5: IRAK1_G10_OT4_PREMIX_Plate_Plate01_B04
430 bases / Sep 27, 2023
138 .. 197

6: IRAK1_A8-2_OT4_PREMIX_Plate_Plate01_E04
433 bases / Sep 27, 2023
140 .. 199

7: IRAK1_A8-3-2_OT4_PREMIX_Plate_Plate01_F04
432 bases / Sep 27, 2023
139 .. 198

8: IRAK1_A8-3-4_OT4_PREMIX_Plate_Plate01_G04
432 bases / Sep 27, 2023
140 .. 199

9: IRAK1_A8-3-5_OT4_PREMIX_Plate_Plate01_H04
433 bases / Sep 27, 2023
139 .. 198

Sequence: IRAK1_offtarget5_chr10_77375945.print.ver.dna (Linear / 60 bp)
Features: 1 visible, 1 total

10 20 30 40 50 60

5′ TCTGACACGAAGTTGAGTGTGCTGGAGGCTCATACCCACGGGAGGAGGGGAAGGCAGCAG 3′

3′ AGACTGTGCTTCAACTCACACGACCTCCGAGTATGGGTGCCCTCCTCCCCTTCCGTCGTC 5′

offtarget

TCTGACACGAAGTTGAGTGTGCTGGAGGCTCATACCCACGGGAGGAGGGGAAGGCAGCAG

1 TCTGACACGAAGTTGAGTGTGCTGGAGGCTCATACCCACGGGAGGAGGGGAAGGCAGCAG

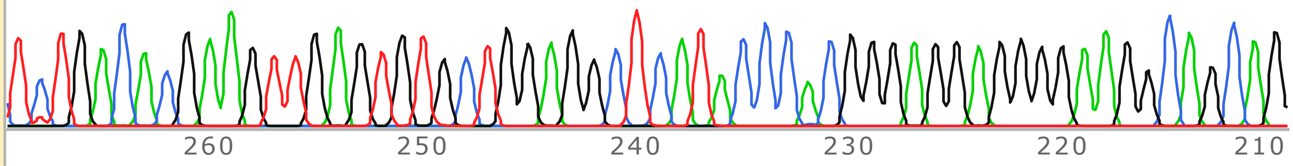

2 TCTGACACGAAGTTGAGTGTGCTGGAGGCTCATACCCACGGGAGGAGGGGAAGGCAGCAG

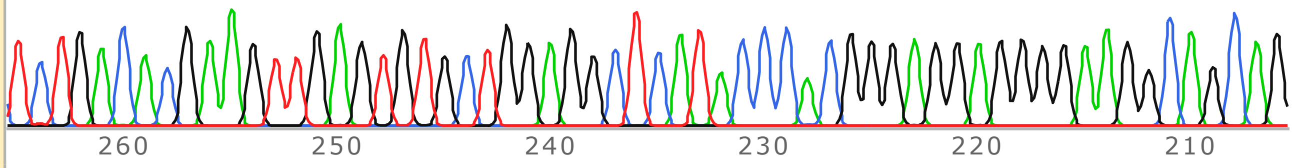

3 TCTGACACGAAGTTGAGTGTGCTGGAGGCTCATACCCACGGGAGGAGGGGAAGGCAGCAG

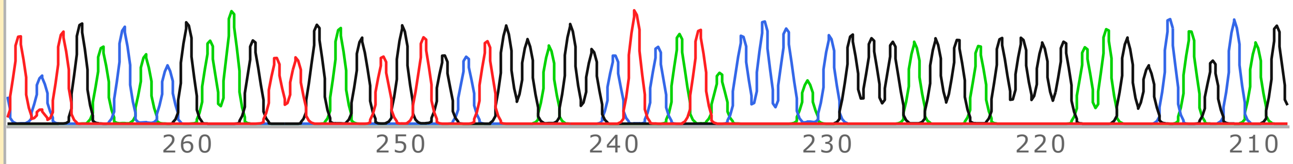

4 TCTGACACGAAGTTGAGTGTGCTGGAGGCTCATACCCACGGGAGGAGGGGAAGGCAGCAG

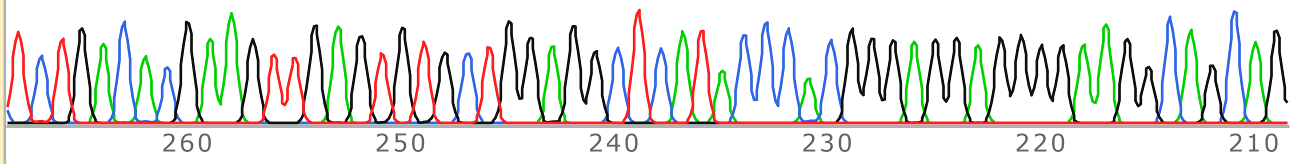

5 TCTGACACGAAGTTGAGTGTGCTGGAGGCTCATACCCACGGGAGGAGGGGAAGGCAGCAG

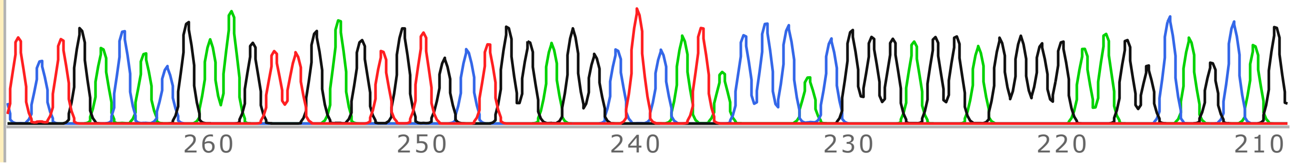

6 TCTGACACGAAGTTGAGTGTGCTGGAGGCTCATACCCACGGGAGGAGGGGAAGGCAGCAG

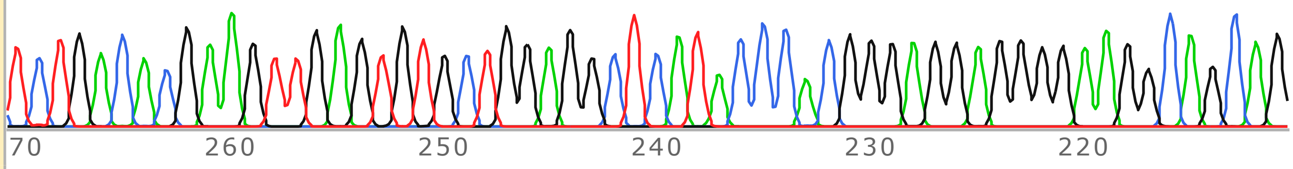

7 TCTGACACGAAGTTGAGTGTGCTGGAGGCTCATACCCACGGGAGGAGGGGAAGGCAGCAG

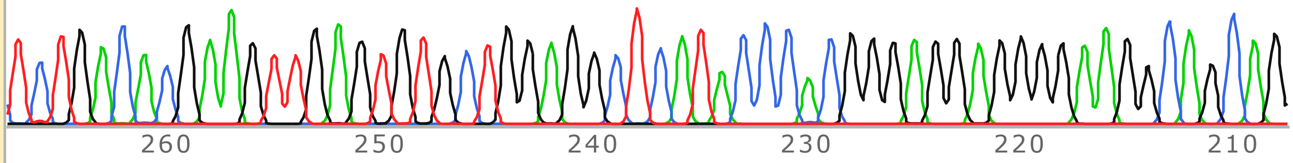

8 TCTGACACGAAGTTGAGTGTGCTGGAGGCTCATACCCACGGGAGGAGGGGAAGGCAGCAG

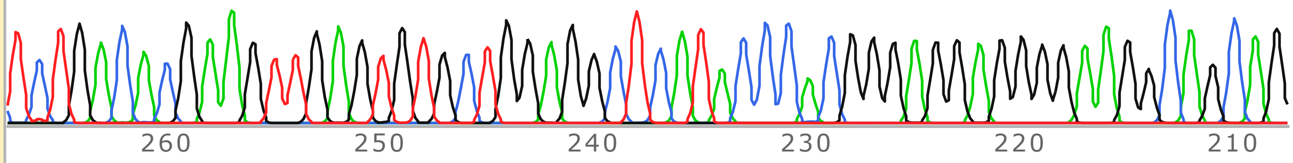

9 TCTGACACGAAGTTGAGTGTGCTGGAGGCTCATACCCACGGGAGGAGGGGAAGGCAGCAG

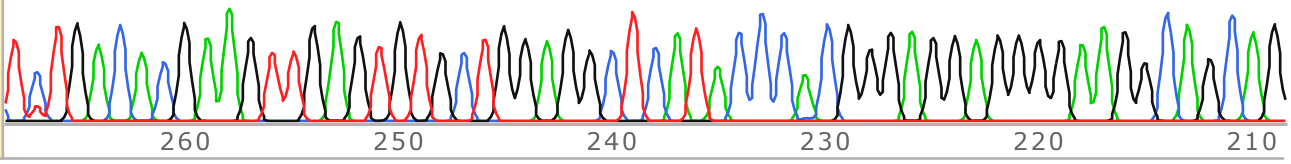

Original Sequence:

1: HEK293T_WT_OT5_PREMIX_Plate_Plate01_E07
477 bases / Sep 27, 2023
210 .. 269

2: IRAK1_G9_OT5_PREMIX_Plate_Plate01_A05
475 bases / Sep 27, 2023
206 .. 265

3: IRAK1_G9-2_OT5_PREMIX_Plate_Plate01_C05
478 bases / Sep 27, 2023
209 .. 268

4: IRAK1_G9-3_OT5_PREMIX_Plate_Plate01_D05
475 bases / Sep 27, 2023
209 .. 268

5: IRAK1_G10_OT5_PREMIX_Plate_Plate01_B05
477 bases / Sep 27, 2023
210 .. 269

6: IRAK1_A8-2_OT5_PREMIX_Plate_Plate01_E05
479 bases / Sep 27, 2023
211 .. 270

7: IRAK1_A8-3-2_OT5_PREMIX_Plate_Plate01_F05
475 bases / Sep 27, 2023
208 .. 267

8: IRAK1_A8-3-4_OT5_PREMIX_Plate_Plate01_G05
475 bases / Sep 27, 2023
208 .. 267

9: IRAK1_A8-3-5_OT5_PREMIX_Plate_Plate01_H05
477 bases / Sep 27, 2023
209 .. 268

Sequence: IRAK1_offtarget6_chr12_132538552.print.ver.dna (Linear / 60 bp)
Features: 1 visible, 1 total

10 20 30 40 50 60

5′ CGCCTGCGAGGGTGCCTCCCGGTGGGGGCTCAAGCCCTCAGGAGAGGCTGTGCCACGTGG 3′

3′ GCGGACGCTCCCACGGAGGGCCACCCCCGAGTTCGGGAGTCCTCTCCGACACGGTGCACC 5′

offtarget

CGCCTGCGAGGGTGCCTCCCGGTGGGGGCTCAAGCCCTCAGGAGAGGCTGTGCCACGTGG

1 ← CGCCTGCGAGGGTGCCTCCCGGTGGGGGCTCAAGCCCTCAGGAGAGGCTGTGCCACGTGG

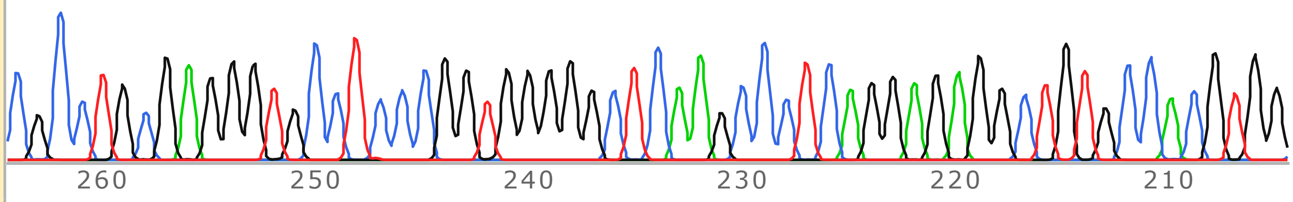

2 ← CGCCTGCGAGGGTGCCTCCCGGTGGGGGCTCAAGCCCTCAGGAGAGGCTGTGCCACGTGG

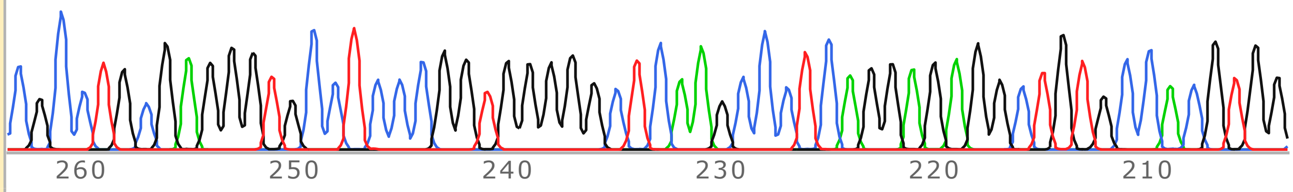

3 ← CGCCTGCGAGGGTGCCTCCCGGTGGGGGCTCAAGCCCTCAGGAGAGGCTGTGCCACGTGG

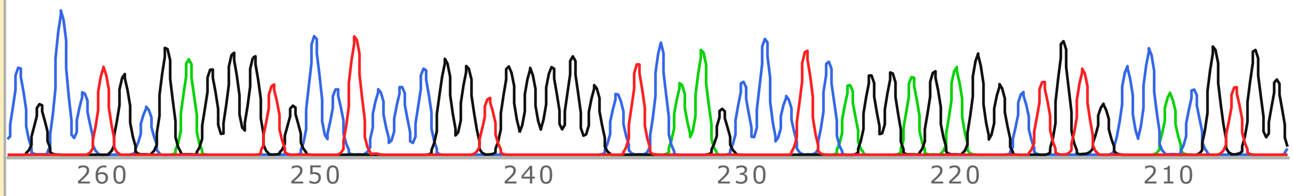

4 ← CGCCTGCGAGGGTGCCTCCCGGTGGGGGCTCAAGCCCTCAGGAGAGGCTGTGCCACGTGG

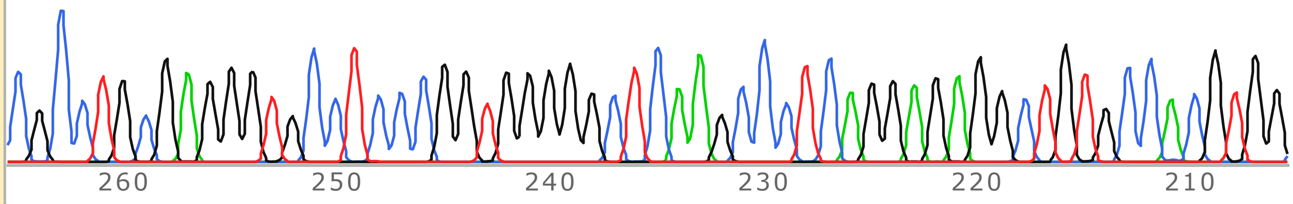

5 ← CGCCTGCGAGGGTGCCTCCCGGTGGGGGCTCAAGCCCTCAGGAGAGGCTGTGCCACGTGG

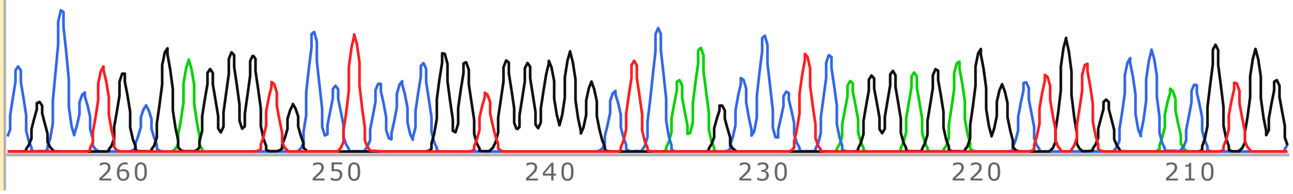

6 ← CGCCTGCGAGGGTGCCTCCCGGTGGGGGCTCAAGCCCTCAGGAGAGGCTGTGCCACGTGG

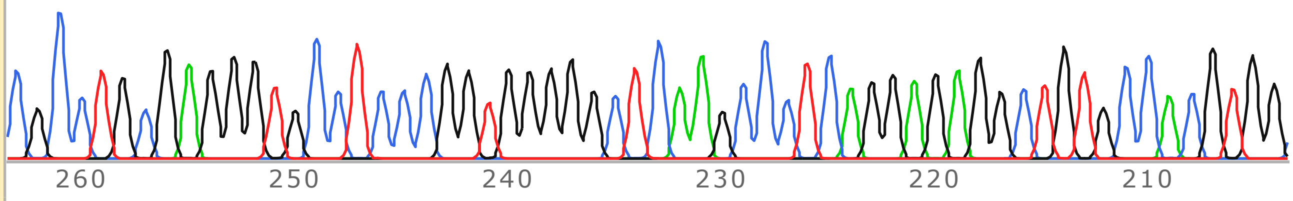

7 ← CGCCTGCGAGGGTGCCTCCCGGTGGGGGCTCAAGCCCTCAGGAGAGGCTGTGCCACGTGG

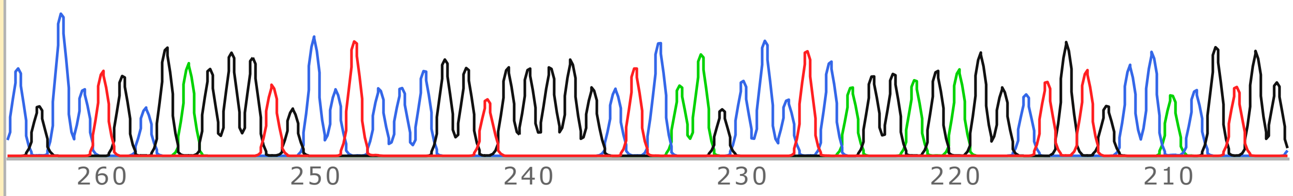

8 ← CGCCTGCGAGGGTGCCTCCCGGTGGGGGCTCAAGCCCTCAGGAGAGGCTGTGCCACGTGG

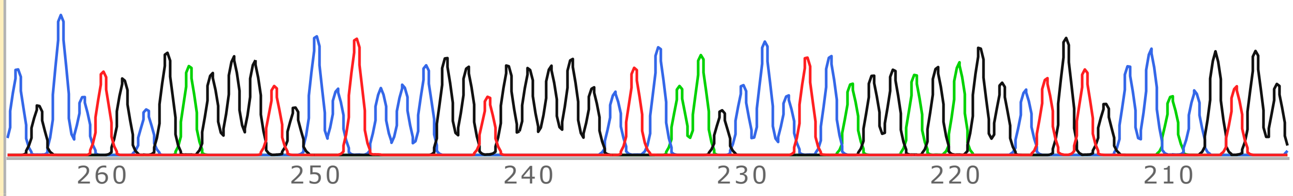

9 ← CGCCTGCGAGGGTGCCTCCCGGTGGGGGCTCAAGCCCTCAGGAGAGGCTGTGCCACGTGG

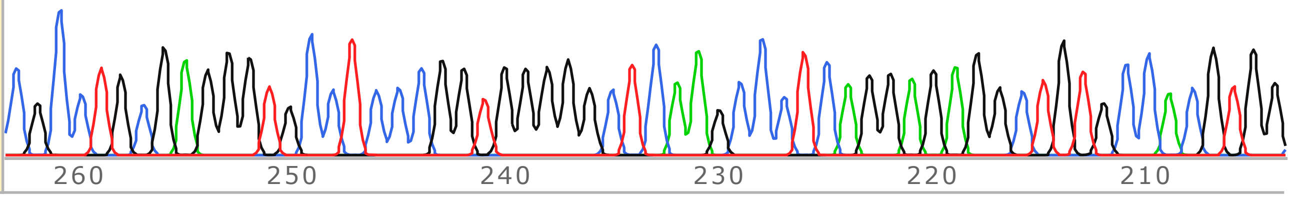

Original Sequence:

1: HEK293T_WT_OT6_PREMIX_Plate_Plate01_F07
452 bases / Sep 27, 2023
205 .. 264

2: IRAK1_G9_OT6_PREMIX_Plate_Plate01_A06
454 bases / Sep 27, 2023
204 .. 263

3: IRAK1_G9-2_OT6_PREMIX_Plate_Plate01_C06
455 bases / Sep 27, 2023
205 .. 264

4: IRAK1_G9-3_OT6_PREMIX_Plate_Plate01_D06
455 bases / Sep 27, 2023
206 .. 265

5: IRAK1_G10_OT6_PREMIX_Plate_Plate01_B06
454 bases / Sep 27, 2023
206 .. 265

6: IRAK1_A8-2_OT6_PREMIX_Plate_Plate01_E06
450 bases / Sep 27, 2023
204 .. 263

7: IRAK1_A8-3-2_OT6_PREMIX_Plate_Plate01_F06
453 bases / Sep 27, 2023
205 .. 264

8: IRAK1_A8-3-4_OT6_PREMIX_Plate_Plate01_G06
452 bases / Sep 27, 2023
205 .. 264

9: IRAK1_A8-3-5_OT6_PREMIX_Plate_Plate01_H06
452 bases / Sep 27, 2023
204 .. 263

Supplementary Note 2. Plasmid map

AseI

5′ TAGTTATTAATAGTAATCAATTACGGGGTCATTAGTTCATAGCCCATATATGGAGTTCCGCGTTA 65
3′ ATCAATAATTATCATTAGTTAATGCCCCAGTAATCAAGTATCGGGTATATACCTCAAGGCGCAAT

CMV enhancer

CATAACTTACGGTAAATGGCCCGCCTGGCTGACCGCCCAACGACCCCCGCCCATTGACGTCAATA 130
GTATTGAATGCCATTTACCGGGCGGACCGACTGGCGGGTTGCTGGGGGCGGGTAACTGCAGTTAT

CMV enhancer

ATGACGTATGTTCCCATAGTAACGCCAATAGGGACTTTCCATTGACGTCAATGGGTGGAGTATTT 195
TACTGCATACAAGGGTATCATTGCGGTTATCCCTGAAAGGTAACTGCAGTTACCCACCTCATAAA

CMV enhancer

NdeI

ACGGTAAACTGCCCACTTGGCAGTACATCAAGTGTATCAtatgccaagtacgccccctattgacg 260
TGCCATTTGACGGGTGAACCGTCATGTAGTTCACATAGTatacggttcatgcgggggataactgc

Cag promoter amplifiable

CMV enhancer

tcaatgacggtaaatggcccgcctggcattatgcccagtacatgaccttatgggactttcctact 325
agttactgccatttaccgggcggaccgtaatacgggtcatgtactggaataccctgaaaggatga

Cag promoter amplifiable

CMV enhancer

SnaBI

tggcagtacatctacgtattagtcatcgctattaccatggtcgaggtgagccccacgttctgctt 390
accgtcatgtagatgcataatcagtagcgataatggtaccagctccactcggggtgcaagacgaa

Cag promoter amplifiable

chicken β-actin promoter

CMV enhancer

cactctccccatctcccccccctccccaccccaattttgtatttatttattttttaattatttt 455
gtgagaggggtagaggggggggaggggtggggttaaaacataaataaataaaaaattaataaaa

Cag promoter amplifiable

chicken β-actin promoter

gtgcagcgatgggggcgggggggggggggggggcgcgcgccaggcggggcggggcggggcgagggg 520
cacgtcgctacccccgcccccccccccccccccgcgcgcggtccgccccgccccgccccgctcccc

Cag promoter amplifiable

chicken β-actin promoter

cggggcggggcgaggcggagaggtgcggcggcagccaatcagagcggcgcgctccgaaagtttcc 585
gccccgccccgctccgcctctccacgccgccgtcggttagtctcgccgcgcgaggctttcaaagg

Cag promoter amplifiable

chicken β-actin promoter

ttttatggcgaggcggcggcggcggcggccctataaaaagcgaagcgcgcggcgggcggggagtc 650
aaaataccgctccgccgccgccgccgccgggatatttttcgcttcgcgcgccgcccgcccctcag

Cag promoter amplifiable

chicken β-actin promoter

gctgcgacgctgccttcgccccgtgccccgctccgccgccgcctcgcgccgcccgccccggctct 715
cgacgctgcgacggaagcggggcacggggcgaggcggcggcggagcgcggcgggcggggccgaga

Cag promoter amplifiable

gactgaccgcgttactcccacaggtgagcgggcgggacggcccttctcctccgggctgtaattag 780
ctgactggcgcaatgagggtgtccactcgcccgccctgccgggaagaggaggcccgacattaatc

Cag promoter amplifiable

* E W L H A P P V A R R R R A T I L

cgcttggtttaatgacggcttgtttcttttctgtggctgcgtgaaagccttgaggggctccggga 845
gcgaaccaaattactgccgaacaaagaaaagacaccgacgcactttcggaactccccgaggccct

Cag promoter amplifiable

A Q N L S P K N R K Q P Q T F A K L P E P L

gggccctttgtgcggggggagcggctcggggggtgcgtgcgtgtgtgtgtgcgtggggagcgccg 910
cccgggaaacacgcccccctcgccgagcccccccacgcacgcacacacacacgcacccctcgcggc

Cag promoter amplifiable

A R Q A P P A A R P T R A H T H A H P A G

```
cgtgcggctccgcgctgcccggcggctgtgagcgctgcgggcgcggcgcggggctttgtgcgctc   975
gcacgccgaggcgcgacgggccgccgacactcgcgacgcccgcgccgcgccccgaaacacgcgag
Cag promoter amplifiable
R  A  A  G  R  Q  G  A  A  T  L  A  A  P  A  A  R  P  K  T  R  E

cgcagtgtgcgcgaggggagcgcggccgggggcggtgccccgcggtgcggggggggctgcgaggg   1040
gcgtcacacgcgctcccctcgcgccggcccccgccacggggcgccacgcccccccccgacgctccc
Cag promoter amplifiable
A  T  H  A  L  P  A  R  G  P  A  T  G  R  P  A  P  P  A  A  L  P

gaacaaaggctgcgtgcggggtgtgtgcgtgggggggtgagcagggggtgtgggcgcgtcggtcg   1105
cttgtttccgacgcacgccccacacacgcacccccccactcgtcccccacacccgcgcagccagc
Cag promoter amplifiable
V  F  A  A  H  P  T  H  A  H  P  P  S  C  P  T  H  A  R  R  D

BlpI
ggctgcaacccccctgcacccccctccccgagttgctgagcacggcccggcttcgggtgcgggg   1170
ccgacgttgggggggacgtggggggaggggctcaacgactcgtgccgggccgaagcccacgcccc
Cag promoter amplifiable
P  Q  L  G  G  Q  V  G  R  G  S  N  S  L  V  A  R  S  R  T  R  P

BsiWI, PaqCI
ctccgtacggggcgtggcgcggggctcgccgtgccgggcgggggtggcggcaggtgggggtgcc   1235
gaggcatgccccgcaccgcgccccgagcggcacggcccgcccccaccgccgtccacccccacgg
Cag promoter amplifiable
E  T  R  P  T  A  R  P  E  G  H  R  A  P  P  P  P  L  H  P  H  R

gggcggggcggggccgcctcgggccggggagggctcgggggagggcgcggcggcccccggagcg   1300
cccgccccgccccggcggagcccggcccctcccgagcccccctcccgcgccgccgggggcctcgc
Cag promoter amplifiable
A  P  R  P  R  R  P  G  P  L  A  R  P  L  P  A  A  A  G  P  A

MreI
SgrAI
ccggcggctgtcgaggcgcggcgagccgcagccattgccttttatggtaatcgtgcgagagggcg   1365
ggccgccgacagctccgcgccgctcggcgtcggtaacggaaaataccattagcacgctctcccgc
Cag promoter amplifiable
G  A  A  T  S  A  R  R  A  A  M
```

```
cagggacttcctttgtcccaaatctgtgcggagccgaaatctgggaggcgccgccgcacccccctc   1430
gtccctgaaggaaacagggtttagacacgcctcggctttagaccctccgcggcggcgtggggggag
```
Cag promoter amplifiable

```
tagcgggcgcggggcgaagcggtgcggcgccggcaggaaggaaatgggcggggagggccttcgtg   1495
atcgcccgcgccccgcttcgccacgccgcggccgtccttcctttacccgcccctcccggaagcac
```
Cag promoter amplifiable

```
cgtcgccgcgccgccgtccccttctccctctccagcctcggggctgtccgcgggggggacggctgc   1560
gcagcggcgcggcggcaggggaagagggagaggtcggagccccgacaggcgccccccctgccgacg
```
Cag promoter amplifiable

XbaI

```
cttcggggggggacggggcagggcggggttcggcttctggcgtgtgaccggcggctctagagcctc   1625
gaagcccccccctgccccgtcccgccccaagccgaagaccgcacactggccgccgagatctcggag
```
Cag promoter amplifiable

```
tgctaaccatgttcatgccttcttctttttcctacagctcctgggcaacgtgctggttattgtgc   1690
acgattggtacaagtacggaagaagaaaaaggatgtcgaggacccgttgcacgaccaataacacg
```
Cag promoter amplifiable

```
tgtctcatcattttggcaaagaattgatttgataccgcgggcGCCACCatggtgtctaagggcga   1755
acagagtagtaaaaccgtttcttaactaaactatggcgcccgCGGTGGtaccacagattcccgct
```
Cag promoter amplifiable | kozak | EGFP

M V S K G E →

Eco53kI **SacI**

```
agagctCTTCACCGGGGTGGTGCCCATCCTGGTCGAGCTGGACGGCGACGTAAACGGCCACAAGT   1820
tctcgaGAAGTGGCCCCACCACGGGTAGGACCAGCTCGACCTGCCGCTGCATTTGCCGGTGTTCA
```
EGFP

nonsense mutation G>C

E L F T G V V P I L V E L D G D V N G H K →

```
TCAGCGTGTCCGGCGAGGGCGAGGGCGATGCCACCTACGGCAAGCTGACCCTGAAGTTCATCTGC   1885
AGTCGCACAGGCCGCTCCCGCTCCCGCTACGGTGGATGCCGTTCGACTGGGACTTCAAGTAGACG
```
EGFP

F S V S G E G E G D A T Y G K L T L K F I C →

ACCACCGGCAAGCTGCCCGTGCCCTGGCCCACCCTCGTGACCACCCTGACCTACGGCGTGCAGTG 1950
TGGTGGCCGTTCGACGGGCACGGGACCGGGTGGGAGCACTGGTGGGACTGGATGCCGCACGTCAC
EGFP
T T G K L P V P W P T L V T T L T Y G V Q C

CTTCAGCCGCTACCCCGACCACATGAAGCAGCACGACTTCTTCAAGTCCGCCATGCCCGAAGGCT 2015
GAAGTCGGCGATGGGGCTGGTGTACTTCGTCGTGCTGAAGAAGTTCAGGCGGTACGGGCTTCCGA
EGFP
F S R Y P D H M K Q H D F F K S A M P E G

ACGTCCAGGAGCGCACCATCTTCTTCAAGGACGACGGCAACTACAAGACCCGCGCCGAGGTGAAG 2080
TGCAGGTCCTCGCGTGGTAGAAGAAGTTCCTGCTGCCGTTGATGTTCTGGGCGCGGCTCCACTTC
EGFP
Y V Q E R T I F F K D D G N Y K T R A E V K

TTCGAGGGCGACACCCTGGTGAACCGCATCGAGCTGAAGGGCATCGACTTCAAGGAGGACGGCAA 2145
AAGCTCCCGCTGTGGGACCACTTGGCGTAGCTCGACTTCCCGTAGCTGAAGTTCCTCCTGCCGTT
EGFP
F E G D T L V N R I E L K G I D F K E D G N

CATCCTGGGGCACAAGCTGGAGTACAACTACAACAGCCACAACGTCTATATCATGGCCGACAAGC 2210
GTAGGACCCCGTGTTCGACCTCATGTTGATGTTGTCGGTGTTGCAGATATAGTACCGGCTGTTCG
EGFP
I L G H K L E Y N Y N S H N V Y I M A D K

AGAAGAACGGCATCAAGGTGAACTTCAAGATCCGCCACAACATCGAGGACGGCAGCGTGCAGCTC 2275
TCTTCTTGCCGTAGTTCCACTTGAAGTTCTAGGCGGTGTTGTAGCTCCTGCCGTCGCACGTCGAG
EGFP
Q K N G I K V N F K I R H N I E D G S V Q L

GCCGACCACTACCAGCAGAACACCCCCATCGGCGACGGCCCCGTGCTGCTGCCCGACAACCACTA 2340
CGGCTGGTGATGGTCGTCTTGTGGGGGTAGCCGCTGCCGGGGCACGACGACGGGCTGTTGGTGAT
EGFP
A D H Y Q Q N T P I G D G P V L L P D N H Y

CCTGAGCACCCAGTCCGCCCTGAGCAAAGACCCCAACGAGAAGCGCGATCACATGGTCCTGCTGG 2405
GGACTCGTGGGTCAGGCGGGACTCGTTTCTGGGGTTGCTCTTCGCGCTAGTGTACCAGGACGACC

EGFP

L S T Q S A L S K D P N E K R D H M V L L

BsrGI EcoRI PstI SalI

AGTTCGTGACCGCCGCCGGGATCACCTTAGGCATGGACGAGCTGTACAAATAAGAATTCTGCAGT 2470
TCAAGCACTGGCGGCGGCCCTAGTGGAATCCGTACCTGCTCGACATGTTTATTCTTAAGACGTCA

EGFP | MCS

REC1

synonymous mutations to avoid primer dimers

change to AAA

E F V T A A G I T L G M D E L Y K *

HincII Acc65I XmaI TspMI AccI KpnI SmaI BamHI

CGACGGTACCGCGGGCCCGGGATCCAGATCGGAAGAGCGTCGTGTAGGGAAAAACTTGTTTATTG 2535
GCTGCCATGGCGCCCGGGCCCTAGGTCTAGCCTTCTCGCAGCACATCCCTTTTTGAACAAATAAC

MCS | adapted from illumina read1 primer | SV40 poly(A) signal

REC2

CAGCTTATAATGGTTACAAATAAAGCAATAGCATCACAAATTTCACAAATAAAGCATTTTTTTCA 2600
GTCGAATATTACCAATGTTTATTTCGTTATCGTAGTGTTTAAAGTGTTTATTTCGTAAAAAAAGT

SV40 poly(A) signal

BsmI MluI

CTGCATTCTAGTTGTGGTTTGTCCAAACTCATCAATGTATCTTAACGCGTAAATTGTAAGCGTTA 2665
GACGTAAGATCAACACCAAACAGGTTTGAGTAGTTACATAGAATTGCGCATTTAACATTCGCAAT

SV40 poly(A) signal | f1 ori

ATATTTTGTTAAAATTCGCGTTAAATTTTTGTTAAATCAGCTCATTTTTTAACCAATAGGCCGAA 2730
TATAAAACAATTTTAAGCGCAATTTAAAAACAATTTAGTCGAGTAAAAAATTGGTTATCCGGCTT

f1 ori

ATCGGCAAAATCCCTTATAAATCAAAAGAATAGACCGAGATAGGGTTGAGTGTTGTTCCAGTTTG 2795
TAGCCGTTTTAGGGAATATTTAGTTTTCTTATCTGGCTCTATCCCAACTCACAACAAGGTCAAAC

f1 ori

GAACAAGAGTCCACTATTAAAGAACGTGGACTCCAACGTCAAAGGGCGAAAAACCGTCTATCAGG 2860
CTTGTTCTCAGGTGATAATTTCTTGCACCTGAGGTTGCAGTTTCCCGCTTTTTGGCAGATAGTCC
f1 ori

DraIII
GCGATGGCCCACTACGTGAACCATCACCCTAATCAAGTTTTTTGGGGTCGAGGTGCCGTAAAGCA 2925
CGCTACCGGGTGATGCACTTGGTAGTGGGATTAGTTCAAAAAACCCCAGCTCCACGGCATTTCGT
f1 ori

CTAAATCGGAACCCTAAAGGGAGCCCCCGATTTAGAGCTTGACGGGGAAAGCCGGCGAACGTGGC 2990
GATTTAGCCTTGGGATTTCCCTCGGGGGCTAAATCTCGAACTGCCCCTTTCGGCCGCTTGCACCG
f1 ori

GAGAAAGGAAGGGAAGAAAGCGAAAGGAGCGGGCGCTAGGGCGCTGGCAAGTGTAGCGGTCACGC 3055
CTCTTTCCTTCCCTTCTTTCGCTTTCCTCGCCCGCGATCCCGCGACCGTTCACATCGCCAGTGCG
f1 ori

TGCGCGTAACCACCACACCCGCCGCGCTTAATGCGCCGCTACAGGGCGCGTCAGGTGGCACTTTT 3120
ACGCGCATTGGTGGTGTGGGCGGCGCGAATTACGCGGCGATGTCCCGCGCAGTCCACCGTGAAAA
f1 ori

CGGGGAAATGTGCGCGGAACCCCTATTTGTTTATTTTTCTAAATACATTCAAATATGTATCCGCT 3185
GCCCCTTTACACGCGCCTTGGGGATAAACAAATAAAAAGATTTATGTAAGTTTATACATAGGCGA
AmpR promoter

CATGAGACAATAACCCTGATAAATGCTTCAATAATATTGAAAAAGGAAGAGTCCTGAGGCGGAAA 3250
GTACTCTGTTATTGGGACTATTTACGAAGTTATTATAACTTTTTCCTTCTCAGGACTCCGCCTTT
AmpR promoter
SV40 promoter

GAACCAGCTGTGGAATGTGTGTCAGTTAGGGTGTGGAAAGTCCCCAGGCTCCCCAGCAGGCAGAA 3315
CTTGGTCGACACCTTACACACAGTCAATCCCACACCTTTCAGGGGTCCGAGGGGTCGTCCGTCTT
SV40 promoter

CsiI
SexAI *
GTATGCAAAGCATGCATCTCAATTAGTCAGCAACCAGGTGTGGAAAGTCCCCAGGCTCCCCAGCA 3380
CATACGTTTCGTACGTAGAGTTAATCAGTCGTTGGTCCACACCTTTCAGGGGTCCGAGGGGTCGT
SV40 promoter

GGCAGAAGTATGCAAAGCATGCATCTCAATTAGTCAGCAACCATAGTCCCGCCCCTAACTCCGCC 3445
CCGTCTTCATACGTTTCGTACGTAGAGTTAATCAGTCGTTGGTATCAGGGCGGGGATTGAGGCGG
SV40 promoter

CATCCCGCCCCTAACTCCGCCCAGTTCCGCCCATTCTCCGCCCCATGGCTGACTAATTTTTTTTA 3510
GTAGGGCGGGGATTGAGGCGGGTCAAGGCGGGTAAGAGGCGGGGTACCGACTGATTAAAAAAAAT
SV40 promoter
SV40 ori

SfiI
TTTATGCAGAGGCCGAGGCCGCCTCGGCCTCTGAGCTATTCCAGAAGTAGTGAGGAGGCTTTTTT 3575
AAATACGTCTCCGGCTCCGGCGGAGCCGGAGACTCGATAAGGTCTTCATCACTCCTCCGAAAAAA
SV40 promoter
SV40 ori

ClaI *
BspDI *
StuI
GGAGGCCTAGGCTTTTGCAAAGATCGATCAAGAGACAGGATGAGGATCGTTTCGCATGATTGAAC 3640
CCTCCGGATCCGAAAACGTTTCTAGCTAGTTCTCTGTCCTACTCCTAGCAAAGCGTACTAACTTG
1
M I E
SV40 promoter
NeoR/KanR
SV40 ori
M I E

AAGATGGATTGCACGCAGGTTCTCCGGCCGCTTGGGTGGAGAGGCTATTCGGCTATGACTGGGCA 3705
TTCTACCTAACGTGCGTCCAAGAGGCCGGCGAACCCACCTCTCCGATAAGCCGATACTGACCCGT
5 10 15 20 25
Q D G L H A G S P A A W V E R L F G Y D W A
NeoR/KanR
Q D G L H A G S P A A W V E R L F G Y D W A

CAACAGACAATCGGCTGCTCTGATGCCGCCGTGTTCCGGCTGTCAGCGCAGGGGCGCCCGGTTCT 3770
GTTGTCTGTTAGCCGACGAGACTACGGCGGCACAAGGCCGACAGTCGCGTCCCCGCGGGCCAAGA
30 35 40 45
Q Q T I G C S D A A V F R L S A Q G R P V L
NeoR/KanR
Q Q T I G C S D A A V F R L S A Q G R P V L

TTTTGTCAAGACCGACCTGTCCGGTGCCCTGAATGAACTGCAAGACGAGGCAGCGCGGCTATCGT 3835
AAAACAGTTCTGGCTGGACAGGCCACGGGACTTACTTGACGTTCTGCTCCGTCGCGCCGATAGCA
50 55 60 65
F V K T D L S G A L N E L Q D E A A R L S
NeoR/KanR
F V K T D L S G A L N E L Q D E A A R L S

MscI FspI PflFI Tth111I
GGCTGGCCACGACGGGCGTTCCTTGCGCAGCTGTGCTCGACGTTGTCACTGAAGCGGGAAGGGAC 3900
CCGACCGGTGCTGCCCGCAAGGAACGCGTCGACACGAGCTGCAACAGTGACTTCGCCCTTCCCTG
70 75 80 85 90
W L A T T G V P C A A V L D V V T E A G R D
NeoR/KanR
W L A T T G V P C A A V L D V V T E A G R D

TGGCTGCTATTGGGCGAAGTGCCGGGGCAGGATCTCCTGTCATCTCACCTTGCTCCTGCCGAGAA 3965
ACCGACGATAACCCGCTTCACGGCCCCGTCCTAGAGGACAGTAGAGTGGAACGAGGACGGCTCTT
95 100 105 110
W L L L G E V P G Q D L L S S H L A P A E K
NeoR/KanR
W L L L G E V P G Q D L L S S H L A P A E K

AGTATCCATCATGGCTGATGCAATGCGGCGGCTGCATACGCTTGATCCGGCTACCTGCCCATTCG 4030
TCATAGGTAGTACCGACTACGTTACGCCGCCGACGTATGCGAACTAGGCCGATGGACGGGTAAGC
115 120 125 130
V S I M A D A M R R L H T L D P A T C P F
NeoR/KanR
V S I M A D A M R R L H T L D P A T C P F

ACCACCAAGCGAAACATCGCATCGAGCGAGCACGTACTCGGATGGAAGCCGGTCTTGTCGATCAG 4095
TGGTGGTTCGCTTTGTAGCGTAGCTCGCTCGTGCATGAGCCTACCTTCGGCCAGAACAGCTAGTC
135 140 145 150 155
D H Q A K H R I E R A R T R M E A G L V D Q
NeoR/KanR
D H Q A K H R I E R A R T R M E A G L V D Q

GATGATCTGGACGAAGAGCATCAGGGGCTCGCGCCAGCCGAACTGTTCGCCAGGCTCAAGGCGAG 4160
CTACTAGACCTGCTTCTCGTAGTCCCCGAGCGCGGTCGGCTTGACAAGCGGTCCGAGTTCCGCTC
160 165 170 175
D D L D E E H Q G L A P A E L F A R L K A S
NeoR/KanR
D D L D E E H Q G L A P A E L F A R L K A S

CATGCCCGACGGCGAGGATCTCGTCGTGACCCATGGCGATGCCTGCTTGCCGAATATCATGGTGG 4225
GTACGGGCTGCCGCTCCTAGAGCAGCACTGGGTACCGCTACGGACGAACGGCTTATAGTACCACC

180 185 190 195
M P D G E D L V V T H G D A C L P N I M V
NeoR/KanR
M P D G E D L V V T H G D A C L P N I M V

RsrII
AAAATGGCCGCTTTTCTGGATTCATCGACTGTGGCCGGCTGGGTGTGGCGGACCGCTATCAGGAC 4290
TTTTACCGGCGAAAAGACCTAAGTAGCTGACACCGGCCGACCCACACCGCCTGGCGATAGTCCTG

200 205 210 215 220
E N G R F S G F I D C G R L G V A D R Y Q D
NeoR/KanR
E N G R F S G F I D C G R L G V A D R Y Q D

ATAGCGTTGGCTACCCGTGATATTGCTGAAGAGCTTGGCGGCGAATGGGCTGACCGCTTCCTCGT 4355
TATCGCAACCGATGGGCACTATAACGACTTCTCGAACCGCCGCTTACCCGACTGGCGAAGGAGCA

225 230 235 240
I A L A T R D I A E E L G G E W A D R F L V
NeoR/KanR
I A L A T R D I A E E L G G E W A D R F L V

GCTTTACGGTATCGCCGCTCCCGATTCGCAGCGCATCGCCTTCTATCGCCTTCTTGACGAGTTCT 4420
CGAAATGCCATAGCGGCGAGGGCTAAGCGTCGCGTAGCGGAAGATAGCGGAAGAACTGCTCAAGA

245 250 255 260
L Y G I A A P D S Q R I A F Y R L L D E F
NeoR/KanR
L Y G I A A P D S Q R I A F Y R L L D E F

BstBI
TCTGAGCGGGACTCTGGGGTTCGAAATGACCGACCAAGCGACGCCCAACCTGCCATCACGAGATT 4485
AGACTCGCCCTGAGACCCCAAGCTTTACTGGCTGGTTCGCTGCGGGTTGGACGGTAGTGCTCTAA

265
F *
NeoR/KanR
F *

TCGATTCCACCGCCGCCTTCTATGAAAGGTTGGGCTTCGGAATCGTTTTCCGGGACGCCGGCTGG 4550
AGCTAAGGTGGCGGCGGAAGATACTTTCCAACCCGAAGCCTTAGCAAAAGGCCCTGCGGCCGACC

ATGATCCTCCAGCGCGGGGATCTCATGCTGGAGTTCTTCGCCCACCCTAGGGGGAGGCTAACTGA 4615
TACTAGGAGGTCGCGCCCCTAGAGTACGACCTCAAGAAGCGGGTGGGATCCCCCTCCGATTGACT

AACACGGAAGGAGACAATACCGGAAGGAACCCGCGCTATGACGGCAATAAAAAGACAGAATAAAA 4680
TTGTGCCTTCCTCTGTTATGGCCTTCCTTGGGCGCGATACTGCCGTTATTTTTCTGTCTTATTTT
HSV TK poly(A) signal

CGCACGGTGTTGGGTCGTTTGTTCATAAACGCGGGGTTCGGTCCCAGGGCTGGCACTCTGTCGAT 4745
GCGTGCCACAACCCAGCAAACAAGTATTTGCGCCCCAAGCCAGGGTCCCGACCGTGAGACAGCTA
HSV TK poly(A) signal

BsaI

ACCCCACCGAGACCCCATTGGGGCCAATACGCCCGCGTTTCTTCCTTTTCCCCACCCCACCCCCC 4810
TGGGGTGGCTCTGGGGTAACCCCGGTTATGCGGGCGCAAAGAAGGAAAAGGGGTGGGGTGGGGGG

AAGTTCGGGTGAAGGCCCAGGGCTCGCAGCCAACGTCGGGGCGGCAGGCCCTGCCATAGCCTCAG 4875
TTCAAGCCCACTTCCGGGTCCCGAGCGTCGGTTGCAGCCCCGCCGTCCGGGACGGTATCGGAGTC

GTTACTCATATATACTTTAGATTGATTTAAAACTTCATTTTTAATTTAAAAGGATCTAGGTGAAG 4940
CAATGAGTATATATGAAATCTAACTAAATTTTGAAGTAAAAATTAAATTTTCCTAGATCCACTTC

ATCCTTTTTGATAATCTCATGACCAAAATCCCTTAACGTGAGTTTTCGTTCCACTGAGCGTCAGA 5005
TAGGAAAAACTATTAGAGTACTGGTTTTAGGGAATTGCACTCAAAAGCAAGGTGACTCGCAGTCT

CCCCGTAGAAAAGATCAAAGGATCTTCTTGAGATCCTTTTTTTCTGCGCGTAATCTGCTGCTTGC 5070
GGGGCATCTTTTCTAGTTTCCTAGAAGAACTCTAGGAAAAAAAGACGCGCATTAGACGACGAACG
ori

AAACAAAAAAACCACCGCTACCAGCGGTGGTTTGTTTGCCGGATCAAGAGCTACCAACTCTTTTT 5135
TTTGTTTTTTTGGTGGCGATGGTCGCCACCAAACAAACGGCCTAGTTCTCGATGGTTGAGAAAAA
ori

CCGAAGGTAACTGGCTTCAGCAGAGCGCAGATACCAAATACTGTCCTTCTAGTGTAGCCGTAGTT 5200
GGCTTCCATTGACCGAAGTCGTCTCGCGTCTATGGTTTATGACAGGAAGATCACATCGGCATCAA
ori

AGGCCACCACTTCAAGAACTCTGTAGCACCGCCTACATACCTCGCTCTGCTAATCCTGTTACCAG 5265
TCCGGTGGTGAAGTTCTTGAGACATCGTGGCGGATGTATGGAGCGAGACGATTAGGACAATGGTC
ori

TGGCTGCTGCCAGTGGCGATAAGTCGTGTCTTACCGGGTTGGACTCAAGACGATAGTTACCGGAT 5330
ACCGACGACGGTCACCGCTATTCAGCACAGAATGGCCCAACCTGAGTTCTGCTATCAATGGCCTA
ori

ApaLI
AAGGCGCAGCGGTCGGGCTGAACGGGGGGTTCGTGCACACAGCCCAGCTTGGAGCGAACGACCTA 5395
TTCCGCGTCGCCAGCCCGACTTGCCCCCCAAGCACGTGTGTCGGGTCGAACCTCGCTTGCTGGAT
ori

CACCGAACTGAGATACCTACAGCGTGAGCTATGAGAAAGCGCCACGCTTCCCGAAGGGAGAAAGG 5460
GTGGCTTGACTCTATGGATGTCGCACTCGATACTCTTTCGCGGTGCGAAGGGCTTCCCTCTTTCC
ori

CGGACAGGTATCCGGTAAGCGGCAGGGTCGGAACAGGAGAGCGCACGAGGGAGCTTCCAGGGGGA 5525
GCCTGTCCATAGGCCATTCGCCGTCCCAGCCTTGTCCTCTCGCGTGCTCCCTCGAAGGTCCCCCT
ori

AACGCCTGGTATCTTTATAGTCCTGTCGGGTTTCGCCACCTCTGACTTGAGCGTCGATTTTTGTG 5590
TTGCGGACCATAGAAATATCAGGACAGCCCAAAGCGGTGGAGACTGAACTCGCAGCTAAAAACAC
ori

ATGCTCGTCAGGGGGGCGGAGCCTATGGAAAAACGCCAGCAACGCGGCCTTTTTACGGTTCCTGG 5655
TACGAGCAGTCCCCCCGCCTCGGATACCTTTTTGCGGTCGTTGCGCCGGAAAAATGCCAAGGACC
ori

PciI
CCTTTTGCTGGCCTTTTGCTCACATGTTCTTTCCTGCGTTATCCCCTGATTCTGTGGATAACCGT 5720
GGAAAACGACCGGAAAACGAGTGTACAAGAAAGGACGCAATAGGGGACTAAGACACCTATTGGCA

ATTACCGCCATGCAT 3′
••• 5735
TAATGGCGGTACGTA 5′
