# Supplementary material for: Massively parallel screen uncovers many rare 3′ UTR variants regulating mRNA abundance of cancer driver genes
Source: Nat Commun. 2024 Apr 18;15:3335. doi: 10.1038/s41467-024-46795-7 (PMC11026479; doi:10.1038/s41467-024-46795-7)
Supplement: Supplementary file 3 — Description of Additional Supplementary Files [file 41467_2024_46795_MOESM3_ESM.pdf]

Description of Additional Supplementary Files

File Name: Supplementary Data 1

Description: Variants tested by MapUTR. Sheet 1. All rare variants tested with MapUTR in HEK293 cells. Sheet 2. Functional rare variants identified by MapUTR in HEK293 cells. Sheet 3. All rare variants tested with MapUTR in HeLa cells. Sheet 4. Functional rare variants identified by MapUTR in HeLa cells. Sheet 5. All COSMIC somatic mutations tested with MapUTR in HeLa cells. Sheet 6. Functional COSMIC somatic mutations identified by MapUTR in HeLa cells. lnFC: relative activity score, natural logarithm of the fold change (alt_vs_ref), pval: pval and FDR: p-values were calculated using MPRAnalyze's two-sided likelihood ratio test and corrected using the Benjamini-Hochberg method, rare_3utr_clinical: gnomad rare variants with clinical relevance.

File Name: Supplementary Data 2

Description: List of functional rare variants in cancer driver genes. Sheet 1. 499 functional rare variants found in 267 cancer driver genes. lnFC: relative activity score. Sheet 2. List of 1,143 cancer driver genes based on three different sources, including the Integrative OncoGenomics (IntOGen) databases, Pan-Cancer Analysis of Whole Genomes (PCAWG), and the Catalogue of Somatic Mutations in Cancer (COSMIC) Cancer Gene Census tier 1 (v96).

File Name: Supplementary Data 3

Description: Functional variants found in TCGA. Sheet 1. 508 functional variants associated with gene expression outliers in TCGA. Sheet 2. All functional variants found in TCGA.

File Name: Supplementary Data 4

Description: List of primers used in this study. Sheet 1. Primers used for master plasmid cloning and DNA/Cell ratio optimization. Sheet 2. Primers used for prime editing and RT-qPCR.
