# Supplementary material for: Massively parallel screen uncovers many rare 3′ UTR variants regulating mRNA abundance of cancer driver genes
Source: Nat Commun. 2024 Apr 18;15:3335. doi: 10.1038/s41467-024-46795-7 (PMC11026479; doi:10.1038/s41467-024-46795-7)
Supplement: Supplementary file 8 — Reporting Summary [file 41467_2024_46795_MOESM8_ESM.pdf]

nature portfolio

Corresponding author(s): Xinshu Xiao

Last updated by author(s): Feb 7, 2024

Reporting Summary

Nature Portfolio wishes to improve the reproducibility of the work that we publish. This form provides structure for consistency and transparency in reporting. For further information on Nature Portfolio policies, see our Editorial Policies and the Editorial Policy Checklist.

Statistics

For all statistical analyses, confirm that the following items are present in the figure legend, table legend, main text, or Methods section.

n/a	Confirmed	
☐	☒	The exact sample size (*n*) for each experimental group/condition, given as a discrete number and unit of measurement
☐	☒	A statement on whether measurements were taken from distinct samples or whether the same sample was measured repeatedly
☐	☒	The statistical test(s) used AND whether they are one- or two-sided<br>*Only common tests should be described solely by name; describe more complex techniques in the Methods section.*
☒	☐	A description of all covariates tested
☐	☒	A description of any assumptions or corrections, such as tests of normality and adjustment for multiple comparisons
☐	☒	A full description of the statistical parameters including central tendency (e.g. means) or other basic estimates (e.g. regression coefficient) AND variation (e.g. standard deviation) or associated estimates of uncertainty (e.g. confidence intervals)
☐	☒	For null hypothesis testing, the test statistic (e.g. *F*, *t*, *r*) with confidence intervals, effect sizes, degrees of freedom and *P* value noted<br>*Give P values as exact values whenever suitable.*
☒	☐	For Bayesian analysis, information on the choice of priors and Markov chain Monte Carlo settings
☒	☐	For hierarchical and complex designs, identification of the appropriate level for tests and full reporting of outcomes
☐	☒	Estimates of effect sizes (e.g. Cohen's *d*, Pearson's *r*), indicating how they were calculated

Our web collection on statistics for biologists contains articles on many of the points above.

Software and code

Policy information about availability of computer code

Data collection	Python (version 2.7.18) was used for synthetic DNA oligonucleotide library design. Incucyte software was used to collect cell proliferation data. pegFinder (http://pegfinder.sidichenlab.org/) and pegLIT (https://peglit.liugroup.us/) were used to design epegRNAs.
Data analysis	R (version 4.2.2), Python (version 2.7.18), UMItools (version 0.5.5), Botwtie2 (version 2.3.5), MPRAnalyze (https://github.com/YosefLab/MPRAnalyze), HOMER (verson 4.11), DeepRiPe (https://github.com/ohlerlab/DeepRiPe), 4Peaks (version 1.8), CFX Maestro (version 4.1.2434.0124), GraphPad Prism (version 7) were used for data analysis.

For manuscripts utilizing custom algorithms or software that are central to the research but not yet described in published literature, software must be made available to editors and reviewers. We strongly encourage code deposition in a community repository (e.g. GitHub). See the Nature Portfolio guidelines for submitting code & software for further information.

Data

Policy information about availability of data

All manuscripts must include a data availability statement. This statement should provide the following information, where applicable:

- Accession codes, unique identifiers, or web links for publicly available datasets
- A description of any restrictions on data availability
- For clinical datasets or third party data, please ensure that the statement adheres to our policy

The rare 3' UTR variants tested in this study were obtained from gnomAD (https://gnomad.broadinstitute.org). The predicted miRNA target sites were obtained

from the TargetScan database at https://www.targetscan.org/vert_72/vert_72_data_download/All_Target_Locations.hg19.bed.zip. The gene-disease association data was obtained from the DisGeNET database at https://www.disgenet.org/downloads. The Intogen data used in this study are available at https://intogen.org/download. The Cancer Genome Atlas (TGCA) data are available under restricted access a
dhere to the National Institutes of Health (NIH) Genomic Data Sharing (GDS) policy as well as the National Cancer Institute (NCI) GDS policy; access for FPKM in cancer patients can be obtained through the Genomic Data Commons (GDC) portal (https://portal.gdc.cancer.gov/); access for genotype data can be obtained through the ICGC Data Portal (http://dcc.icgc.org/pcawg/). Processed MapUTR data generated by this study is available at https://github.com/gxiaolab/mapUTR. The GTEx data was obtained from the GTEx portal (https://www.gtexportal.org/). All raw MapUTR data are available in the GEO database under the accession code GSE232573 (https://www.ncbi.nlm.nih.gov/geo/query/acc.cgi?acc=GSE232573). Source data are provided with this paper.

Research involving human participants, their data, or biological material

Policy information about studies with human participants or human data. See also policy information about sex, gender (identity/presentation), and sexual orientation and race, ethnicity and racism.

Reporting on sex and gender	This information has not been collected.
Reporting on race, ethnicity, or other socially relevant groupings	This study did not perform race, ethnicity, or other socially relevant groupings.
Population characteristics	For TCGA data, we categorized individuals based on their genotype, gene expression, and survival metrics (progression-free interval). For GTEx data, we categorized individuals based on their genotype and gene expression.
Recruitment	This study did not recruit any participants.
Ethics oversight	n/a

Note that full information on the approval of the study protocol must also be provided in the manuscript.

Field-specific reporting

Please select the one below that is the best fit for your research. If you are not sure, read the appropriate sections before making your selection.

☒ Life sciences ☐ Behavioural & social sciences ☐ Ecological, evolutionary & environmental sciences

For a reference copy of the document with all sections, see nature.com/documents/nr-reporting-summary-flat.pdf

Life sciences study design

All studies must disclose on these points even when the disclosure is negative.

Sample size	The sample size of variants tested in MapUTR was determined by first requiring variants to have an adjusted minor allele frequency < 0.01. The remaining variants were filtered based on the cloning strategy. Thus, 3' UTR sequences that shared similarities with restriction enzyme sites or primer sequences were filtered. MapUTR was performed with three replicates. All other experiments were performed with at least 3 replicates.
Data exclusions	No data was excluded.
Replication	MapUTR was performed with three replicates. For functional studies of genome edited variants, 4-6 clones were used for each variant to avoid bias caused by off-target effects. All attempts at replication were successful.
Randomization	MapUTR variants were randomly allocated into different subpools and tested at different time-points.
Blinding	Investigators were blinded to variant outcome during data collection and analysis.

Reporting for specific materials, systems and methods

We require information from authors about some types of materials, experimental systems and methods used in many studies. Here, indicate whether each material, system or method listed is relevant to your study. If you are not sure if a list item applies to your research, read the appropriate section before selecting a response.

Materials & experimental systems

n/a	Involved in the study
☒	☐ Antibodies
☐	☒ Eukaryotic cell lines
☒	☐ Palaeontology and archaeology
☒	☐ Animals and other organisms
☒	☐ Clinical data
☒	☐ Dual use research of concern
☒	☐ Plants

Methods

n/a	Involved in the study
☒	☐ ChIP-seq
☒	☐ Flow cytometry
☒	☐ MRI-based neuroimaging

Eukaryotic cell lines

Policy information about cell lines and Sex and Gender in Research

Cell line source(s)	HeLa (female) and HEK293T (female) cells were obtained from ATCC. HEK293 (female) cells were obtained from Jing Huang's lab at UCLA.
Authentication	None of the cell line used were authenticated.
Mycoplasma contamination	HeLa cells were tested negative for mycoplasma contamination. HEK293 and HEK293T cells were not tested for mycoplasma contamination.
Commonly misidentified lines (See ICLAC register)	HEK293 and HEK293T are on the list of commonly misidentified cell lines.
